# Host-directed antiviral strategy targeting prohibitins: Mel56 suppresses influenza A virus and severe acute respiratory syndrome coronavirus 2 via modulation of antioxidant pathways and mitochondrial function

**DOI:** 10.1128/spectrum.03093-25

**Published:** 2026-05-15

**Authors:** Masaki Shoji, Rina Hashimoto, Canan G. Nebigil, Kazuto Takegawa, Itsuki Tomita, Kensuke Nakaoka, Momiji Ishikawa, Yasufumi Matsumura, Miki Nagao, Tomoyuki Esumi, Etsuhisa Takahashi, Hiroshi Kido, Yasuo Shinohara, Laurent Désaubry, Kazuo Takayama, Takashi Kuzuhara

**Affiliations:** 1 Laboratory of Biochemistry, Faculty of Pharmaceutical Sciences, Tokushima Bunri University 426811 , Tokushima, Japan; 2 Department of Synthetic Human Body System, Medical Research Laboratory, Institute of Integrated Research, Institute of Science Tokyo, Tokyo, Japan; 3 Center for iPS Cell Research and Application (CiRA), Kyoto University 12918, Kyoto, Japan; 4 Regenerative Nanomedicine Laboratory (UMR1260), INSERM-University of Strasbourg, Center of Research in Biomedicine of Strasbourg (CRBS), Strasbourg, France; 5 Institute for Genome Research, Institute of Advanced Medical Sciences, Tokushima University 13109, Tokushima, Japan; 6 Graduate School of Pharmaceutical Sciences, Tokushima University 13109, Tokushima, Japan; 7 Department of Clinical Laboratory Medicine, Graduate School of Medicine, Kyoto University 12918, Kyoto, Japan; 8 Laboratory of Medicinal Chemistry, Faculty of Pharmaceutical Sciences at Kagawa, Tokushima Bunri University, Takamatsu, Japan; 9 Division of Enzyme Chemistry, Institute for Enzyme Research, Tokushima University 13109, Tokushima, Japan; Wannan Medical University, Wuhu, Anhui Province, China; The University of Hong Kong, Pokfulam, Hong Kong

**Keywords:** influenza A virus, SARS-CoV-2, prohibitin, Mel56, triazine, host-targeted, mitochondria, melanogenin, knockdown, transcriptome

## Abstract

Respiratory viruses, such as influenza A virus (IAV) and severe acute respiratory syndrome coronavirus 2 (SARS-CoV-2), remain major global health threats, and the emergence of drug-resistant variants underscores the urgent need for host-targeted antiviral strategies. Prohibitins (PHBs), mitochondrial scaffold proteins involved in diverse cellular processes, are host factors exploited by multiple viruses. Here, we investigated the antiviral potential of two PHB-binding triazine melanogenin derivatives, Mel56 and Mel6, against IAV and SARS-CoV-2. Mel56, but not Mel6, exhibited potent anti-IAV activity. In particular, Mel56 increased cell survival, suppressed viral nucleoprotein expression, and reduced viral gene transcription in IAV-infected Madin-Darby canine kidney (MDCK) cells and A549 human lung carcinoma cells. PHB2 knockdown enhanced the inhibitory effect of Mel56, supporting the involvement of PHBs in its antiviral mechanism. Transcriptomic profiling revealed that Mel56 downregulates virus-induced immune response genes while upregulating antioxidant response-related genes, including nuclear factor erythroid 2-related factor 2 (NRF2) target genes. NRF2 activation by Mel56 was confirmed using a reporter assay. Consistent with these findings, Mel56 impaired mitochondrial ATP synthesis and electron transport in isolated rat liver mitochondria and attenuated mitochondrial membrane potential and promoted mitochondrial reactive oxygen species production in live MDCK cells. Importantly, Mel56 exhibited potent anti-SARS-CoV-2 activity in human induced pluripotent stem cell-derived lung organoids. Mel56 did not upregulate NRF2 target genes in SARS-CoV-2-infected organoids. These findings identify Mel56 as a PHB-binding compound with broad-spectrum antiviral activity and support the development of PHB-targeting ligands as a novel host-directed therapeutic strategy against respiratory viral infections.

**IMPORTANCE:**

This study identifies Mel56 as a novel host-directed antiviral compound that targets prohibitins and suppresses both influenza A virus and severe acute respiratory syndrome coronavirus 2. Mel56 exhibits broad-spectrum antiviral activity in conventional cell culture models as well as in physiologically relevant human lung organoids. Mechanistically, Mel56 upregulates antioxidant response-related genes, activates nuclear factor erythroid 2-related factor 2, impairs mitochondrial function, and enhances mitochondrial reactive oxygen species production. Notably, its effects differ depending on the cellular context, underscoring the complexity of host signaling pathways during viral infection. These findings highlight prohibitins as promising therapeutic targets and provide proof of concept for the development of host-directed strategies to combat respiratory viruses and mitigate the emergence of drug resistance.

## INTRODUCTION

Respiratory viruses, including influenza A virus (IAV) and severe acute respiratory syndrome coronavirus 2 (SARS-CoV-2), continue to pose major threats to global health. Seasonal influenza causes an estimated 3 to 5 million cases of severe illness and 290,000 to 650,000 respiratory deaths annually, according to the World Health Organization [https://www.who.int/news-room/fact-sheets/detail/influenza-(seasonal)]. The coronavirus disease 2019 (COVID-19) pandemic, caused by SARS-CoV-2, has led to over 760 million confirmed cases and nearly 7 million deaths worldwide since its emergence in late 2019 (https://www.who.int/health-topics/coronavirus#tab=tab_1). Despite the availability of several antiviral drugs targeting viral proteins, such as the influenza M2 ion channel, neuraminidase, cap-dependent endonuclease, SARS-CoV-2 3C-like protease, and RNA-dependent RNA polymerase (1–6), increasing resistance is an important issue. Many circulating influenza strains are resistant to M2 inhibitors, and reduced susceptibility to neuraminidase and endonuclease inhibitors has been reported in various influenza subtypes, including H1N1, H3N2, and H5N1 (7–10). Similarly, SARS-CoV-2 variants harboring mutations that confer resistance to SARS-CoV-2 3C-like protease inhibitors (e.g., nirmatrelvir and ensitrelvir) and RNA-dependent RNA polymerase inhibitors (e.g., remdesivir) have been documented (11–13). These trends highlight the urgent need for antiviral strategies that target host factors essential for viral replication, offering the potential for broad-spectrum efficacy and reducing the risk of resistance development.

Prohibitins (PHBs), comprising PHB1 and PHB2, are evolutionarily conserved mitochondrial scaffold proteins that interact with proteins, lipids, and nucleic acids and are involved in diverse cellular processes, including mitochondrial quality control, cell proliferation, apoptosis, and intracellular signaling (14–16). Host cell PHBs are exploited by various human viruses, including human immunodeficiency virus type 1 (17), human hepatitis C virus (18), dengue virus (19), Chikungunya virus (20, 21), influenza virus (22–25), SARS-CoV (26, 27), and SARS-CoV-2 (28, 29). In IAV-infected cells, PHBs are upregulated, and RNA interference-mediated knockdown of PHBs reduces cytoplasmic nucleoprotein (NP) accumulation and impairs viral replication (22–25). In SARS-CoV-2, proteomic studies have revealed interactions between PHBs and nonstructural proteins NSP2 and NSP6 (27–29), suggesting a possible role in viral replication and pathogenesis. Taken together, these observations suggest that PHBs are promising host-directed targets for the development of broad-spectrum antiviral agents. Our previous study identified bakuchiol (baku), a natural phenolic compound with a chiral tetra-alkylated center, as an enantiomer-specific PHB2-binding antiviral agent effective against IAV (24, 30, 31), supporting the antiviral potential of small molecules targeting PHBs.

Melanocytes, beyond their role in pigmentation, contribute to the innate immune defense in the epidermis. Toll-like receptor signaling stimulates melanogenesis and melanin transport (32), suggesting a functional link between immune signaling and pigment production. These findings suggest that melanogenic processes are connected to host antiviral responses through shared signaling components. Recent studies have characterized a series of triazine-based melanogenin derivatives, Mel6, Mel9, Mel41, and Mel56, that bind to PHBs (33, 34). Mel9, Mel41, and Mel56 promote melanogenesis and induce apoptosis in melanoma cells, whereas Mel6 inhibits melanin production in melanocytes (33, 34). These effects are mediated by PHB-dependent activation of microphthalmia-associated transcription factor (MITF) signaling via extracellular signal-regulated kinase (ERK) phosphorylation and accumulation of LC3-II, an autophagy-related protein (33, 34).

In this study, we investigated the antiviral properties of two PHB-binding triazine melanogenin derivatives, Mel56 and Mel6, against IAV and SARS-CoV-2. We found that Mel56, but not Mel6, inhibited IAV infection in Madin-Darby canine kidney (MDCK) cells and A549 human lung carcinoma cells and acted additively with PHB2 knockdown to suppress viral NP expression. Transcriptomic profiling revealed that Mel56 downregulates virus-induced immune response genes while upregulating antioxidant response-related genes, including nuclear factor erythroid 2-related factor 2 (NRF2) target genes. NRF2 activation by Mel56 was confirmed using a reporter assay. Furthermore, we demonstrated that Mel56 impairs mitochondrial ATP synthesis and electron transport in isolated rat liver mitochondria. In live MDCK cells, Mel56 attenuated mitochondrial membrane potential and promoted mitochondrial reactive oxygen species (ROS) production. Interestingly, Mel56 also inhibited SARS-CoV-2 infection in human induced pluripotent stem cell (hiPSC)-derived lung organoids but did not upregulate NRF2 target genes in SARS-CoV-2-infected organoids, suggesting context-dependent NRF2 signaling. Together, these findings identify Mel56 as a PHB-binding compound with broad-spectrum antiviral activity that induces an NRF2-mediated antioxidant response through modulation of mitochondrial function, supporting the development of PHB-targeting ligands as a novel host-directed therapeutic strategy against respiratory viral infections.

## RESULTS

### Mel56 increases the survival of MDCK cells infected with influenza A H1N1 and H3N2 viruses

The cytotoxicity of Mel6 and/or Mel56 in MDCK and A549 cells was assessed using thiazolyl blue tetrazolium bromide (MTT) and WST-8 assays (Fig. S1A through E and S2A). In medium supplemented with 10% fetal bovine serum (FBS), treatment with 20 µM–40 µM Mel56 for 72 h reduced cell viability significantly compared with that for dimethyl sulfoxide (DMSO) controls (Fig. S1B and C and S2A). Mel6 at concentrations up to 40 µM and Mel56 up to 10 µM showed no significant cytotoxicity (Fig. S1B and C and S2A). In medium supplemented with 1% bovine serum albumin (BSA), both compounds markedly reduced cell viability at concentrations between 1.25 and 40 µM after 72 h (Fig. S1D and E). These results indicate that Mel6 and Mel56 are non-cytotoxic at ≤10 µM when MDCK and A549 cells are cultured in 10% FBS-containing medium.

**Fig 1:**
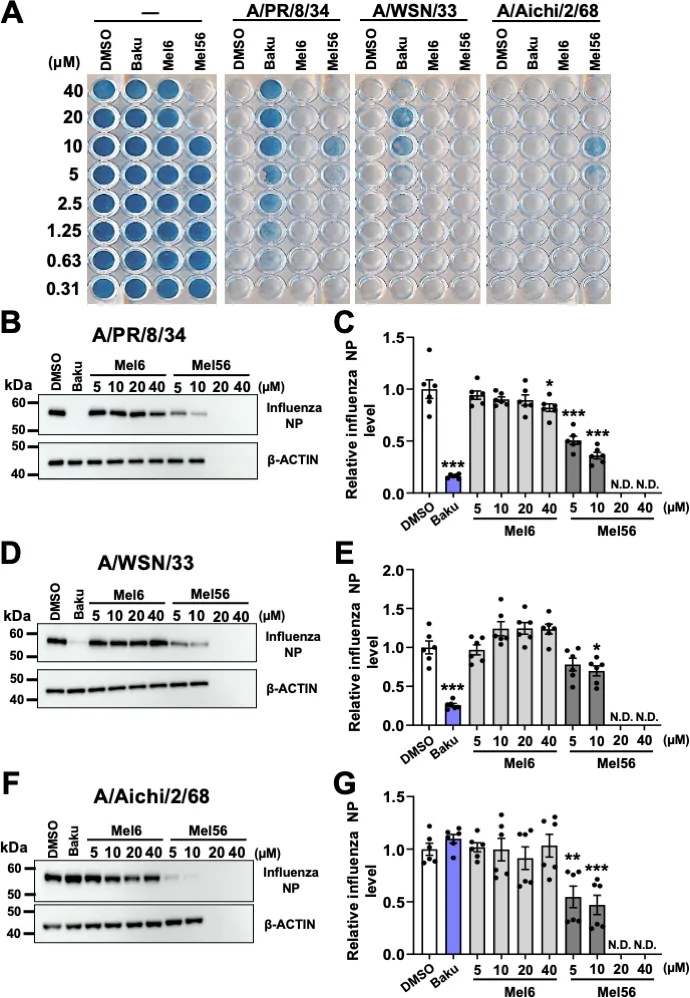
Effects of PHB ligands Mel6 and Mel56 on cell survival and viral NP expression in IAV-infected MDCK cells. (**A**) NB staining performed at 72 hpi to assess the viability of MDCK cells treated with the indicated Mel6 or Mel56 concentrations in the absence (–) or presence of influenza A/PR/8/34, A/WSN/33, or A/Aichi/2/68 viruses. DMSO (0.0031%–0.4%) served as a negative control and baku (0.8 μM–25 μM) as a positive control. Data are representative of three independent experiments. (**B–G**) WB analysis of influenza A NP expression in MDCK cells infected with A/PR/8/34 (**B and C**), A/WSN/33 (**D and E**), or A/Aichi/2/68 (**F and G**) and treated with the indicated Mel6 or Mel56 concentrations. DMSO (0.4%) served as a negative control, baku (10 μM) as a positive control, and β-actin as an internal control. NP expression was normalized to β-actin and presented relative to levels in DMSO-treated controls (set to 1). Data are shown as means ± SEM (*n* = 6) from two independent experiments. N.D., not detected. *P < 0.05, **P < 0.01, ***P < 0.001 versus DMSO, as determined using one-way ANOVA followed by Dunnett’s *post hoc* tests.

The effects of Mel6 and Mel56 on the viability of MDCK cells infected with the IAV strains A/PR/8/34 (H1N1), A/WSN/33 (H1N1), and A/Aichi/2/68 (H3N2) were evaluated using naphthol blue-black (NB) staining at 72 h post-infection (hpi). DMSO and baku served as negative and positive controls, respectively. Virus-free cells remained viable, as indicated by blue staining, at all concentrations tested, except 20 µM–40 µM Mel56 (Fig. 1A), indicating negligible cytotoxicity under these conditions. In contrast, infected cells treated with DMSO or Mel6 were unstained, indicating cell death (Fig. 1A). Treatment with 5 µM–10 µM Mel56 preserved cell viability in cells infected with A/PR/8/34 or A/Aichi/2/68, similar to the effects of baku at 0.4 µM–25 µM (A/PR/8/34) or 3.1 µM–25 µM (A/WSN/33) (Fig. 1A). These findings demonstrated that Mel56, but not Mel6, enhanced the survival of MDCK cells infected with H1N1 and H3N2 IAVs.

### Mel56 reduces influenza A NP expression and the number of infected cells

Next, we investigated whether Mel6 or Mel56 suppresses IAV NP expression. MDCK and A549 cells were infected with A/PR/8/34, A/WSN/33, or A/Aichi/2/68 in the presence of Mel6 or Mel56, and NP levels in cell lysates were analyzed by Western blotting (WB) at 24 hpi (Fig. 1B through G; Fig. S2B through G). In MDCK cells, Mel56 significantly reduced NP expression in a dose-dependent manner for all three virus strains. NP expression was suppressed at 5 µM–10 µM for A/PR/8/34 and A/Aichi/2/68 and at 10 µM for A/WSN/33 compared with levels in DMSO-treated controls (Fig. 1B through G). In A549 cells, Mel56 also significantly reduced NP expression in a dose-dependent manner for A/PR/8/34 and A/Aichi/2/68, with suppression observed at 5 µM–10 µM for A/PR/8/34 (Fig. S2B and C) and 2.5 µM–10 µM for A/Aichi/2/68 (Fig. S2F and G). Baku (10 µM) showed similar inhibitory effects in IAV-infected MDCK and A549 cells. Mel6 reduced NP expression only at 40 µM in A/PR/8/34-infected cells (Fig. 1C).

**Fig 2:**
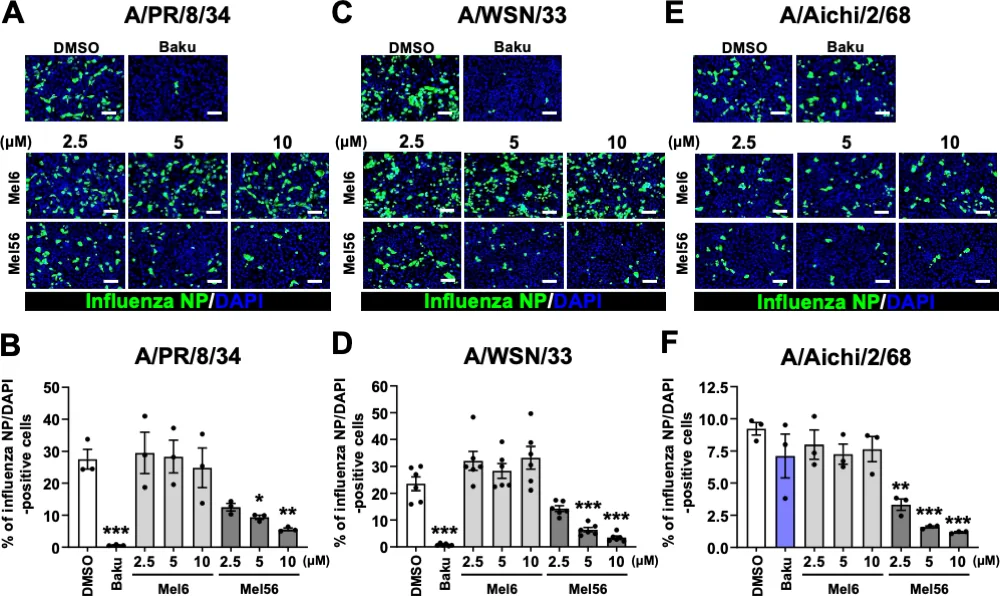
IF analysis of Mel56-mediated inhibition of IAV infection. (**A, C, and E**) Representative merged IF images showing NP-positive cells (green) and 4′,6-diamidino-2-phenylindole (DAPI)-stained nuclei (blue) in MDCK cells infected with A/PR/8/34 (**A**), A/WSN/33 (**C**), or A/Aichi/2/68 (**E**) and treated with Mel6 or Mel56 (2.5 µM–10 µM). DMSO (0.1%) was used as a negative control and baku (10 μM) as a positive control. Scale bars represent 100 µm. (**B, D, and F**) Quantification of NP-positive cells (green) as a percentage of total DAPI-stained cells (blue) in the respective experimental groups. Data are expressed as means ± SEM (*n* = 3 or 6) from two independent experiments. *P < 0.05, **P < 0.01, ***P < 0.001 versus DMSO, as determined using one-way ANOVA followed by Dunnett’s *post hoc* tests.

To further assess the inhibitory effect on viral infection, we performed immunofluorescence (IF) staining to quantify NP-positive cells at 24 hpi (Fig. 2). Mel56 (2.5 µM–10 µM) resulted in significantly lower numbers (Fig. 2A, C, and E) and percentages of NP-positive cells in cultures infected with A/PR/8/34, A/WSN/33, or A/Aichi/2/68 compared with those for cells treated with DMSO and comparable estimates to those for baku (Fig. 2B, D, and F). In contrast, Mel6 (2.5 µM–10 µM) did not alter the number of NP-positive cells. These results confirmed that Mel56 suppresses IAV infection in MDCK and A549 cells.

We evaluated the selectivity index (SI) of Mel56 against IAV infection in MDCK and A549 cells, as summarized in Table 1. SI values were calculated using CC_50_ values determined by the WST-8 assay in MDCK and A549 cells (Fig. S1C and S2A) and IC_50_ values derived from WB and IF analyses of NP expression for the A/PR/8/34, A/WSN/33, and A/Aichi/2/68 strains in the respective cell lines (Fig. 1C, E, and G; Fig. S2C, E, and G). The calculated SI values ranged from approximately 0.1 to >9.9 (Table 1).

**TABLE 1:** The SI values of Mel56 against anti-IAV activity^*a*^

Cell	Experiment	Influenza virus strain	IC_50_ (µM)	CC_50_ (µM)	SI values (CC_50_/IC_50_)
MDCK	WB analysis (viral NP expression)	A/PR/8/34	5.1	14.2	2.8
		A/WSN/33	38.8	14.2	0.4
		A/Aichi/2/68	7.6	14.2	1.9
	IF analysis (virus-infected cell number)	A/PR/8/34	2.1	14.2	6.8
		A/WSN/33	3.1	14.2	4.6
		A/Aichi/2/68	1.4	14.2	9.9
A549	WB analysis (viral NP expression)	A/PR/8/34	323.2	18.1	0.1
		A/WSN/33	NA	18.1	NA
		A/Aichi/2/68	5.1	18.1	3.6

^*a*^ The SI was calculated using the formula: SI = CC_50_/IC_50_. CC_50_ values of Mel56 were determined by the WST-8 cell viability assay using MDCK cells, A549 cells, as described above (Fig. S1C and S2A). IC_50_ values against the infection of IAV strains A/PR/8/34, A/WSN/33, and A/Aichi/2/68 were calculated based on WB and IF analyses of viral NP expression in Mel56-treated MDCK or A549 cells infected with the respective IAV strains (Fig. 1C, E, and G; Fig. S2C, E, and G). Dose–response curves were generated, and all CC_50_ and IC_50_ values were calculated. An SI value greater than 1.0 was considered indicative of greater antiviral efficacy than cytotoxicity in the respective cell model. NA indicates no activity.

### Mel56 suppresses viral and host antiviral gene expression in IAV-infected cells

**Fig 3:**
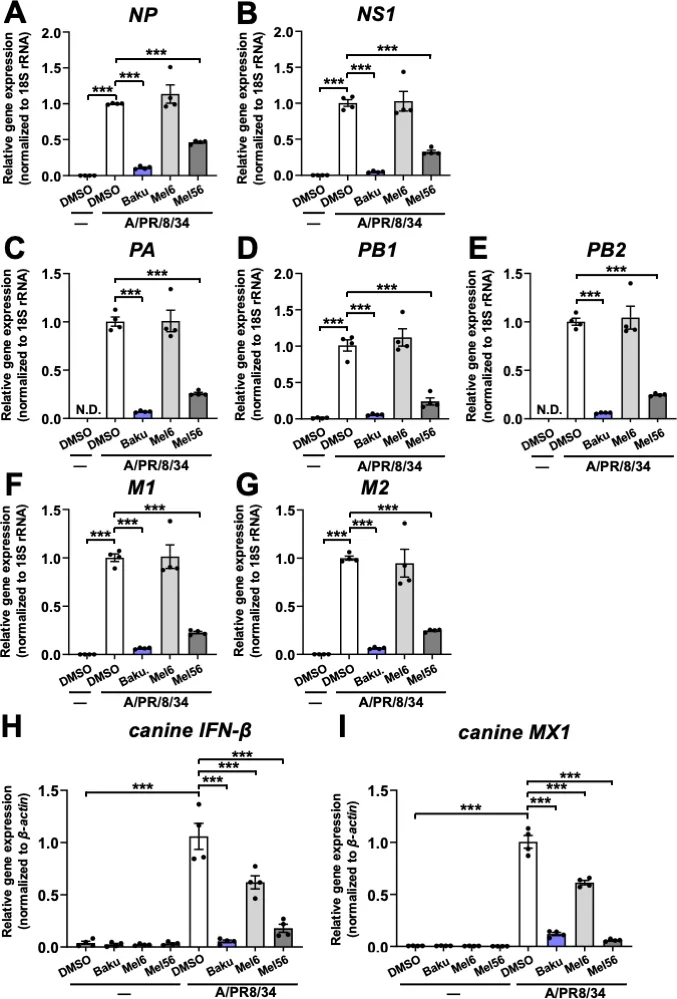
Mel56 suppresses influenza A viral and host antiviral gene expression in infected MDCK cells. (**A–G**) Relative viral gene expression levels (*NP*, *NS1*, *PA*, *PB1*, *PB2*, *M1*, and *M2*) in MDCK cells treated with Mel6 or Mel56 (10 µM) in the presence or absence (–) of A/PR/8/34 virus, analyzed using RT-qPCR. (**H and I**) Expression levels of the antiviral host genes canine *IFN-β* and *MX1* under similar conditions. DMSO (0.1%) served as a negative control and baku (10 µM) as a positive control. Gene expression was normalized to levels of *18S rRNA* or *canine β-actin* and expressed relative to DMSO-treated, virus-infected cells (set to 1). Data are presented as means ± SEM (*n* = 4) from two independent experiments. ***P < 0.001 versus DMSO, as determined using one-way ANOVA followed by Dunnett’s *post hoc* tests.

To determine whether Mel56 affects viral gene expression, we conducted reverse transcription-quantitative polymerase chain reaction (RT-qPCR) on MDCK cells treated with Mel6 or Mel56 (10 µM) and infected with A/PR/8/34 virus. Mel56 decreased the expression levels of *NP*, nonstructural protein 1 (*NS1*), polymerase subunits (*PA*, *PB1*, and *PB2*), matrix protein 1 (*M1*), and *M2* viral genes significantly at 24 hpi (*P* < 0.001, Fig. 3A through G), similar to the effects of baku.

We also examined the host antiviral responses. Mel56 reduced the expression levels of interferon-β (*IFN-β*) and MX dynamin-like GTPase 1 (*MX1*), two key antiviral genes, significantly (*P* < 0.001, Fig. 3H and I). Mel6 reduced the expression of these transcripts to a lesser extent. These findings suggest that Mel56 potently inhibits viral replication and virus-induced host immune gene expression.

### PHB proteins contribute to the anti-influenza activity of Mel56

**Fig 4:**
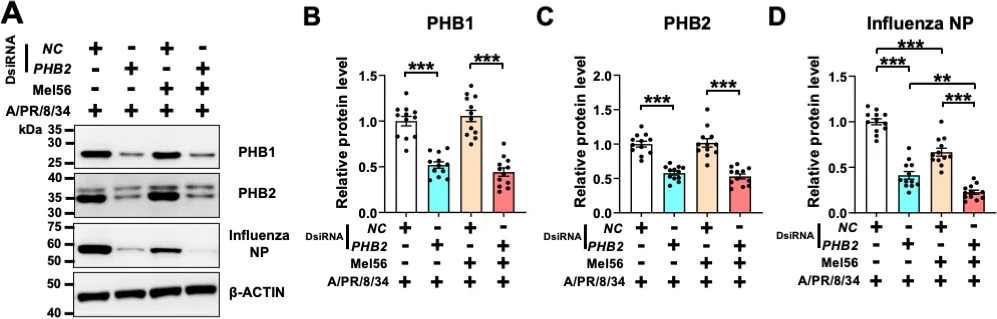
*PHB2* knockdown enhances antiviral effects of Mel56 in IAV-infected MDCK cells. (**A**) WB analysis of PHB1, PHB2, influenza NP, and β-actin in MDCK cells transfected with *PHB2* DsiRNA or negative control (*NC*) DsiRNA, treated with Mel56 (10 µM), and infected with A/PR/8/34 virus. DMSO (0.1%) was used as a negative control. (**B–D**) Quantification of PHB1 (**B**), PHB2 (**C**), and NP (**D**) protein levels, normalized to β-actin and presented relative to levels in the *NC* DsiRNA + DMSO group (set to 1). Data are presented as means ± SEM (*n* = 12) from four independent experiments. **P < 0.01, ***P < 0.001, as determined using one-way ANOVA followed by Tukey’s *post hoc* tests.

To assess the role of PHBs in Mel56-mediated antiviral activity, MDCK cells were transfected with *PHB2*-specific dicer-substrate siRNA (DsiRNA) before A/PR/8/34 virus infection and Mel56 treatment. WB analyses revealed that *PHB2* knockdown led to significant reductions in both PHB1 and PHB2 protein levels (*P* < 0.001, Fig. 4A through C). Influenza A NP expression was significantly decreased by either *PHB2* DsiRNA (*P* < 0.001) or Mel56 alone (*P* < 0.001), with greater reductions when both were combined (*P* < 0.01 and *P* < 0.001) (Fig. 4A and D), indicating an additive inhibitory effect and implicating PHBs in the antiviral mechanism of Mel56.

### Mel56-induced activation of MITF signaling is not involved in the antiviral mechanism

Mel56 activates MITF signaling via LC3-II accumulation and ERK phosphorylation, thereby promoting melanogenesis (33, 34). To determine whether MITF signaling contributes to the antiviral activity of Mel56, we assessed relative protein expression levels of PHB1 (Fig. S3B), PHB2 (Fig. S3C), LC3-I (Fig. S3D), LC3-II (Fig. S3E), ERK (Fig. S3F), p-ERK/ERK (Fig. S3G), MITF (Fig. S3H), tyrosinase (Fig. S3I), and influenza NP (Fig. S3J). Mel56 and Mel6 increased LC3-II and MITF levels (Fig. S3E and H), indicating the activation of MITF signaling. However, only Mel56 reduced NP expression (Fig. S3J), suggesting that MITF signaling was not responsible for the anti-influenza activity of Mel56.

### Mel56 reduces virus-induced host immune response genes and induces antioxidant response genes via NRF2 activation

To identify the genes involved in the anti-influenza activity of Mel56, we performed an RNA sequencing (RNA-seq) analysis of IAV-infected MDCK cells treated with Mel56. A total of 1,767 differentially expressed genes (DEGs) were identified using |fold change (FC)| ≥2 and raw *P*-value <0.05 as thresholds for the comparison between Mel56-treated and DMSO-treated MDCK cells infected with A/PR/8/34 virus (Fig. S4). Among these DEGs, 1,062 were upregulated and 705 were downregulated (≥2 FC) in response to Mel56 treatment (Fig. S4B, Tables S3 and S4).

Gene Ontology (GO) and Kyoto Encyclopedia of Genes and Genomes (KEGG) enrichment analyses revealed that the downregulated genes were significantly enriched in the “immune response” and “immune system process” categories, which ranked among the top 2 Biological Process (BP) terms (Fig. S5A). Among these, the top 7 downregulated genes—2′-5′-oligoadenylate synthetase 2 (*OAS2*), chemokine (C-X-C motif) ligand 10 (*CXCL10*), *MX2*, bone marrow stromal cell antigen 2 (*BST2*), ISG15 ubiquitin-like modifier (*ISG15*), DExH-box helicase 58, transcript variant X3 (*DHX58*), and interferon-induced protein 35—were identified based on the criteria of |FC| ≥ 2 and transcripts per million (TPM) ≥100 (*P* < 0.001, Fig. S5B). Similarly, a KEGG pathway analysis identified “Influenza A” and “Herpes simplex virus 1 infection” categories as the top 2 enriched pathways (Fig. S5C). The top 7 downregulated genes, *OAS2*, *CXCL10*, *MX2*, *MX1*, *BST2*, DExD/H-box helicase 58 (*DDX58*), and TNF superfamily member 10 (*TNFSF10*), were selected based on the same criteria (*P* < 0.001, Fig. S5D). Genes involved in these pathways are typically upregulated during influenza virus infection. Additionally, other viral infection-related categories, including “Hepatitis C,” “Epstein–Barr virus infection,” and “Coronavirus disease–COVID-19,” were ranked among the top 10 categories in the KEGG pathway analysis (Fig. S5C).

**Fig 5:**
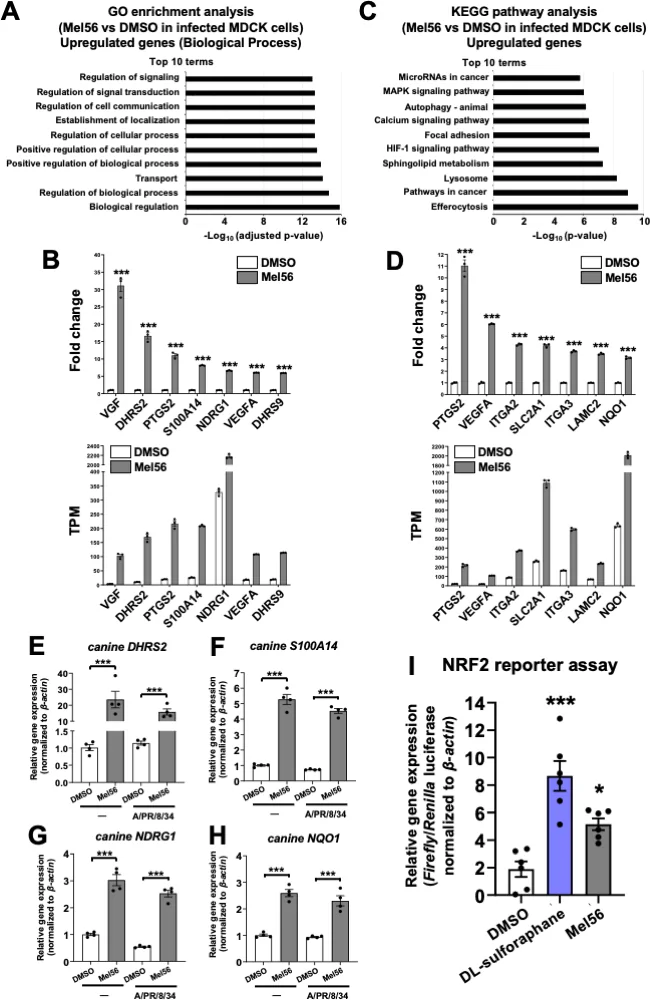
Mel56 induces antioxidant response gene upregulation and activates NRF2. (**A−H**) Transcriptomic analysis of upregulated DEGs in Mel56-treated MDCK cells infected with IAV.(**A**) GO enrichment analysis of 1,062 upregulated DEGs in Mel56-treated MDCK cells infected with A/PR/8/34 identified by RNA-seq. Top 10 enriched BP terms are shown as −log₁₀ (adjusted *P*-value). (**B**) Upper: fold change of top 7 upregulated genes in “Biological regulation” and “Regulation of biological process” BP categories, relative to DMSO (set to 1). Lower: TPM values of the same genes. Data are shown as mean ± SEM (*n* = 3). ***P < 0.001 versus DMSO, as determined using an unpaired *t*-test. (**C**) KEGG pathway analysis of similar DEGs, showing top 10 enriched pathways. (**D**) Upper: fold change of top 7 upregulated genes associated with “Efferocytosis” and “Pathways in cancer.” Lower: corresponding TPM values. ***P < 0.001 versus DMSO, as determined using an unpaired *t*-test. Full gene lists are provided in Table S4. Genes shown in panels B and D were selected based on the following criteria: |FC| ≥ 2 and TPM ≥ 100. (**E–H**) RT-qPCR validation of selected genes: *canine DHRS2* (**E**), *S100A14* (**F**), *NDRG1* (**G**), and *NQO1* (**H**). Expression was normalized to *canine β-actin* and expressed relative to levels in DMSO-treated and virus-free controls (set to 1). Data are presented as means ± SEM (*n* = 4). Statistical significance was determined via unpaired *t*-tests; ***P < 0.001 for the indicated comparisons. (**I**) NRF2 reporter assay using a dual-luciferase (*Firefly*/*Renilla*) system. MDCK cells were co-transfected with pNQO1-ARE-*Firefly* luciferase and pRL-TK-*Renilla* luciferase plasmids and subsequently treated with Mel56 (10 µM). DMSO (0.25%) or dl-sulforaphane (25 µM) were used as negative or positive controls, respectively. The mRNA expression levels of *Firefly* and *Renilla* luciferase were analyzed by RT-qPCR and normalized to *canine β-actin* mRNA. Relative *Firefly*/*Renilla* luciferase expression was calculated and expressed relative to levels in DMSO-treated cells (set to 1). Data are presented as means ± SEM (*n* = 6) from two independent experiments. *P < 0.05, ***P < 0.001 vs DMSO, as determined using one-way ANOVA followed by Dunnett’s *post hoc* tests.

The upregulated genes were significantly enriched in the “biological regulation” and “regulation of biological process” categories, which ranked among the top 2 BP terms (Fig. 5A). Among these, the top 7 upregulated genes—VGF nerve growth factor inducible (*VGF*), dehydrogenase/reductase 2 (*DHRS2*), prostaglandin-endoperoxide synthase 2 (*PTGS2*), S100 calcium-binding protein A14 (*S100A14*), *N*-myc downstream regulated gene 1 (*NDRG1*), vascular endothelial growth factor A (*VEGFA*), and *DHRS9*—were selected based on |FC| ≥ 2 and TPM ≥100 (*P* < 0.001, Fig. 5B). Likewise, a KEGG pathway analysis identified “Efferocytosis” and “Pathways in cancer” categories as the top 2 enriched pathways (Fig. 5C), in which the top 7 upregulated genes—*PTGS2*, *VEGFA*, integrin subunit alpha 2 (*ITGA2*), solute carrier family 2 member 1 (*SLC2A1*; encoding the glucose transporter 1 [GLUT1]), integrin subunit alpha 3 (*ITGA3*), laminin subunit gamma 2 (*LAMC2*), and NAD(P)H quinone dehydrogenase 1 (*NQO1*)—were selected based on the same criteria (*P* < 0.001, Fig. 5D). Subsequently, an RT-qPCR analysis confirmed that the expression levels of *canine VGF*, *DHRS2*, *S100A14*, *NDRG1*, *DHRS9*, *SLC2A1*, *ITGA3*, and *NQO1* were significantly upregulated in Mel56-treated MDCK cells infected with or without the A/PR/8/34 virus compared with levels in DMSO-treated controls (*P* < 0.001, Fig. S6A through D; Fig. 5E through H). Several of these genes are associated with oxidative stress regulation. NQO1 is transcriptionally activated by NRF2, a key regulator of the antioxidant response triggered by ROS (35). DHRS2 reduces mitochondrial ROS (mtROS) levels (36). S100A14 mitigates oxidative stress by stabilizing glutaminase (37), while *NDRG1* is upregulated in response to oxidative stress along with heme oxygenase-1, another NRF2-inducible antioxidant factor (35, 38). Additionally, SLC2A1/GLUT1 overexpression promotes cell survival under glucose deficiency-induced oxidative stress (39). We therefore evaluated whether Mel56 activates NRF2 in MDCK cells using an NRF2 reporter assay. Mel56 (*P* < 0.05) increased NRF2 activation significantly, similar to the effects of the known NRF2 activator dl-sulforaphane (*P* < 0.001), compared with levels in the DMSO-treated control group (Fig. 5I).

Together, these results suggest that Mel56 attenuates virus-induced host immune responses while enhancing antioxidant gene expression via NRF2 activation.

### Mel56 impairs mitochondrial function and promotes mtROS production

Mitochondria are a major source of cellular ROS, and enhanced mtROS production leads to oxidative stress, which subsequently activates the NRF2-mediated antioxidant response (40). PHBs perform multiple functions within mitochondria, including the maintenance of mitochondrial structural integrity and dynamics (41). *PHB* knockdown impairs the formation of mitochondrial respiratory supercomplexes and increases mtROS generation (42). Mel56 binding to PHBs is expected to produce mtROS by attenuating mitochondrial function through PHB activity inhibition. Thus, to explore the mechanism underlying antioxidant gene upregulation via NRF2 activation, we examined mitochondrial function in isolated rat liver mitochondria and assessed mitochondrial function and mtROS production in live MDCK cells following Mel56 treatment.

**Fig 6:**
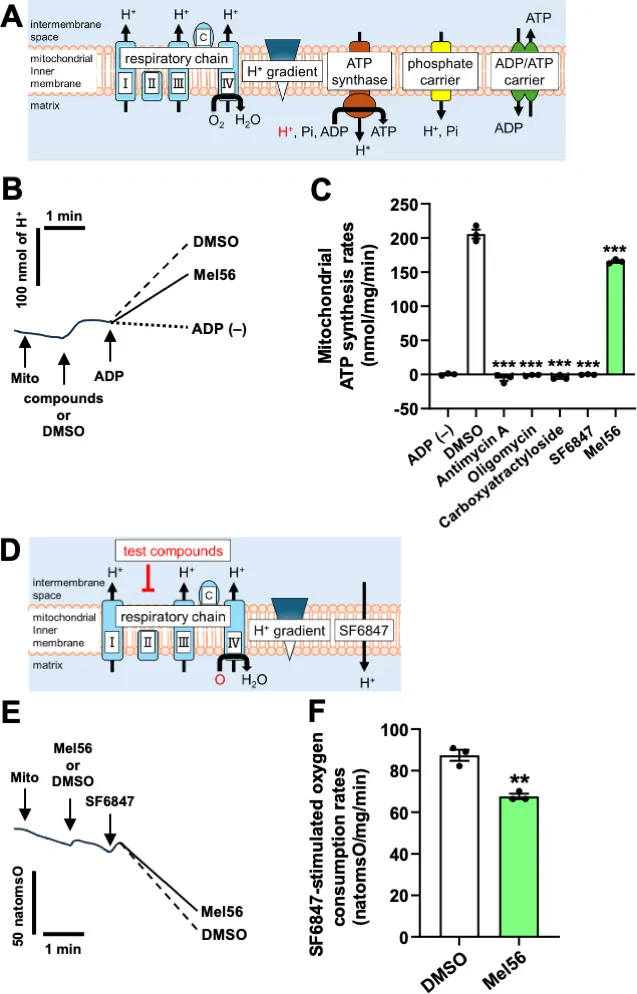
Mel56 inhibits mitochondrial ATP synthesis and respiration in rat liver mitochondria. (**A**) Schematic diagram showing components of the mitochondrial inner membrane involved in ATP synthesis. “C” represents mitochondrial cytochrome c. Since mitochondrial ATP synthesis is an overall reaction, the inhibition of each protein causes a decrease in ATP synthesis. (**B**) Time-course tracing of H^+^ concentration changes in the presence or absence of ADP and mitochondrial respiratory substrates. “Mito” represents the addition of rat liver mitochondria. Mel56 (10 µM), DMSO (0.1%), and known mitochondrial inhibitors (antimycin A [1 µM], oligomycin [1 µg/mL], carboxyatractyloside [1 µM], and SF6847 [100 nM]) were tested. (**C**) Mitochondrial ATP synthesis rates (nmol/mg/min). DMSO control: 206 ± 9 nmol/mg/min. Data are presented as means ± SEM (*n* = 3) from three independent experiments. ***P < 0.001 versus DMSO, as determined using one-way ANOVA followed by Dunnett’s *post hoc* tests. (**D**) Model schematic for compound-mediated inhibition of SF6847-stimulated mitochondrial respiration. (**E**) Time-dependent oxygen consumption in mitochondria treated with DMSO or Mel56. (**F**) SF6847-stimulated oxygen consumption rates (natoms O/mg/min). DMSO control: 87 ± 4 natoms O/mg/min. Data are presented as means ± SEM (*n* = 3) from three independent experiments. **P < 0.01 versus DMSO, as determined by unpaired *t*-tests.

We first assessed whether Mel56 inhibits mitochondrial functions, including ATP synthesis. Since mitochondrial ATP synthesis reflects the integrated activity of various proteins in the inner mitochondrial membrane (Fig. 6A), we examined whether Mel56 inhibits specific components involved in this process, which may result in reduced ATP production in rat liver mitochondria (Fig. 6B and C). As mitochondrial ATP synthesis consumes protons (H^+^) in the reaction mixture, the addition of ADP to a mitochondrial suspension containing a respiratory substrate, inorganic phosphate, and magnesium ions induces a time-dependent alkalinization of the reaction mixture (43). Therefore, the inhibitory effects of these compounds on mitochondrial ATP synthesis were evaluated by measuring the alkalinization rate (43). Mel56 reduced mitochondrial ATP synthesis significantly compared with that in the DMSO (0.1%) control (*P* < 0.001, Fig. 6C), similar to the strong effects of known inhibitors of specific mitochondrial proteins or uncouplers. These included antimycin A (1 µM), an inhibitor of complex III; oligomycin (1 µg/mL), an ATP synthase (complex V) inhibitor; carboxyatractyloside (1 µM), an inhibitor of the ADP/ATP translocase; and SF6847 (tyrphostin A9, 100 nM), a potent epidermal growth factor receptor inhibitor and protonophore that markedly stimulates mitochondrial oxygen consumption (43).

We next evaluated the effects of Mel56 on the mitochondrial electron transport system, which drives ATP synthesis (Fig. 6D). Since oxygen is consumed by the electron transport chain, the addition of SF6847 (100 nM) to a mitochondrial suspension containing a respiratory substrate, inorganic phosphate, and magnesium ions causes a time-dependent decrease in the oxygen concentration (43). Inhibitory effects of the compounds on the electron transport system were assessed by measuring the rate of oxygen consumption (43). Mel56 suppressed oxygen consumption stimulated by the uncoupler SF6847 significantly compared with that for DMSO (0.1%) (Fig. 6E, *P* < 0.01, Fig. 6F), suggesting the inhibition of the mitochondrial electron transport chain.

We further investigated whether Mel56 affects the permeability of the mitochondrial inner membrane, as observed with SF6847 (Fig. S7A). In the presence of an uncoupler, mitochondrial oxygen consumption is accelerated by membrane permeabilization (43). Accordingly, we measured the oxygen consumption rate to evaluate membrane permeabilization (43). While SF6847 increased the rate of oxygen consumption significantly, Mel56 increased inner mitochondrial membrane permeability modestly compared with that for DMSO (0.1%) (Fig. S7B, *P* < 0.001 or *P* = 0.057, Fig. S7C).

**Fig 7:**
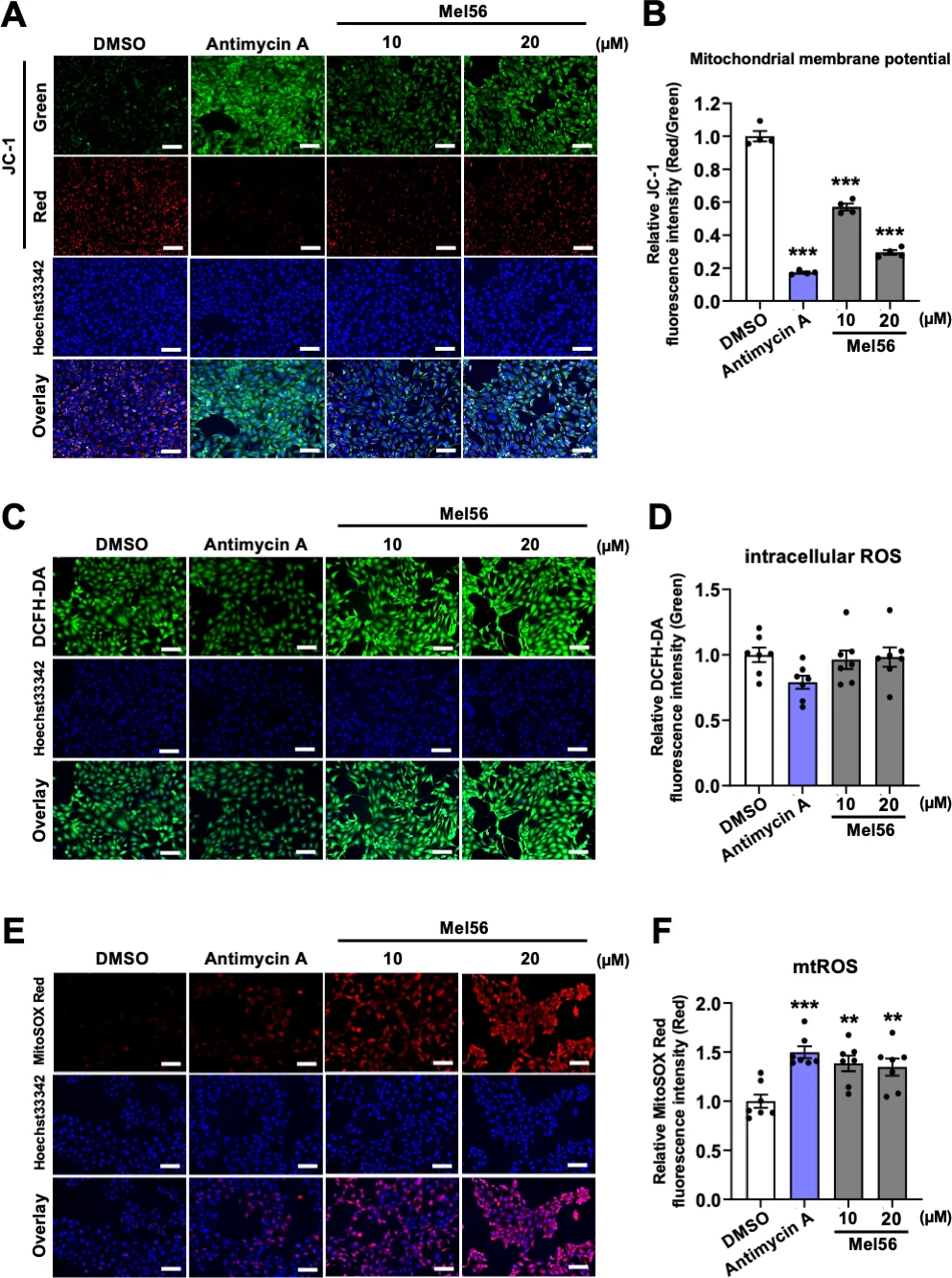
Mel56 attenuates mitochondrial membrane potential and promotes mtROS production in live MDCK cells. (**A and B**) Analysis of mitochondrial membrane potential in live MDCK cells using JC-1 staining. MDCK cells cultured in 35 mm glass-bottom dishes with 14 mm micro-wells or in 96-well optical-bottom black plates (1 × 10^4^ cells/well) were stained with JC-1 (4 µM). For imaging experiments, Hoechst 33342 (10 µM) was added for nuclear staining. Cells were treated with Mel56 (10 µM–20 µM) for 2 h. DMSO (0.2%) or antimycin A (10 µM) were used as negative or positive controls, respectively. (**A**) Live cells in glass-bottom dishes were observed under a fluorescence microscope, and images of JC-1 green, JC-1 red, and Hoechst 33342 (blue, nuclei) fluorescence were captured. Scale bars: 100 µm. (**B**) In 96-well plates, fluorescence intensities of JC-1 green and red were measured using a fluorescence plate reader at excitation/emission wavelengths of 485/535 nm (green) and 535/595 nm (red), respectively. Mitochondrial membrane potential is expressed as the JC-1 red/green fluorescence intensity ratio. Data are presented as means ± SEM (*n* = 4). ***P < 0.001 versus DMSO, as determined using one-way ANOVA followed by Dunnett’s *post hoc* tests. Results were reproducible across experiments. (**C−F**) Analysis of intracellular and mtROS production in live MDCK cells using DCFH-DA and MitoSOX Red staining, respectively. MDCK cells cultured in 35 mm glass-bottom dishes (3 × 10^4^ cells/well) or 96-well optical-bottom black plates (1 × 10^4^ cells/well) were treated with Mel56 (10 µM–20 µM) for 2 h. DMSO (0.2%) or antimycin A (10 µM) were used as negative or positive controls, respectively. (**C and E**) Cells in glass-bottom dishes were stained with DCFH-DA (1:1,000 dilution) or MitoSOX Red (2.5 µM), respectively. Hoechst 33342 (10 µM) was included for nuclear visualization. After 30 min of incubation, fluorescence images of DCFH-DA (green; **C**) or MitoSOX Red (red; **E**), together with Hoechst 33342 (blue, nuclei), were acquired by fluorescence microscopy. Scale bars: 100 µm. (**D and F**) Cells in 96-well plates were stained with DCFH-DA (1:1,000 dilution) or MitoSOX Red (2.5 µM) alone. After 30 min of incubation, fluorescence intensities were measured using a fluorescence plate reader at excitation/emission wavelengths of 490/530 nm (DCFH-DA, green; **D**) and 510/580 nm (MitoSOX Red, red; **F**). Data are presented as means ± SEM (*n* = 6) from two independent experiments. **P < 0.01, ***P < 0.001 versus DMSO, as determined using one-way ANOVA followed by Dunnett’s *post hoc* tests.

We next examined the effects of Mel56 on mitochondrial membrane potential, intracellular ROS, and mtROS production in live MDCK cells using JC-1, DCFH-DA, and MitoSOX Red staining, respectively. JC-1 is a membrane potential-dependent dye that forms red-fluorescent aggregates in polarized mitochondria and remains as green-fluorescent monomers when mitochondrial membrane potential is reduced. DCFH-DA fluoresces upon oxidation by intracellular ROS. MitoSOX Red detects mitochondrial superoxide. Mel56- or antimycin A-treated cells exhibited increased JC-1 green fluorescence and decreased red fluorescence compared with those of DMSO-treated controls (Fig. 7A). The JC-1 red/green fluorescence ratio was significantly reduced (*P* < 0.001, Fig. 7B), indicating attenuated mitochondrial membrane potential. Although Mel56 did not alter total intracellular ROS levels significantly, as assessed by DCFH-DA staining (Fig. 7C and D), MitoSOX Red fluorescence increased markedly in Mel56- or antimycin A-treated cells (*P* < 0.001, Fig. 7E and F), indicating enhanced mtROS production. Collectively, these findings demonstrate that Mel56 induces mitochondrial dysfunction, leading to increased mtROS production and subsequent activation of antioxidant responses.

### Mel56 inhibits SARS-CoV-2 infection independently of NRF2 activation in hiPSC-derived lung organoids

SARS-CoV and SARS-CoV-2 NSPs interact with mitochondrial proteins and are involved in various mitochondrial processes (44). Protein interactome analyses of SARS-CoV-2 and host proteins have demonstrated that PHBs interact with NSP2 and NSP6 of SARS-CoV-2 (27–29). Moreover, pharmacological NRF2 inducers inhibit both SARS-CoV-2 replication and the associated inflammatory response (45). Based on these results, we assessed the potential anti-SARS-CoV-2 activity of Mel56 in hiPSC-derived lung organoids (46).

**Fig 8:**
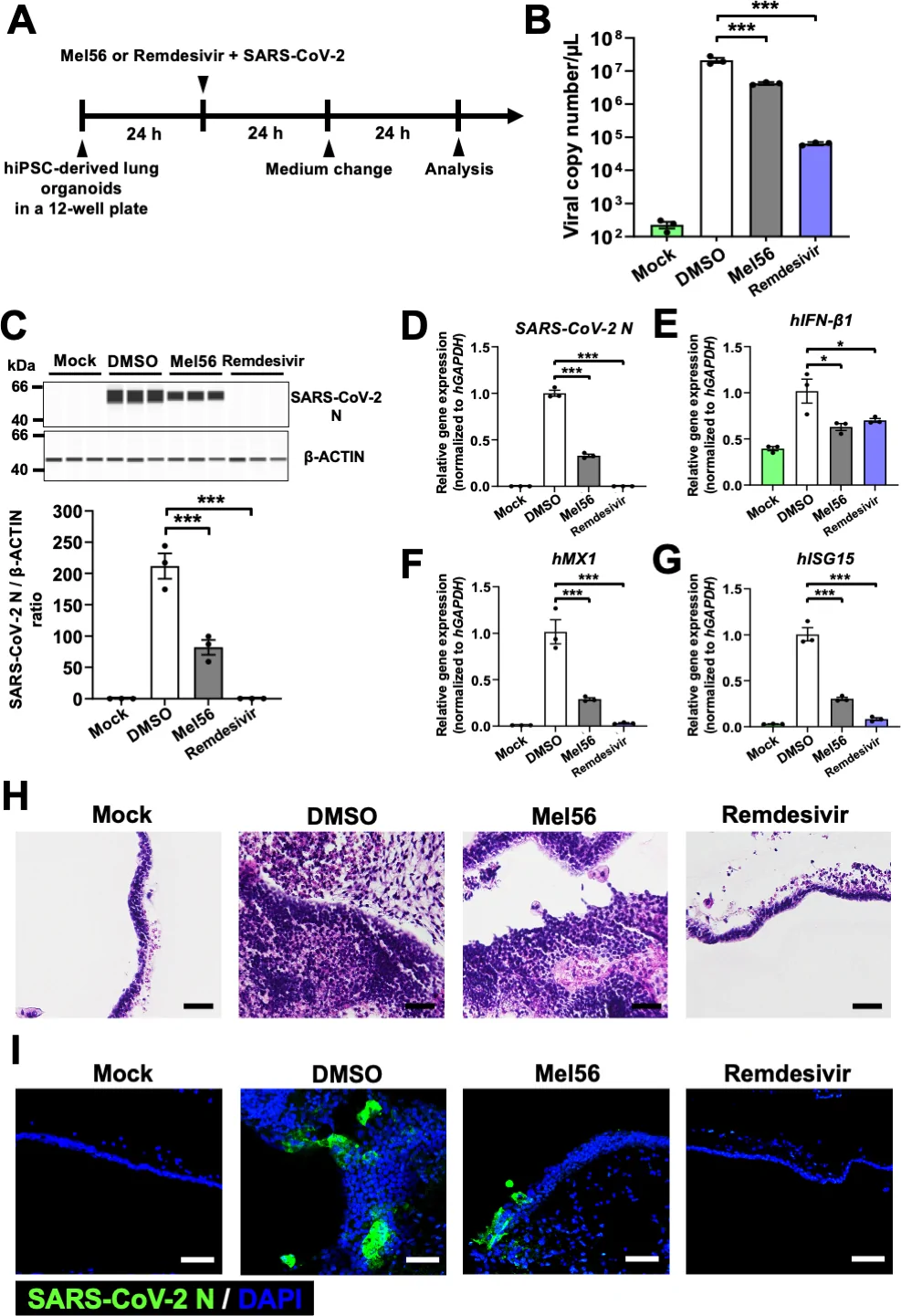
Mel56 suppresses SARS-CoV-2 infection in hiPSC-derived lung organoids. (**A**) Schematic workflow for the infection assay using hiPSC-derived lung organoids infected with SARS-CoV-2 Omicron XDQ.1. (**B**) Viral copy number in the culture supernatant measured using RT-qPCR following treatment with DMSO (0.1%), Mel56 (10 µM), or remdesivir (10 µM). Data are shown as means ± SEM (*n* = 3) from two independent experiments. ***P < 0.001 versus DMSO, as determined using one-way ANOVA followed by Dunnett’s *post hoc* tests. (**C**) SARS-CoV-2 N protein levels in virus-infected lung organoids analyzed by capillary-based immunoassay (Jess system). β-Actin was used for normalization. “Mock” denotes uninfected and untreated controls. Data are shown as means ± SEM (*n* = 3) from two independent experiments. ***P < 0.001 versus DMSO, as determined using one-way ANOVA followed by Dunnett’s *post hoc* tests. (**D–F**) mRNA expression levels of SARS-CoV-2 N (**D**) and antiviral host genes *hIFN-β1* (**E**), *hMX1* (**F**), and *hISG15* (**G**) in virus-infected lung organoids, analyzed by RT-qPCR. *hGAPDH* was used for normalization. “Mock” denotes uninfected and untreated controls. Data are presented as means ± SEM (*n* = 3) from two independent experiments. *P < 0.05, ***P < 0.001 versus DMSO, as determined using one-way ANOVA followed by Dunnett’s *post hoc* tests. (**H**) Representative hematoxylin and eosin-stained images of lung organoid sections from each treatment group. (**I**) IF images showing SARS-CoV-2 N protein (green) and DAPI-stained nuclei (blue); merged images shown. Scale bars: 50 µm. Data represent two independent experiments with consistent results.

As shown in Fig. S8A and Fig. 8A, hiPSC-derived lung organoids cultured in 96- or 12-well plates were treated with Mel56 (0.31 µM–10 µM), followed by infection with or without SARS-CoV-2 Omicron XDQ.1. DMSO (0.1% or 0.2%) and remdesivir (10 µM) were used as negative and positive controls, respectively. Lung organoids were analyzed at 48 and 72 hpi. In the viral assay using a 96-well plate, Mel56 (5 µM–10 µM) reduced viral copy numbers in the culture supernatant of SARS-CoV-2-infected hiPSC-derived lung organoids in a dose-dependent manner compared with copy numbers in the DMSO (0.2%) treatment (*P* < 0.05 at 5 µM and *P* < 0.001 at 10 µM, Fig. S8B). However, treatment with 10 µM Mel56 reduced lung organoid viability slightly (*P* < 0.01, Fig. S8C). We further evaluated the SI of Mel56 against SARS-CoV-2 infection in hiPSC-derived lung organoids, as shown in Table 2, using the same calculation method described in Table 1. The SI values were calculated based on CC_50_ values determined by the WST-8 assay in hiPSC-derived lung organoids (Fig. S8C) and IC_50_ values derived from a qPCR analysis of viral RNA levels in SARS-CoV-2-infected organoids cultured in 96-well plates (Fig. S8B). The calculated SI value was approximately 2.0 (Table 2).

**TABLE 2:** The SI value of Mel56 against anti-SARS-CoV-2 activity^*a*^

Cell	Experiment	SARS-CoV-2 strain	IC_50_ (µM)	CC_50_ (µM)	SI value (CC_50_/IC_50_)
hiPSC-derived lung organoid	qPCR analysis (viral copy number)	SARS-CoV-2 Omicron XDQ.1	5.8	11.5	2.0

^*a*^ The SI was calculated using the same method described in Table 1. CC_50_ values of Mel56 were determined by the WST-8 cell viability assay using hiPSC-derived lung organoids, as described above (Fig. S8C). IC_50_ values were determined based on qPCR analyses of viral RNA levels (Fig. S8B). Dose–response curves were generated, and all CC_50_ and IC_50_ values were calculated. An SI value greater than 1.0 was considered indicative of greater antiviral efficacy than cytotoxicity in the respective cell model.

Similarly, in the viral assay using a 12-well plate, both Mel56 and remdesivir (10 µM each) significantly reduced viral copy numbers in the culture supernatant (Fig. 8B) and decreased SARS-CoV-2 nucleocapsid (N) protein expression (Fig. 8C) in infected lung organoids compared with those in the DMSO (0.1%) treatment (*P* < 0.001, Fig. 8B and C). RT-qPCR analyses further showed that Mel56 and remdesivir significantly decreased the expression levels of the *SARS-CoV-2 N* gene (*P* < 0.001, Fig. 8D) as well as human antiviral genes, including *hIFNβ1* (*P* < 0.05, Fig. 8E), *hMX1* (*P* < 0.001, Fig. 8F), *hISG15* (*P* < 0.001, Fig. 8G), and *hISG56* (*P* < 0.001, Fig. S9A), in infected organoids compared with levels in the DMSO group (Fig. 8D through G; Fig. S9A). Hematoxylin and eosin staining (Fig. 8H) and IF analyses (Fig. 8I) indicated that Mel56 treatment suppresses the morphological changes in lung organoids induced by SARS-CoV-2 infection. Consistently, there were slightly fewer N protein-positive cells in infected lung organoid sections treated with Mel56 than with DMSO (Fig. 8I). Importantly, under conditions in which Mel56 exhibited anti-SARS-CoV-2 activity, the expression levels of NRF2 target genes, including *hNQO1* and *hHMOX1*, were comparable to those in DMSO-treated infected organoids (Fig. S9B and C), indicating that NRF2 signaling was not activated in this context.

Collectively, these results demonstrate that Mel56 exerts antiviral activity against SARS-CoV-2 independently of NRF2 activation in a physiologically relevant lung organoid model.

## DISCUSSION

In this study, we demonstrated that Mel56, but not Mel6, effectively inhibits infection by influenza A H1N1 and H3N2 viruses in MDCK and A549 cells. In line with results of extensive structure-activity relationship studies of triazines targeting PHBs (33), a comparison of the chemical structures of Mel6 and Mel56 (see Fig. S1A) suggested that the position of the protonable tertiary amine and the triazine skeleton are both important for anti-influenza activity. The combination of PHB2 knockdown and Mel56 treatment further reduced viral NP expression, indicating an additive effect and implicating PHBs in the antiviral Mel56 mechanism. Transcriptomic analysis revealed that Mel56 downregulates virus-induced antiviral immune response genes and upregulates genes associated with the antioxidant response. NRF2 activation by Mel56 was confirmed using a reporter assay, supporting the involvement of NRF2 signaling. Furthermore, Mel56 impaired mitochondrial ATP synthesis by disrupting the electron transport chain in isolated rat liver mitochondria. In live cells, Mel56 reduced mitochondrial membrane potential and promoted mtROS production. These mitochondrial modulations likely contribute to NRF2 activation. In addition to its anti-influenza activity, Mel56 exerts antiviral effects against SARS-CoV-2 independently of NRF2 activation in a physiologically relevant lung organoid model. Overall, these findings suggest that Mel56 functions as a broad-spectrum host-directed antiviral agent targeting PHBs, supporting its potential as a lead compound for the development of therapeutics against multiple respiratory viruses.

Mel56 is a synthetic triazine derivative that binds to PHB1 and PHB2 and has previously been shown to promote melanogenesis and induce apoptosis in melanoma cells (33, 34). PHBs are multifunctional scaffolding proteins implicated in the replication of several viruses, including IAV (22–25), SARS-CoV (26, 27), and SARS-CoV-2 (28, 29). In IAV-infected cells, PHB is upregulated, and siRNA-mediated knockdown of PHBs reduces viral replication (22, 24, 25), supporting a proviral role under basal conditions. In the present study, both *PHB2* DsiRNA and Mel56 independently reduced viral NP expression, and their combination exerted a stronger suppressive effect, indicating that PHB function is mechanistically linked to viral replication and can be disrupted by Mel56. Given that PHBs are essential for maintaining mitochondrial integrity and function, their reduction by siRNA likely induces mitochondrial stress and increases ROS production, as previously reported (47). Such mitochondrial stress may activate the NRF2 pathway, which exerts antiviral effects and may therefore explain the reduced NP expression observed following *PHB2* knockdown. Similarly, Mel56 binding to PHBs modulated mitochondrial function, resulting in enhanced mtROS production and NRF2 activation (Fig. 5 to 7). Mel56 also induces cell apoptosis via the loss of mitochondrial potential and caspase activation in melanoma lines (48). Thus, although intact PHB complexes may facilitate efficient viral replication, their functional perturbation—either through genetic knockdown or pharmacological targeting by Mel56—appears to shift mitochondrial homeostasis toward a stress-responsive state that activates NRF2 signaling and suppresses IAV replication. This interpretation reconciles the apparent paradox between the basal proviral role of PHBs and the antiviral effects observed upon their modulation. Rather than functioning as direct antiviral factors, PHBs may serve as regulatory nodes whose disruption triggers host stress responses that limit viral replication. This model aligns with the concept of host-directed antiviral strategies, in which controlled modulation of host factors activates protective signaling pathways rather than directly targeting viral components. In this context, Mel56 binding to PHBs may alter PHB-dependent mitochondrial signaling to promote NRF2-mediated antiviral responses.

Recent interactome analyses have revealed extensive interactions between PHBs and SARS-CoV-2 proteins. PHBs bind to NSPs, including NSP2 and NSP6 (27–29). In a comprehensive protein interaction study, PHBs have been found to interact with multiple viral components, including structural proteins (S, M, and E), 14 NSPs (NSP1, NSP2, and NSP4–15), and several accessory proteins (49). Among these, NSP2 interacts with ribosomes and replication-transcription complexes and plays a role in viral transcription and translation. NSP2 is involved in host translation suppression, miRNA-mediated transcript silencing, and mitochondrial Ca²^+^ homeostasis (27, 50, 51). NSP6 plays a critical role in the formation of viral replication organelles via membrane rearrangement (52). Given these interactions, Mel56 may inhibit SARS-CoV-2 replication by interfering with the PHB-mediated support of NSP2 and NSP6 functions. Further studies are required to determine the precise mechanisms by which Mel56–PHB-binding affects viral protein function and replication dynamics.

Our transcriptomic analysis indicated that Mel56 upregulates several antioxidant response-related genes, including *NQO1*, *DHRS2*, *S100A14*, *NDRG1*, and *SLC2A1*. NRF2 activation by Mel56 was confirmed using a reporter assay. Mel56 also impaired mitochondrial ATP synthesis by suppressing electron transport in isolated mitochondria. In live cells, Mel56 decreased mitochondrial membrane potential and enhanced mtROS production. Collectively, these mitochondrial alterations suggest that mtROS generation may contribute to the activation of NRF2 signaling. We have previously reported that baku, a natural PHB2-binding compound with anti-influenza activity, also activates NRF2 signaling and induces the expression of antioxidant genes, including *NQO1* and *glutathione S-transferase A3* (24, 30). Baku impairs mitochondrial function and reduces lipid peroxidation via mtROS generation (53, 54). These results suggest that Mel56, similar to baku, exerts its antiviral effects via the mtROS-dependent activation of the NRF2 pathway. NRF2 activation has been linked to the suppression of SARS-CoV-2 replication and associated inflammation (45, 55). Pharmacological NRF2 activators, such as 4-octyl-itaconate, dimethyl fumarate, sulforaphane, and bardoxolone, inhibit viral replication and inflammation. SARS-CoV-2 NSP14 suppresses NRF2 activation via Sirtuin 1 inhibition (55), and NRF2 activity is diminished in the lungs of patients with COVID-19 (45). Moreover, NRF2 activation reduces *ACE2* mRNA levels, a key receptor for SARS-CoV-2 entry, suggesting an additional antiviral mechanism (56). However, under conditions in which Mel56 exerted anti-SARS-CoV-2 activity, it did not upregulate the expression of NRF2 target genes, including *NQO1* and *HMOX1*, in virus-infected lung organoids, indicating that NRF2 signaling was not activated in this setting. These results suggest that NRF2 activation is cell type- or virus-dependent. Accordingly, the contribution of NRF2 signaling to antiviral activity may differ between the IAV and SARS-CoV-2 models. Overall, these observations highlight the context-dependent nature of the antiviral effects of Mel56 and underscore the complexity of host signaling pathways during viral infection.

Despite these similarities, Mel56 and baku exhibit strain-specific differences in antiviral potency; Mel56 is less effective than baku against influenza A H1N1 but more effective against H3N2. If their effects were limited to general mitochondrial or antioxidant responses, such strain-dependent differences would not be expected. One possible explanation is that these compounds differentially disrupt the interaction between prohibitins and influenza viral components. For instance, baku may more efficiently destabilize PHB-virus interactions in H1N1, whereas Mel56 may be more effective in H3N2. Further studies are needed to clarify whether the antiviral selectivity of Mel56 and baku involves differential modulation of PHB-mediated virus-host interactions.

Both Mel6 and Mel56 activated MITF signaling in MDCK cells; however, only Mel56 reduced influenza NP expression, indicating that MITF activation was not essential for the anti-influenza activity of Mel56. Previous studies have linked MITF dysregulation to COVID-19-related pathogenesis and pregnancy outcomes (57, 58), and MITF has been identified as an upregulated gene in SARS-CoV-2 infection (59). These observations suggest that MITF activation influences host responses to SARS-CoV-2, although its role in antiviral defense remains unclear. The PHB–LC3–MITF signaling axis may contribute to the anti-SARS-CoV-2 effects of Mel56 and related compounds.

A range of PHB ligands have been identified, among which flavaglines—cyclopenta[*b*]benzofuran derivatives from *Aglaia* species—are particularly notable for their antiviral and cytoprotective activities (34). Initially considered as eukaryotic initiation factor 4F (eIF4F) inhibitors, flavaglines, such as rocaglamide, were recognized as PHB1 ligands that disrupt PHB1-eIF4F interactions (60, 61). eIF4F is essential for the translation of several RNA viruses, including the Ebola virus, Zika virus, chikungunya virus, influenza virus, SARS-CoV, and SARS-CoV-2 (60, 62). Both natural and synthetic flavaglines—including zotatifin, CR-31-B, FL3, and others—have demonstrated antiviral activity. For example, silvestrol inhibits eIF4A to suppress the replication of coronaviruses, picornaviruses, and influenza viruses and selectively inhibits SARS-CoV-2 mRNA translation (49). These findings suggest that PHB-targeting flavonoids and triazines, such as Mel56, are a promising class of broad-spectrum antiviral agents that target host translational and mitochondrial pathways.

This study had several limitations. First, all mechanistic analyses were conducted using *in vitro* systems, including MDCK and A549 cells and hiPSC-derived lung organoids. Although these models provide important mechanistic insights, *in vivo* studies using appropriate animal models will be required to evaluate the pharmacokinetics, bioavailability, toxicity, and therapeutic efficacy of Mel56 against respiratory viral infections.

Second, the reduction in the proportion of NP-positive cells without a substantial decrease in NP signal intensity among infected cells (shown in Fig. 2) suggests that Mel56 primarily affects an early stage of the viral life cycle. This pattern is consistent with a potential impact on viral entry, uncoating, or early replication events rather than direct inhibition of viral protein synthesis in already infected cells. However, because time-of-addition and viral entry assays were not performed in this study, the precise step targeted by Mel56 remains undefined. Future experiments designed to dissect the temporal stage of viral inhibition are necessary to clarify this mechanism.

Third, although previous studies have reported that melanogenin derivatives, including Mel56, directly bind to PHB1 and PHB2 in pull-down experiments using a derivative conjugated to agarose (33, 34), the present study did not include additional biophysical or structural analyses to confirm direct target engagement under antiviral conditions. Moreover, although Mel56 reduced viral NP expression and suppressed infection in MDCK and A549 cells and was associated with mitochondrial modulation, increased mtROS production, and NRF2 activation, the necessity of these pathways has not been formally demonstrated using genetic or pharmacological inhibition approaches. In addition, the additive effect observed with PHB2 knockdown does not establish whether Mel56 and PHB perturbation act through identical or partially overlapping mechanisms. Thus, while our findings support a functional association between PHB modulation, mitochondrial stress responses, and antiviral activity, further biochemical and genetic studies are required to definitively identify the direct molecular target(s) and clarify the mechanistic basis of the antiviral effects of Mel56.

Finally, this study focused on only two triazine derivatives, Mel6 and Mel56. A broader structure–activity relationship analysis encompassing additional PHB-targeting compounds is necessary to optimize antiviral potency and selectivity. Addressing these limitations in future studies is essential to validate Mel56 as a host-directed broad-spectrum antiviral agent.

### Conclusion

**Fig 9:**
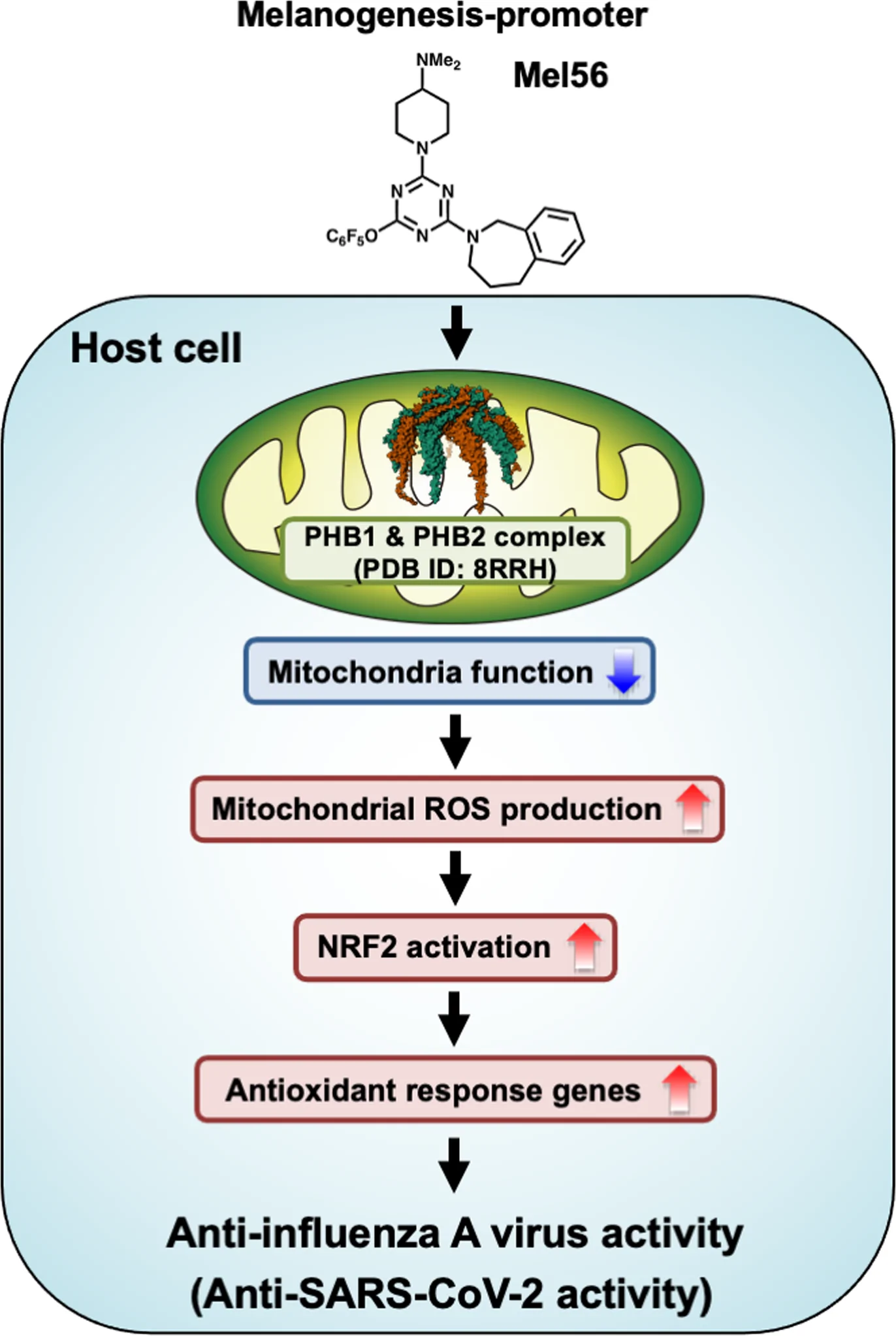
Schematic showing the mechanisms underlying the anti-IAV and anti-SARS-CoV-2 activities of Mel56 by targeting PHBs. Information on the tertiary structures of PHB1 and 2 complex (PDB ID: 8RRH) was obtained from the RCSB Protein Data Bank (https://www.rcsb.org) and visualized using Mol* viewer (WebGL). PHB complexes are bell-shaped scaffolds mainly localized in the inner membrane (63). These findings provide a new strategy for the development of antiviral drugs.

Figure 9 summarizes the principal findings of this study. We demonstrated that Mel56 exhibits antiviral activity against both IAV and SARS-CoV-2 in conventional cell culture models as well as in physiologically relevant human lung organoids. Mechanistically, Mel56 upregulates antioxidant response-related genes, activates NRF2 signaling, impairs mitochondrial function, and enhances mtROS production. Notably, its effects vary depending on the cellular context, underscoring the complexity of host signaling pathways during viral infection. These effects are likely mediated, at least in part, through binding to PHBs and modulation of host mitochondrial function, leading to NRF2 activation. These findings provide new insights into the potential of PHB-targeting compounds as antiviral agents and highlight a mechanistic link between melanogenin derivatives, PHBs, mitochondrial regulation, and antiviral responses. Collectively, our results support the development of PHB-targeting ligands as a promising strategy for broad-spectrum antiviral therapy.

## MATERIALS AND METHODS

### Cell lines

MDCK cells were provided by Dr. Masahiro Nagahama (Tokushima Bunri University). A549 cells were purchased from Japanese Collection of Research Bioresources (JCRB) Cell Bank (JCRB0076, Osaka, Japan). These cells were cultured in growth medium containing 10% FBS (high-glucose Dulbecco’s modified Eagle’s medium [DMEM; Nacalai Tesque, Kyoto, Japan] supplemented with 10% FBS [Thermo Fisher Scientific, Waltham, MA, USA], 100 U/mL penicillin, 100 μg/mL streptomycin [P/S; Thermo Fisher Scientific], and 4 mM l-glutamine). TMPRSS2/Vero cells were purchased from JCRB Cell Bank (JCRB1818) and cultured in growth medium containing 5% FBS (Eagle’s minimum essential medium [FUJIFILM Wako Pure Chemical] supplemented with 5% FBS [Thermo Fisher Scientific] and P/S [Thermo Fisher Scientific]). Cells were maintained at 37°C in a humidified atmosphere containing 5% CO_2_.

hiPSCs (1383D6 line) were provided by Dr. Masato Nakagawa (Kyoto University) and maintained on iMatrix-511 (Nippi, Tokyo, Japan)-coated plates in StemFit AK02N medium (Ajinomoto Healthy Supply, Tokyo, Japan). Cells were passaged every 6 days using TrypLE Select Enzyme (Thermo Fisher Scientific) and plated in the presence of 10 µM Y-27632 (FUJIFILM Wako Pure Chemical, Osaka, Japan).

### Compound preparation

Mel6 and Mel56 (Fig. S1A) were synthesized according to an established procedure (33). Baku was synthesized using previously reported methods (64). dl-Sulforaphane was purchased from Sigma-Aldrich (St. Louis, MO, USA; S4441). Remdesivir (GS-5734) was purchased from MedChemExpress (Monmouth Junction, NJ, USA; HY-104077). Stock solutions (10 mM) were prepared by dissolving these compounds in DMSO.

### Analysis of MDCK cell viability following compound treatment using MTT and WST-8 assays

MDCK (1 × 10^4^ cells/well) or A549 (2 × 10^4^ cells/well) cells were seeded in a 96-well plate and treated with Mel6 or Mel56 in DMSO at concentrations of 40 μM (0.4%), 20 μM (0.2%), 10 μM (0.1%), 5 μM (0.05%), 2.5 μM (0.025%), or 1.25 μM (0.0125%) in either growth medium containing 10% FBS or serum-free medium (high-glucose DMEM [Nacalai Tesque] supplemented with 1% BSA [FUJIFILM Wako Pure Chemical], P/S [Thermo Fisher Scientific], and 4 mM l-glutamine). The cells were then incubated for 72 h at 37°C in the presence of 5% CO_2_. DMSO (0.0125%–0.4%) was used as the negative control. After incubation, cell viability was assessed using MTT or WST-8 assays with an MTT Cell Counting Kit (Nacalai Tesque) or Cell Count Reagent SF (Nacalai Tesque), respectively, according to the manufacturer’s instructions.

### IAV strains

The IAV strains A/PR/8/34 (H1N1), A/WSN/33 (H1N1), and A/Aichi/2/68 (H3N2) were provided by Takahashi and Kido (65). These viruses were propagated in MDCK cells using serum-free medium supplemented with 3 µg/mL l-tosylamido-2-phenyl ethyl chloromethyl ketone (TPCK)-treated trypsin (Sigma-Aldrich). The culture media were clarified by centrifugation (5,000 rpm, 10 min) and stored at −80°C. Viral titers in these media were determined by immunostaining for influenza A viral NP, as previously described (30, 66, 67). The multiplicity of infection (MOI) used for the IAV infection experiments was determined based on preliminary studies to ensure robust and reproducible viral replication while minimizing excessive cytopathic effects under the experimental conditions. All IAV infection experiments described below were performed in growth medium containing 10% FBS without exogenous trypsin (e.g., TPCK-treated trypsin), as previous reports using similar culture conditions demonstrated efficient proteolytic activation of influenza viruses by endogenous proteases (68–70). With regard to TPCK-treated trypsin, supplementation with this exogenous protease is primarily required for efficient multi-cycle viral propagation. In contrast, our experimental design was intended to evaluate host-directed antiviral effects under conditions compatible with single-cycle or limited-cycle infection, rather than classical multi-cycle amplification assays.

### Analysis of IAV-infected MDCK cell viability by NB staining

MDCK cells were seeded in a 96-well plate (1 × 10^4^ cells/well). Mel6 or Mel56 (0.31 μM–40 μM) was mixed with or without A/PR/8/34 (MOI 0.5), A/WSN/33 (MOI 0.3), or A/Aichi/2/68 (MOI 10) in growth medium containing 10% FBS and incubated for 30 min at 37°C with 5% CO_2_. DMSO (0.0031%–0.4%) and baku (0.8–25 μM) (30) were used as negative and positive controls, respectively. The resulting mixture was added to the cells and incubated for an additional 30 min at 37°C with 5% CO_2_, followed by a 72 h incubation under the same conditions. After incubation, the cells were stained with NB, as described previously (24, 30, 67, 71). Viable cells were stained blue, whereas dead cells remained unstained.

### Western blot analysis of influenza A viral NP or protein expression involved in the activation of MITF signaling in virus-infected cells

MDCK (1 × 10^4^ cells/well) or A549 (2 × 10^4^ cells/well) cells were seeded in a 96-well plate. Mel6 or Mel56 (5–40 or 2.5 μM–20 μM) was mixed with A/PR/8/34 (MOI 0.01), A/WSN/33 (MOI 0.01), or A/Aichi/2/68 (MOI 0.1) in growth medium containing 10% FBS and incubated for 30 min at 37°C with 5% CO_2_. DMSO (0.4 or 0.2%) and baku (10 μM) were used as negative and positive controls, respectively. The resulting mixture was added to the cells. After a 24 h incubation at 37°C with 5% CO_2_, the treated cells were washed with phosphate-buffered saline (PBS)(–) and lysed in 1× SDS sample buffer (62.5 mM Tris-HCl [pH 6.8], 2% SDS, 10% glycerol, 0.005% bromophenol blue, and 5% β-mercaptoethanol).

MDCK cells were seeded in a 24-well plate (1 × 10^5^ cells/well). DMSO (0.1%), baku, Mel6, or Mel56 (each 10 μM) were mixed either without the virus (–) or with A/PR/8/34 virus (MOI 0.01) in the growth medium and incubated for 30 min at 37°C with 5% CO_2_. The resulting mixture was added to the cells. After a 24 h incubation at 37°C with 5% CO_2_, the treated cells were washed with PBS(–) and lysed in 1 × SDS sample buffer.

Lysates were boiled for 5 min and subjected to sodium dodecyl sulfate-polyacrylamide gel electrophoresis. Proteins were then transferred onto polyvinylidene fluoride membranes (Millipore, Billerica, MA, USA) and were detected using primary and secondary antibodies, as listed in Table S1. Signals were visualized using Immobilon Western Chemiluminescent Horseradish Peroxidase Substrate (Millipore). β-Actin expression was used as an internal control. Signal intensities were measured using ImageJ software, and protein expression levels were normalized to those of β-actin.

### Detection of IAV-infected MDCK cells by IF staining

MDCK cells were seeded in a 96-well plate (1 × 10^4^ cells/well). Mel6 or Mel56 (2.5 μM–10 μM each) was mixed with A/PR/8/34 (MOI 0.01), A/WSN/33 (MOI 0.01), or A/Aichi/2/68 (MOI 0.1) viruses in growth medium containing 10% FBS and incubated for 30 min at 37°C with 5% CO_2_. DMSO (0.1%) and baku (10 μM) were used as negative and positive controls, respectively. The resulting mixture was added to the cells. After a 24 h incubation at 37°C with 5% CO_2_, the treated cells were washed with PBS(–) and fixed with 4% paraformaldehyde (FUJIFILM Wako Pure Chemical) for 30 min at 4°C. The cells were then permeabilized with 0.3% Triton X-100 (Nacalai Tesque) for 20 min at 25°C. Influenza A viral NP was detected using primary and secondary antibodies, as described in Table S1. Cell nuclei were stained with DAPI (Thermo Fisher Scientific). Images were captured using a fluorescence microscope (BIOREVO BZ-X700; Keyence, Osaka, Japan). Influenza NP-positive and DAPI-positive cells in each image were counted, and the percentage of influenza NP-positive cells relative to DAPI-positive cells was calculated.

### RT-qPCR analysis of IAV and host gene expression in virus-infected MDCK cells

MDCK cells were seeded in a 96-well plate (1 × 10⁴ cells/well). Mel6 or Mel56 (10 μM each) was mixed with or without A/PR/8/34 (MOI 0.01) in growth medium containing 10% FBS and incubated for 30 min at 37°C with 5% CO₂. DMSO (0.1%) and baku (10 μM) were used as negative and positive controls, respectively. The resulting mixture was added to the cells. After a 24 h incubation at 37°C with 5% CO_2_, the treated cells were washed with PBS(–), and total RNA was extracted from cell lysates using an RNeasy Mini Kit (Qiagen, GmbH, Hilden, Germany). The extracted RNA was used to synthesize cDNA using SuperScript VILO (Thermo Fisher Scientific) according to the manufacturer’s instructions. The synthesized cDNA was used as a template for qPCR, which was performed using the SYBR Green Real-Time PCR Master Mix (TOYOBO, Osaka, Japan) or THUNDERBIRD Next SYBR qPCR Mix (TOYOBO). The primers used are listed in Table S2. PCR and data analyses were conducted using an Applied Biosystems QuantStudio 3 Real-Time PCR System (Thermo Fisher Scientific). Relative gene expression was calculated using the ΔΔCT method. The expression levels of viral genes were normalized to those of 18S ribosomal RNA (rRNA) (31), while the expression levels of host genes were normalized to those of *canine β-actin*.

### Effect of Mel56 on influenza A viral NP expression levels in PHB2 DsiRNA-treated MDCK cells

MDCK cells (1 × 10^5^ cells/well) were transfected with 10 nM human *PHB2* DsiRNA (Design ID: hs.Ri.PHB2.13.2; Integrated DNA Technologies [IDT], San Diego, CA, USA) in a 24-well plate using Lipofectamine RNAiMAX reagent (Thermo Fisher Scientific) via reverse transfection following the manufacturer’s instructions. The cells were then incubated at 37°C with 5% CO_2_ for 48 h. Additionally, 10 nM *NC* DsiRNA (#51-01-14-03; IDT) was used as a negative control. The *PHB2* DsiRNA-transfected cells were incubated with 10 µM Mel56 and 0.01 MOI of A/PR/8/34 virus in growth medium containing 10% FBS at 37°C with 5% CO_2_ for 24 h, while 0.1% DMSO treatment was used as the negative control. WB was performed as described previously, and PHB1, PHB2, and influenza NP expression levels were analyzed.

### RNA-seq analysis

MDCK cells were seeded in a 96-well plate (1 × 10^4^ cells/well). Mel56 (10 μM) was mixed with or without A/PR/8/34 (MOI 0.01) virus in growth medium containing 10% FBS and incubated for 30 min at 37°C with 5% CO_2_. DMSO (0.1%) was used as the negative control. The resulting mixture was added to the cells. After a 24 h incubation at 37°C with 5% CO_2_, total RNA was extracted from cell lysates using an RNeasy Mini Kit (Qiagen).

Total RNA concentrations were calculated using Quant-IT RiboGreen (Thermo Fisher Scientific). To assess the integrity of total RNA, samples were run on a TapeStation RNA ScreenTape (Agilent Technologies, Santa Clara, CA, USA). Only high-quality RNA preparations (RIN greater than 7.0) were used for RNA library construction. A library was independently prepared with 1 µg of total RNA for each sample using the Illumina TruSeq Stranded mRNA Sample Prep Kit (Illumina, San Diego, CA, USA). The first step in the workflow involved purifying the poly-A-containing mRNA molecules using poly-T-attached magnetic beads. Following purification, mRNA was fragmented into small pieces using divalent cations at elevated temperatures. Cleaved RNA fragments were copied into first-strand cDNA using SuperScript II Reverse Transcriptase (Thermo Fisher Scientific) and random primers. This was followed by the synthesis of second-strand cDNA using DNA polymerase I, RNase H, and dUTP. These cDNA fragments underwent an end-repair process, followed by the addition of a single “A“ base and ligation of the adapters. The products were purified and enriched using PCR to create a final cDNA library. Libraries were quantified using KAPA Library Quantification kits for Illumina Sequencing platforms according to the qPCR Quantification Protocol Guide (KAPA Biosystems, Wilmington, MA, USA) and qualified using a TapeStation D1000 ScreenTape (Agilent Technologies). Indexed libraries were then submitted to Illumina NovaSeq X (Illumina), and paired-end (2 × 150 bp) sequencing was performed by Macrogen, Inc.

### RNA-seq data processing and analysis

Paired-end sequencing reads were generated using an Illumina NovaSeq X sequencing platform. Trimmomatic v.0.38 was used to remove adapter sequences and trim poor-quality bases. The cleaned reads were aligned to the *Canis lupus familiaris* (CanFam3.1) genome using HISAT v.2.1.0 (72). Reference genome sequences and gene annotation data were downloaded from the NCBI Genome Assembly and RefSeq databases, respectively. The aligned data (in SAM file format) were sorted and indexed using SAMtools v.1.9. After alignment, the transcripts were assembled and quantified using StringTie v.2.1.3b (73, 74). Gene-level and transcript-level quantifications were performed as raw read counts, fragments per kilobase of transcript per million mapped reads, and TPM.

### Differential gene expression analysis of RNA-seq data

Differential gene expression was analyzed using DESeq2 v.1.38.3 (75) with raw counts as input. In the QC step, genes with non-zero counts in all samples were selected. Principal component analysis and multidimensional scaling plots were generated to confirm similarity in expression between samples. The filtered data set was subjected to RLE normalization to correct for variations in library sizes among samples. The statistical significance of DEGs was determined using the DESeq2 nbinom Wald test (75). FC and *P*-values were extracted from Wald test results. All *P*-values were adjusted using the Benjamini-Hochberg algorithm to control the false discovery rate. |FC| ≥ 2 and adjusted *P*-value <0.05 were the thresholds for significance. Hierarchical clustering of the log-transformed values for significant genes was performed using distance metric = Euclidean distance and linkage method = complete. Gene enrichment and functional annotation analyses for significant genes were performed using g:Profiler (76) (https://biit.cs.ut.ee/gprofiler/orth) against the GO database and an in-house KEGG viewer script against the KEGG pathway database (https://www.genome.jp/kegg/pathway.html). Adjusted *P*-values obtained using g:Profiler were derived using a one-sided hypergeometric test and corrected using the Benjamini–Hochberg method. Adjusted *P*-values from the KEGG viewer results were derived using a two-sided modified Fisher’s exact test and corrected using the Benjamini-Hochberg method. All data analyses and visualization of DEGs were conducted using R 4.2.2 (https://www.r-project.org). RNA-seq data have been deposited in GEO: GSE304376.

### NRF2 reporter assay

The *Firefly* and *Renilla* dual-luciferase system-based NRF2 reporter assays were performed as described previously (31, 77). The pNQO1-ARE (antioxidant response element)-*Firefly* luciferase plasmid, kindly provided by Dr. Masayuki Yamamoto (Tohoku University), expresses the *Firefly* luciferase gene driven by NRF2 activation. The pRL-TK (thymidine kinase)-*Renilla* luciferase vector (Promega, Madison, WI, USA), which expresses the *Renilla* luciferase gene driven by the herpes simplex virus TK promoter, was used as an internal control for transfection efficiency. MDCK cells were seeded in 96-well plates (1 × 10^4^ cells/well) and co-transfected with pNQO1-ARE-*Firefly* luciferase (50 ng) and pRL-TK-*Renilla* luciferase (50 ng) at 37°C under 5% CO_2_ using Lipofectamine 2000 transfection reagent (Thermo Fisher Scientific), according to the manufacturer’s instructions. At 6 h post-transfection, the cells were treated with Mel56 (10 µM) in growth medium containing 10% FBS. DMSO (0.25%) and dl-sulforaphane (25 µM) were used as negative and positive controls, respectively. After 24 h of incubation at 37°C under 5% CO_2_, the treated cells were washed with PBS(–), and total RNA was extracted from cell lysates using an RNeasy Mini Kit (Qiagen). The mRNA expression levels of *Firefly* and *Renilla* luciferase were analyzed by RT-qPCR as described above. The primer sequences used for *Firefly* and *Renilla* luciferase are listed in Table S2. Relative gene expression was calculated using the ΔΔCt method. The expression levels of *Firefly* and *Renilla* luciferase were normalized to *canine β-actin* mRNA levels.

### Preparation of mitochondria from rat livers

Mitochondria were prepared from the livers of young adult male Wistar rats following a previously reported differential centrifugation procedure (78). All experimental animals used in the present study were purchased from Japan SLC, Shizuoka, Japan. Euthanasia was performed by cervical dislocation. The final mitochondrial pellets were suspended in aliquots of medium containing 250 mM sucrose and 2 mM Tris-HCl at pH 7.4. The resulting suspension was used as a stock solution for mitochondria and kept on ice during the experiments. Protein concentrations were determined using the Biuret method with BSA (FUJIFILM Wako Pure Chemical) as a standard.

### Inhibitory effects of Mel56 on mitochondrial ATP synthesis

The rate of ATP synthesis in the reaction mixture was measured by calculating the pH change in the incubation medium reported previously (43). Briefly, after 2.5 mM succinate addition (plus 0.23 µg/mL rotenone) as a respiratory substrate, mitochondria were suspended in 2.2 mL of medium (3 mM potassium phosphate buffer containing 200 mM sucrose, 20 mM KCl, and 3 mM MgCl_2_ at pH 7.4) to give a 0.2 mg/mL protein concentration. The mitochondria were treated with Mel56 (10 µM), antimycin A (1 µM; Sigma-Aldrich), oligomycin (1 µg/mL; Sigma-Aldrich), carboxyatractyloside (1 µM; Sigma-Aldrich), and SF6847 (100 nM; Tokyo Chemical Industry, Tokyo, Japan) as test compounds. As individual compounds were handled as DMSO solutions, the effect of DMSO on pH was also examined. Mitochondrial ATP synthesis was then initiated by the addition of 90 µM ADP. Baseline ATP synthesis was measured in the experiment. ATP synthesis in mitochondria can be determined by measuring the pH of the incubation medium, as H^+^ is consumed during ATP synthesis, as shown below.


$$ADP+Pi+H^{+}\to ATP+H_{2}O$$

The incubation time-dependent pH change of the reaction mixture was monitored using the electrode model PCE108CW-SR (Tokyo Chemicals, Tokyo, Japan) and recorded at 25°C. The pH change was calibrated by the addition of oxalic acid to make a final concentration of 45 µM.

### Inhibitory effects of Mel56 on the mitochondrial electron transport system

The inhibitory effect of Mel56 on the mitochondrial electron transport system was determined by measuring the oxygen consumption rate in the reaction mixture, as reported previously (43). Briefly, the mitochondria were suspended in an appropriate amount of medium (3 mM potassium phosphate buffer containing 200 mM sucrose, 20 mM KCl, and 3 mM MgCl_2_ at pH 7.4). After the addition of DMSO (0.1%) or Mel56 (10 µM) to the medium containing 2.5 mM succinate (plus 0.23 µg/mL rotenone), SF6847 (100 nM; Tokyo Chemical Industry) was added. The incubation time-dependent oxygen consumption of the reaction mixture was monitored at 25°C using the Clark-type oxygen electrode model 5331 (Yellow Springs Instrument, Yellow Springs, OH, USA).

### Evaluation of Mel56 permeabilization effect on mitochondrial inner membrane

Permeabilization effects of these compounds on the inner mitochondrial membrane were determined by measuring the oxygen consumption rate in the reaction mixture, as reported previously (43). Briefly, the mitochondria were suspended in an appropriate amount of medium (3 mM potassium phosphate buffer containing 200 mM sucrose, 20 mM KCl, and 3 mM MgCl_2_ at pH 7.4). After the addition of DMSO (0.1%), SF6847 (100 nM), or Mel56 (10 µM) to the medium containing 2.5 mM succinate (plus 0.23 µg/mL rotenone), the incubation time-dependent oxygen consumption of the reaction mixture was monitored at 25°C using the Clark-type oxygen electrode model 5331 (Yellow Springs Instrument, OH, USA).

### Detection of mitochondrial membrane potential in live MDCK cells

To monitor mitochondrial membrane potential in live MDCK cells, a JC-1 MitoMP Detection Kit (Dojindo, Kumamoto, Japan) was used. MDCK cells were seeded either in 35 mm glass-bottom dishes with 14 mm micro-wells (Matsunami, Osaka, Japan) at 3 × 10^4^ cells/well or in a 96-well optical-bottom black plate (Thermo Fisher Scientific) at 1 × 10^4^ cells/well and allowed to adhere for 24 h at 37°C under 5% CO_2_. Cells cultured in 35 mm glass-bottom dishes were stained with JC-1 (4 µM; Dojindo) and Hoechst 33342 (10 µM; Dojindo) for cell nuclear staining in growth medium. Cells cultured in 96-well optical-bottom black plates were stained with JC-1 (4 µM; Dojindo) alone. The cells were incubated for 1 h at 37°C under 5% CO_2_. After washing twice with growth medium, the cells were treated with Mel56 (10 µM–20 µM) in growth medium containing 10% FBS. DMSO (0.2%) and antimycin A (10 µM; Sigma-Aldrich) were used as negative and positive controls, respectively. After 2 h of incubation at 37°C under 5% CO_2_, the culture medium was replaced with 1 × imaging buffer included in the JC-1 MitoMP Detection Kit (Dojindo). Live cells in 35 mm glass-bottom dishes were observed using a fluorescence microscope (BIOREVO BZ-X700; Keyence), and images of JC-1 green, JC-1 red, and Hoechst 33342 (blue, nuclei) fluorescence were captured. Fluorescence intensities of JC-1 green and red in live cells cultured in 96-well plates were measured using an Infinite 200 PRO M Plex (Tecan, Zurich, Switzerland) at excitation/emission wavelengths of 485/535 nm (green) and 535/595 nm (red), respectively. The relative mitochondrial membrane potential is expressed as the JC-1 red/green fluorescence intensity ratio.

### Detection of intracellular ROS or mtROS in live MDCK cells

To measure intracellular or mtROS in live MDCK cells, a Highly Sensitive DCFH-DA ROS Assay Kit (Dojindo) and MitoSOX Red mitochondrial superoxide indicator (Thermo Fisher Scientific) were used. MDCK cells were seeded in 35 mm glass-bottom dishes with 14 mm micro-wells (Matsunami) at 3 × 10^4^ cells/well or in a 96-well optical-bottom black plate (Thermo Fisher Scientific) at 1 × 10^4^ cells/well and allowed to adhere for 24 h at 37°C under 5% CO_2_. The cells were then treated with Mel56 (10 µM–20 µM) in growth medium containing 10% FBS for 2 h at 37°C under 5% CO_2_. DMSO (0.2%) and antimycin A (10 µM; Sigma-Aldrich) were used as negative and positive controls, respectively. After treatment, the cells were washed twice with pre-warmed Hank’s balanced salt solution (HBSS) containing calcium, magnesium, and glucose. Cells cultured in 35 mm glass-bottom dishes were stained with DCFH-DA (1:1,000 dilution; Dojindo) in 1× loading buffer (included in the DCFH-DA kit) or with MitoSOX Red (2.5 µM; Thermo Fisher Scientific) in HBSS containing calcium, magnesium, and glucose. Hoechst 33342 (10 µM; Dojindo) was added to each staining solution for nuclear visualization. Cells cultured in a 96-well optical-bottom black plate were stained with DCFH-DA (1:1,000 dilution) or MitoSOX Red (2.5 µM) alone. After incubation for 30 min at 37°C under 5% CO_2_, the cells were washed with HBSS containing calcium, magnesium, and glucose, and the medium was replaced with fresh HBSS containing calcium, magnesium, and glucose. Live cells in 35 mm glass-bottom dishes were observed using a fluorescence microscope (BIOREVO BZ-X700; Keyence), and images of DCFH-DA (green) or MitoSOX Red (red), along with Hoechst 33342 (blue, nuclei), were captured. Fluorescence intensities of DCFH-DA or MitoSOX Red in live cells cultured in 96-well plates were measured using an Infinite 200 PRO M Plex (Tecan) at excitation/emission wavelengths of 490/530 nm (green) or 510/580 nm (red), respectively.

### Preparation of lung organoids derived from hiPSC

To start differentiation, hiPSC colonies were treated with TrypLE Select Enzyme (Thermo Fisher Scientific) for 10 min at 37°C. After centrifugation, cells were seeded onto Matrigel Growth Factor-Reduced Basement Membrane (Corning, Inc., Corning, NY, USA)-coated cell culture plates (2.0 × 10^5^ cells/4 cm^2^) and cultured for 2 days. The differentiation of human lung organoids was performed in serum-free differentiation (SFD) medium, composed of DMEM/F12 (3:1) (FUJIFILM Wako Pure Chemical and Thermo Fisher Scientific) supplemented with N2 (FUJIFILM Wako Pure Chemical), B-27 Supplement Minus Vitamin A (Thermo Fisher Scientific), 50 μg/mL ascorbic acid (STEMCELL Technologies, Vancouver, Canada), 1 × GlutaMAX (Thermo Fisher Scientific), 1% monothioglycerol (FUJIFILM Wako Pure Chemical), 0.05% BSA (Sigma-Aldrich), and P/S (Thermo Fisher Scientific). During days 0–1 of differentiation, cells were cultured with SFD medium supplemented with 10 μM Y-27632 (FUJIFILM Wako Pure Chemical) and 100 ng/mL recombinant Activin A (R&D Systems, Minneapolis, MN, USA). During days 1–3 of differentiation, cells were cultured with SFD medium supplemented with 10 μM Y-27632 (FUJIFILM Wako Pure Chemical), 100 ng/mL recombinant Activin A (R&D Systems), and 1% FBS. Between days 3 and 5 of differentiation, cells were cultured in SFD medium supplemented with 1.5 μM dorsomorphin dihydrochloride (FUJIFILM Wako Pure Chemical) and 10 μM SB431542 (FUJIFILM Wako Pure Chemical) for 24 h, followed by SFD medium supplemented with 10 μM SB431542 and 1 μM Stemolecule Wnt inhibitor IWP2 for another 24 h. During days 5–12 of differentiation, cells were cultured with SFD medium supplemented with 3 μM CHIR99021 (FUJIFILM Wako Pure Chemical), 10 ng/mL human fibroblast growth factor (FGF) 10 (PeproTech, Cranbury, NJ, USA), 10 ng/mL human FGF7 (PeproTech), 10 ng/mL human bone morphogenetic protein (BMP) 4 (PeproTech), 20 ng/mL human epidermal growth factor (PeproTech), and all-trans retinoic acid (ATRA; Sigma-Aldrich). On day 12 of differentiation, the cells were dissociated and embedded in Matrigel Growth Factor-Reduced Basement Membrane (Corning) to generate organoids. During days 12–20 of differentiation, organoids were cultured in SFD medium containing 3 μM CHIR99021, 10 ng/mL human FGF10, 10 ng/mL human FGF7, 10 ng/mL human BMP4, and 50 nM ATRA. On day 20 of differentiation, the organoids were recovered from the Matrigel, and the resulting suspension of organoids (small free-floating clumps) was seeded onto thin Matrigel-coated cell culture plates. Organoids were recovered from the Matrigel matrix because the virus could not access the apical side of the epithelial cells if they remained embedded in the Matrigel. During days 20–30 of differentiation, organoids were cultured in SFD medium containing 50 nM dexamethasone (Selleck Chemicals, Houston, TX, USA), 0.1 mM 8-bromo-cAMP (Tocris Bioscience), and 0.1 mM 3-isobutyl-1-methylxanthine (FUJIFILM Wako Pure Chemical).

### SARS-CoV-2 strains

SARS-CoV-2 Omicron XDQ.1 (EPI_ISL_19110349) was isolated from nasopharyngeal swab samples of patients with COVID-19. This study was approved by the research ethics committee of Kyoto University (R2379-3). Viruses were replicated in TMPRSS2/Vero cells and stored at −80°C until use. The SARS-CoV-2 infection experiments were performed according to strict regulations in a biosafety level 3 facility at Kyoto University. Viral titers were measured using a median tissue culture infectious dose (TCID_50_) assay. TMPRSS2/Vero cells were seeded in 96-well cell culture plates (Thermo Fisher Scientific). Samples were serially diluted 10-fold from 10^−1^ to 10^−8^ in cell culture medium, transferred onto the cells, and incubated at 37°C with 5% CO_2_ for 96 h. Cytopathic effects were evaluated under a microscope. The TCID_50_/mL was calculated using the Reed-Muench method. The MOI used for the SARS-CoV-2 infection experiments was determined based on preliminary studies to ensure robust and reproducible viral replication while minimizing excessive cytopathic effects under the experimental conditions.

### Protocol for SARS-CoV-2 infection assays in 96- or 12-well plates

A schematic representation of the SARS-CoV-2 infection assay protocol is shown in Fig. S8A and Fig. 8A. Briefly, hiPSC-derived lung organoids were seeded in a 96-well plate (2 × 10^4^ cells/well) or in a 12-well plate (8 × 10^5^ cells/well) and treated with DMSO (0.2%), remdesivir (10 µM), or Mel56 (0.31 µM–10 µM) in growth medium containing 5% FBS, followed by infection with or without SARS-CoV-2 Omicron XDQ.1 (MOI 0.01). After 24 h of incubation, the culture medium was replaced with fresh medium supplemented with DMSO or Mel56. The lung organoids were analyzed at 48 and 72 hpi.

### Measuring SARS-CoV-2 copy numbers in hiPSC-derived lung organoids

Cell culture supernatants from lung organoids were mixed with an equal volume of 2× RNA lysis buffer (distilled water containing 0.4 U/μL SUPERase In RNase Inhibitor [Thermo Fisher Scientific], 2% Triton X-100 [Nacalai Tesque], 50 mM KCl, 100 mM Tris-HCl [pH 7.5], and 40% glycerol) and incubated at 25°C for 10 min. The mixture was diluted 10-fold with distilled water. For the quantification of SARS-CoV-2 RNA (N-Sarbeco), a One-Step TB Green PrimeScript PLUS RT-PCR Kit (Perfect Real Time) (Takara Bio, Shiga, Japan) was used on a QuantStudio 1 or QuantStudio 3 real-time PCR system (Thermo Fisher Scientific). Standard curves were generated using SARS-CoV-2 RNA (N gene, 10^5^ copies/μL) purchased from Nihon Gene Research Laboratories (Miyagi, Japan). The primer sequences are listed in Table S2.

### Cell viability analysis in Mel56-treated hiPSC-derived lung organoids using the WST-8 assay

Cell viability in non-infected lung organoids treated with DMSO (0.2%) or Mel56 (0.31 µM–10 µM) in growth medium containing 5% FBS was determined after 72 h of incubation using the WST-8 assay with Cell Count Reagent SF (Nacalai Tesque), according to the manufacturer’s instructions.

### SARS-CoV-2 N protein expression analysis in SARS-CoV-2-infected hiPSC-derived lung organoids using capillary-based immunoassays

The lung organoids were lysed in RIPA buffer (Thermo Fisher Scientific) containing a protease inhibitor mixture (Sigma-Aldrich). After centrifugation, supernatants were collected. Antibody-based protein quantification was performed using a Jess system (ProteinSimple, Tokyo, Japan) with a 12 kDa–230 kDa Separation Module (ProteinSimple) according to the manufacturer’s instructions. SARS-CoV-2 N and β-actin were detected using the antibodies listed in Table S1. Data were analyzed and visualized using Compass for Simple Western software (ProteinSimple).

### RT-qPCR analysis of SARS-CoV-2 and host gene expression in virus-infected hiPSC-derived lung organoids

SARS-CoV-2-infected hiPSC-derived lung organoids used in the infection assay described in Fig. 8A were washed with PBS(–), and total RNA was isolated from lung organoids using ISOGENE (NIPPON GENE, Tokyo, Japan), according to the manufacturer’s instructions. The mRNA expression levels of the SARS-CoV-2 N and human host genes were analyzed by RT-qPCR, as described above. The primer sequences used for the SARS-CoV-2 N and human host genes are listed in Table S2. Relative gene expression was calculated using the ΔΔCt method. The expression levels of SARS-CoV-2 N and human host genes were normalized to human glyceraldehyde-3-phosphate dehydrogenase (*hGAPDH*) mRNA levels.

### Hematoxylin and eosin staining of SARS-CoV-2-infected hiPSC-derived lung organoid sections

The lung organoids were fixed with 4% paraformaldehyde (FUJIFILM Wako Pure Chemical) for 15 min, harvested, and used to prepare paraffin sections. Paraffin embedding, tissue sectioning, and histological staining were performed at the Applied Medical Research Laboratory.

### SARS-CoV-2-infected cells in sections of hiPSC-derived lung organoids by IF staining

For IF staining, lung organoids were fixed with 4% paraformaldehyde (FUJIFILM Wako Pure Chemical) in PBS(–) at 4°C. Lung organoids were harvested and used to prepare paraffin sections (approximately 15 μm). Paraffin was removed using xylene, and the sections were rehydrated with different ethanol concentrations. Antigen retrieval was performed using 0.1% tTBS (10×) (pH 7.4) (Nacalai Tesque). The slides were incubated in Blocking One (Nacalai Tesque) for 10 min at 25°C to block nonspecific staining. SARS-CoV-2 N protein was detected using primary and secondary antibodies, as described in Table S1. The sections were washed, mounted with ProLong Glass Antifade Mountant with NucBlue Stain (Thermo Fisher Scientific) and DAPI (Nacalai Tesque), and analyzed using an inverted laser scanning confocal microscopy system (FV3000, Evident, Tokyo, Japan).

### CC_50_, IC_50_, and selectivity index determination

The SI was calculated using the formula: SI = CC_50_/IC_50_, where CC_50_ represents the 50% cytotoxic concentration and IC_50_ represents the 50% inhibitory concentration. CC_50_ values of Mel56 were determined by the WST-8 cell viability assay using MDCK cells, A549 cells, and hiPSC-derived lung organoids, as described above (Fig. S1C, S2A, and S8C). IC_50_ values against the infection with IAV strains A/PR/8/34, A/WSN/33, and A/Aichi/2/68 were calculated based on WB and IF analyses of viral NP expression in Mel56-treated MDCK or A549 cells infected with the respective IAV strains (Fig. 1C, E, and G; Fig. S2C, E, and G). For SARS-CoV-2 infection in hiPSC-derived lung organoids, IC_50_ values were determined based on qPCR analyses of viral RNA levels (Fig. S8B). Dose–response curves were generated, and all CC_50_ and IC_50_ values were calculated using GraphPad Prism version 10 (GraphPad Software, La Jolla, CA, USA). An SI value greater than 1.0 was considered indicative of greater antiviral efficacy than cytotoxicity in the respective cell model.

### Quantification and statistical analysis

All statistical analyses were performed using GraphPad Prism version 10 (GraphPad Software) and Microsoft Excel (Microsoft Corporation, Redmond, WA, USA). All results are presented as means ± standard errors of the mean. Differences between two groups were assessed using an unpaired *t*-test, whereas comparisons among more than two groups were evaluated using one-way analysis of variance, followed by Dunnett’s *post hoc* tests or Tukey’s *post hoc* tests, as appropriate. Differences were considered statistically significant at *P* < 0.05.

## Supplementary Material

Reviewer comments

## Data Availability

All data supporting the findings of this study are available in the article and supplemental information. Raw data corresponding to figures in the text (Fig. 1A, B, D, and F; 2A, C, and C; 4A; 7A, C, and E; and 8C, H, and I) and supplemental material (Fig. S2B, D, and F; S3A; and S4A and B) were deposited on Mendeley data at DOI 10.17632/j9p6t6z7td.1. RNA-seq data have been deposited at GEO: GSE304376.

## SUPPLEMENTAL MATERIAL

Supplemental figures and tables spectrum.03093-25-s0001.docx Fig. S1 to S15, and Tables S1 and S2.

Table S3 spectrum.03093-25-s0002.xlsx List of downregulated DEGs in influenza A virus-infected MDCK cells treated with Mel56, identified by RNA-seq.

Table S4 spectrum.03093-25-s0003.xlsx List of upregulated DEGs in influenza A virus-infected MDCK cells treated with Mel56, identified by RNA-seq.

OPEN PEER REVIEW reviewer-comments.pdf An accounting of the reviewer comments and feedback.

ASM does not own the copyrights to Supplemental Material that may be linked to, or accessed through, an article. The authors have granted ASM a non-exclusive, world-wide license to publish the Supplemental Material files. Please contact the corresponding author directly for reuse.
