# Supplementary material for: Host-directed antiviral strategy targeting prohibitins: Mel56 suppresses influenza A virus and severe acute respiratory syndrome coronavirus 2 via modulation of antioxidant pathways and mitochondrial function
Source: Microbiol Spectr. 2026 May 15;14(7):e03093-25. doi: 10.1128/spectrum.03093-25 (PMC13339898; doi:10.1128/spectrum.03093-25)
Supplement: Supplemental figures and tables — Fig. S1 to S15, and Tables S1 and S2. [file spectrum.03093-25-s0001.docx]

**Supplementary information**

**Host-Directed Antiviral Strategy Targeting Prohibitins: Mel56 Suppresses Influenza A Virus and Severe Acute Respiratory Syndrome Coronavirus 2 via Modulation of Antioxidant Pathways and Mitochondrial Function**

Masaki Shoji^1*^, Rina Hashimoto^2,3^, Canan G. Nebigil^4^, Kazuto Takegawa^5,6^, Itsuki Tomita^1^, Kensuke Nakaoka^1^, Momiji Ishikawa^1^, Yasufumi Matsumura^7^, Miki Nagao^7^, Tomoyuki Esumi^8^, Etsuhisa Takahashi^9^, Hiroshi Kido^9^, Yasuo Shinohara^5,6^, Laurent Désaubry^4^, Kazuo Takayama^2,3^ and Takashi Kuzuhara^1*^

^1^ Laboratory of Biochemistry, Faculty of Pharmaceutical Sciences, Tokushima Bunri University, Tokushima, Japan.

^2^ Department of Synthetic Human Body System, Medical Research Laboratory, Institute of Integrated Research, Institute of Science Tokyo, Tokyo, Japan.

^3^ Center for iPS Cell Research and Application (CiRA), Kyoto University, Kyoto, Japan.

^4^ Regenerative Nanomedicine Laboratory (UMR1260), INSERM-University of Strasbourg, Center of Research in Biomedicine of Strasbourg (CRBS), Strasbourg, France.

^5^ Institute for Genome Research, Institute of Advanced Medical Sciences, Tokushima University, Tokushima, Japan

^6^ Graduate School of Pharmaceutical Sciences, Tokushima University, Tokushima, Japan

^7^ Department of Clinical Laboratory Medicine, Graduate School of Medicine, Kyoto University, Kyoto, Japan.

^8^ Laboratory of Medicinal Chemistry, Faculty of Pharmaceutical Sciences at Kagawa, Tokushima Bunri University, Takamatsu, Japan.

^9^ Division of Enzyme Chemistry, Institute for Enzyme Research, Tokushima University, Tokushima, Japan.

**^*^**Corresponding authors:

Laboratory of Biochemistry, Faculty of Pharmaceutical Sciences, Tokushima Bunri University, 180, Yamashiro-cho, Tokushima-city, Tokushima 770-8514, Japan.

Tel.: +81 (88) 602-8477; Fax: +81 (88) 655-3051.

E-mail address: [masaki-shoji@ph.bunri-u.ac.jp](mailto:masaki-shoji@ph.bunri-u.ac.jp) (MS) and [kuzuhara@ph.bunri-u.ac.jp](mailto:kuzuhara@ph.bunri-u.ac.jp) (TK)


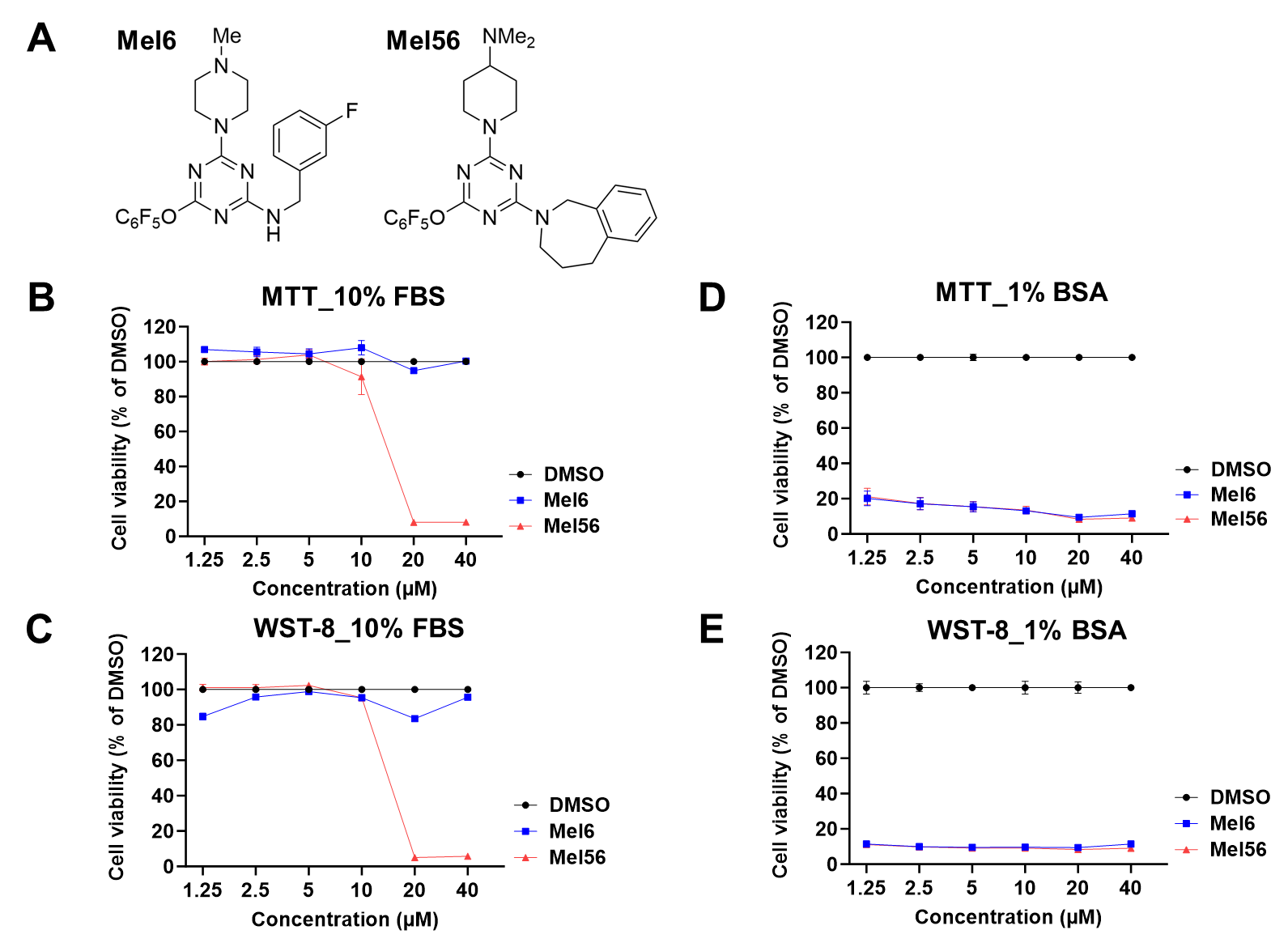
**Figure S1. Cytotoxicity of Mel6 and Mel56 in MDCK cells.** (A) Chemical structures of Mel6 and Mel56. (B–E) Cell viability of MDCK cells treated with the indicated concentrations of Mel6 or Mel56 (dissolved in DMSO) in growth medium containing 10% FBS (B, C) or serum-free medium containing 1% BSA (D, E), assessed using the MTT assay (B, D) or WST-8 assay (C, E). DMSO concentrations corresponded to compound concentrations as follows: 40 μM (0.4%), 20 μM (0.2%), 10 μM (0.1%), 5 μM (0.05%), 2.5 μM (0.025%), 1.25 μM (0.0125%). Data represent mean ± SEM (n = 4–17) and are representative of 2–3 independent experiments. Results were reproducible across experiments.

**
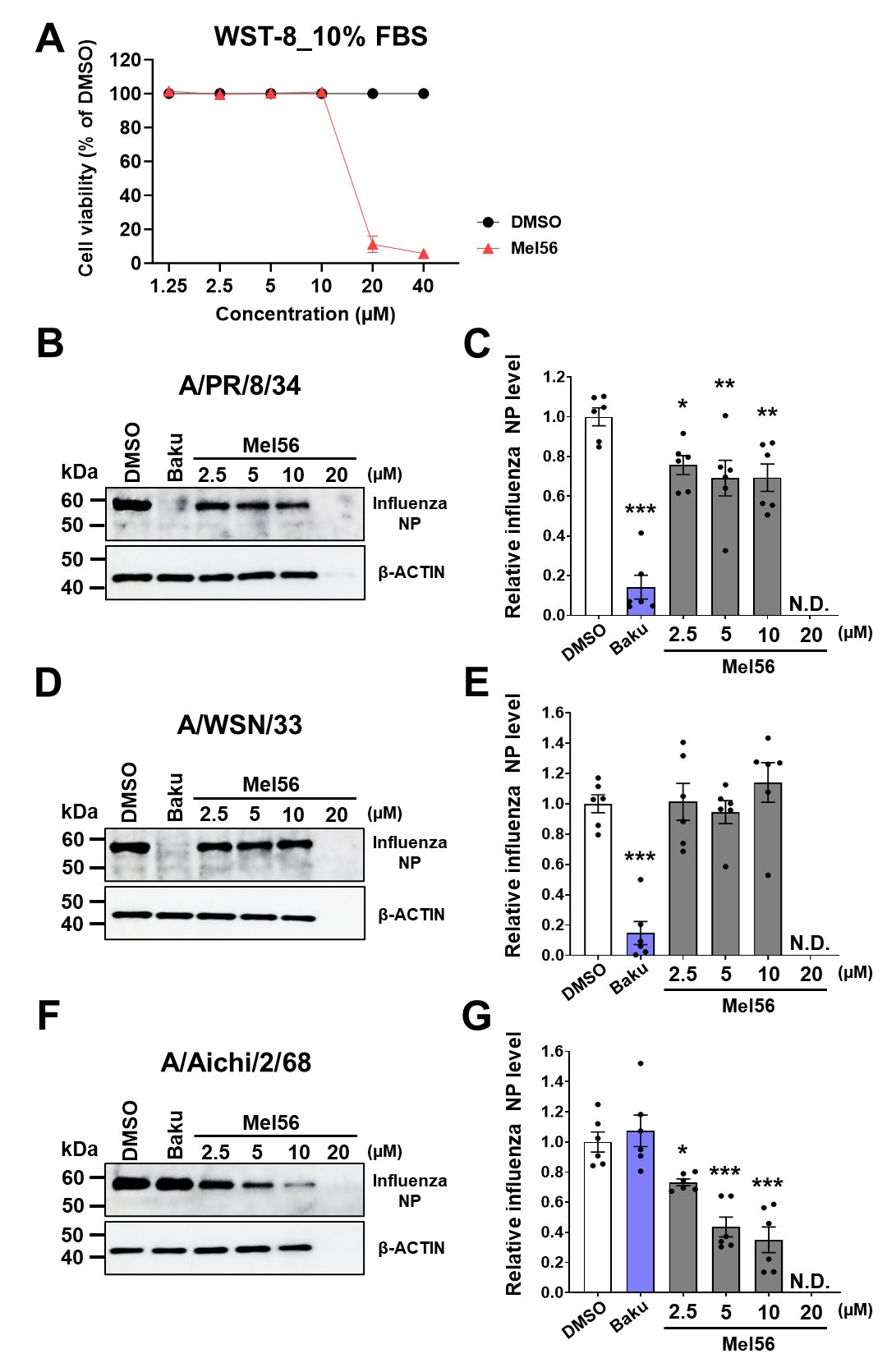
Figure S2. Mel56 reduces IAV NP expression in virus-infected A549 cells.** (A) Cell viability of MDCK cells treated with the indicated concentrations of Mel56 in growth medium containing 10% FBS was assessed using the WST-8 assay. The corresponding DMSO concentrations were as follows: 40 μM (0.4%), 20 μM (0.2%), 10 μM (0.1%), 5 μM (0.05%), 2.5 μM (0.025%), and 1.25 μM (0.0125%). Data represent the mean ± SEM (n = 5) from two independent experiments. (B–G) Western blot (WB) analysis of IAV NP expression in A549 cells infected with A/PR/8/34 (B, C), A/WSN/33 (D, E), or A/Aichi/2/68 (F, G) and treated with the indicated concentrations of Mel56 (2.5–20 μM) in growth medium containing 10% FBS. DMSO (0.2%) and baku (10 μM) were used as negative and positive controls, respectively. β-ACTIN was used as an internal control. NP expression levels were normalized to β-ACTIN and expressed relative to DMSO-treated controls (set to 1). Data represent the mean ± SEM (n = 6) from two independent experiments. N.D., not detected. *p < 0.05, **p < 0.01, ***p < 0.001 versus DMSO, as determined using one-way ANOVA followed by Dunnett’s post hoc tests.


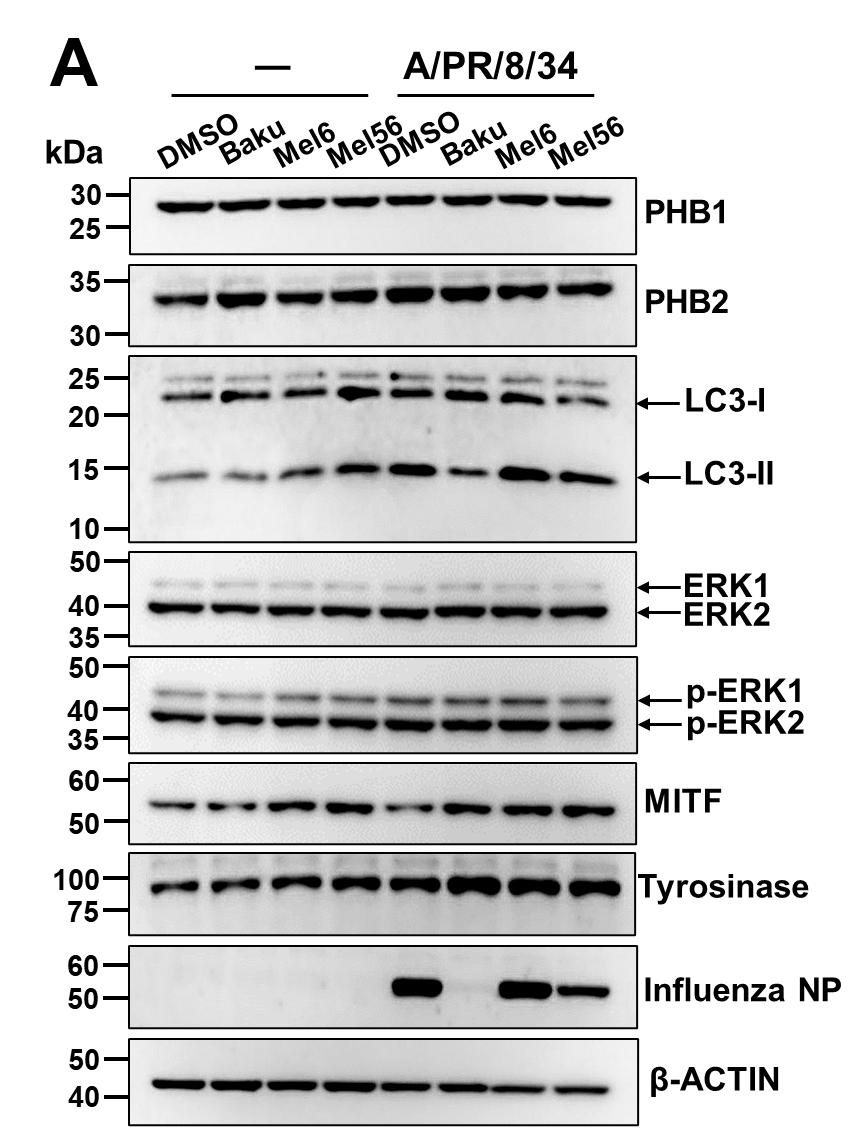


**
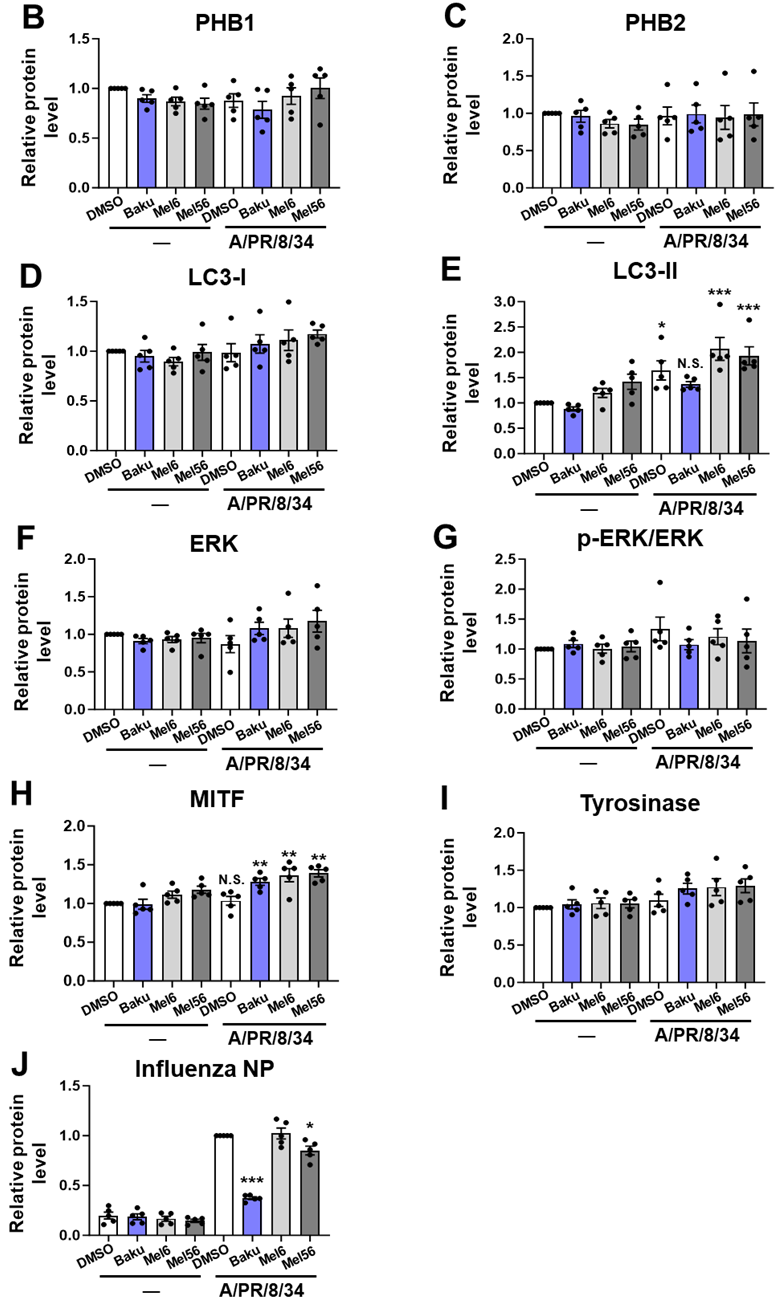
**

**Figure S3. WB analysis of MITF signaling in MDCK cells treated with Mel6 or Mel56.** (A) Protein expression levels of PHB1, PHB2, LC3-I, LC3-II, ERK1/2, p-ERK1/2, MITF, tyrosinase, influenza NP, and β-ACTIN in MDCK cells treated with DMSO (0.4%), Mel6 or Mel56 (2.5–10 µM). DMSO (0.1%) as negative control and baku (10 μM) as positive control, analyzed by Western blotting. β-ACTIN served as a loading control. (B–J) Quantification of PHB1 (B), PHB2 (C), LC3-I (D), LC3-II (E), ERK (F), p-ERK/ERK ratio (G), MITF (H), tyrosinase (I), and influenza NP (J). Protein levels were normalized to β-ACTIN and expressed relative to DMSO-treated cells without (B–I) or with (J) virus infection (set to 1). Data represent mean ± SEM (n = 5) and are representative of three independent experiments. *p < 0.05, **p < 0.01, ***p < 0.001 versus DMSO group, as determined using one-way ANOVA followed by Dunnett’s post hoc tests. N.S., not significant.

**
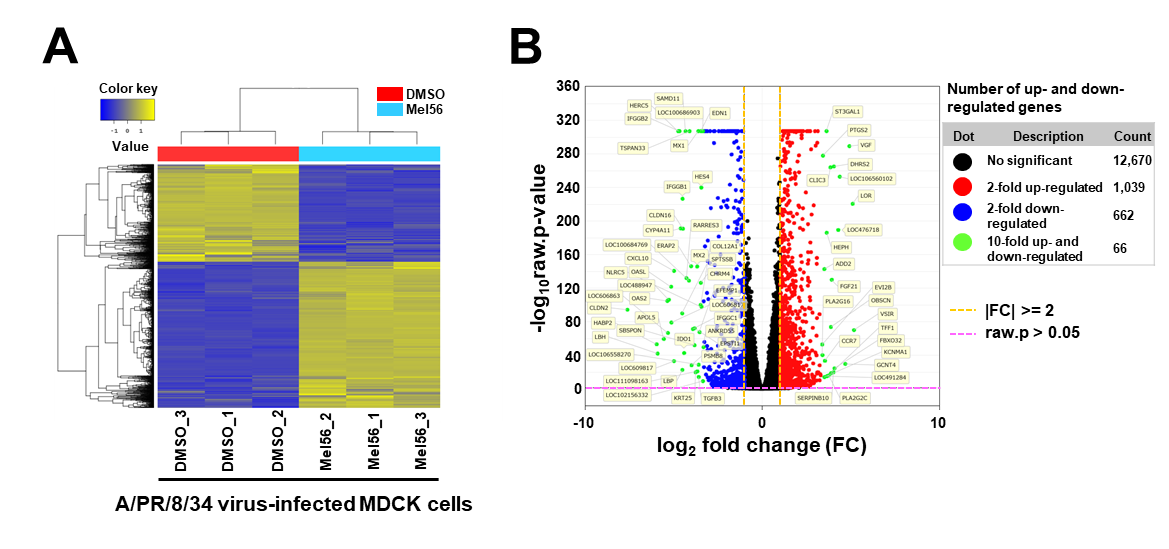
Figure S4. Transcriptomic profiling of Mel56-treated MDCK cells infected with IAV.** (A) Heatmap showing two-way hierarchical clustering of 1,767 DEGs (|fold change (FC)| ≥ 2, raw p < 0.05), based on Z-scores of log₂-transformed normalized expression values from RNA-seq data comparing Mel56- and DMSO-treated MDCK cells infected with A/PR/8/34 (n = 3). (B) Volcano plot of DEGs comparing Mel56- and DMSO-treated virus-infected cells. Red: upregulated ≥ 2-fold; blue: downregulated ≥ 2-fold; green: genes with ≥ 10-fold changes (both up and downregulated), with corresponding gene names labeled.

**
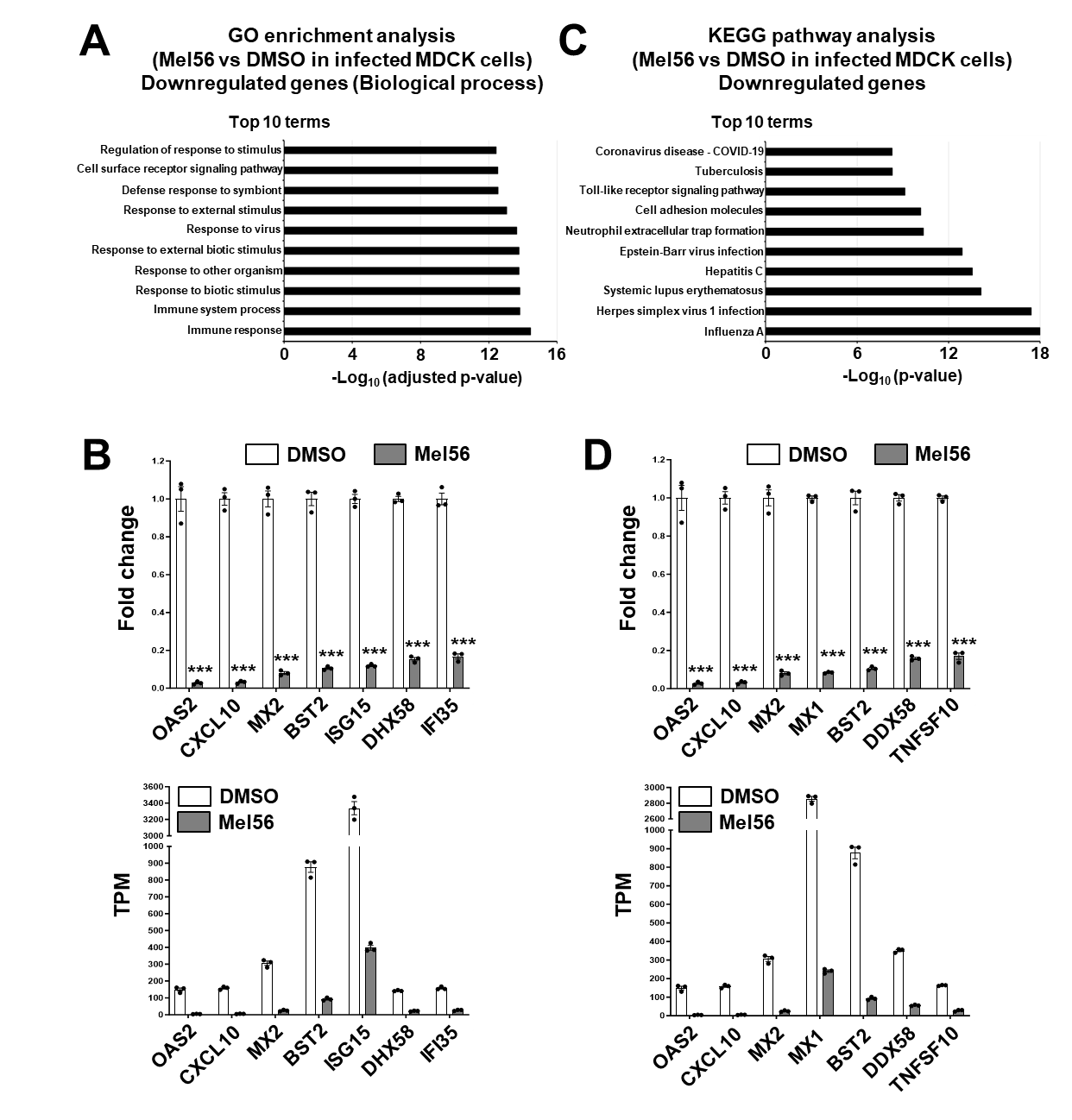
Figure S5. Transcriptomic analysis of downregulated DEGs in Mel56-treated MDCK cells infected with IAV.** (A) GO enrichment analysis of 609 downregulated DEGs in Mel56-treated MDCK cells infected with A/PR/8/34 identified by RNA-seq. Top 10 enriched biological process (BP) terms are shown as −log₁₀ (adjusted p-value). (B) Upper: Fold-change of top seven downregulated genes in “immune response” and “immune system process” BP categories, relative to DMSO (set to 1). Lower: TPM values of the same genes. Data are shown as mean ± SEM (n = 3). ***p < 0.001 versus DMSO, as determined using an unpaired *t*-test. (C) KEGG pathway analysis of similar DEGs, showing top 10 enriched pathways. (D) Upper: Fold-change of top seven downregulated genes associated with “Influenza A” and “Herpes simplex virus 1 infection”. Lower: Corresponding TPM values. ***p < 0.001 versus DMSO, as determined using an unpaired *t*-test. Full gene lists are provided in Table S3. Genes shown in panels B and D were selected based on criteria: |FC| ≥ 2 and TPM ≥ 100.

**Fi
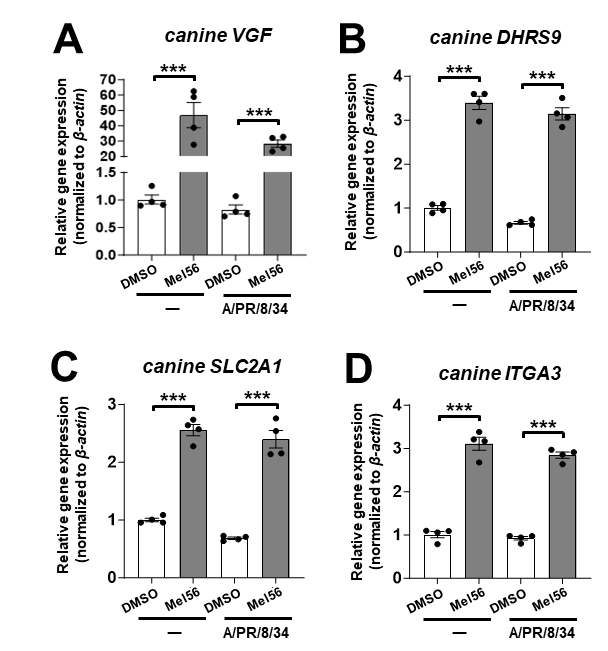
gure S6. RT-qPCR validation of genes identified by transcriptomic analysis of upregulated DEGs in Mel56-treated MDCK cells infected with IAV.** Relative mRNA expression levels of canine *VGF* (A), *DHRS9* (B), *SLC2A1* (C), and *ITGA3* (D) were analyzed by RT-qPCR in Mel56-treated MDCK cells infected with IAV. Gene expression was normalized to *canine β-actin* and expressed relative to DMSO-treated, virus-free controls (set to 1). Data represent mean ± SEM (n = 4). Statistical significance was determined via unpaired *t*-test; ***p < 0.001 for the indicated comparisons.

**
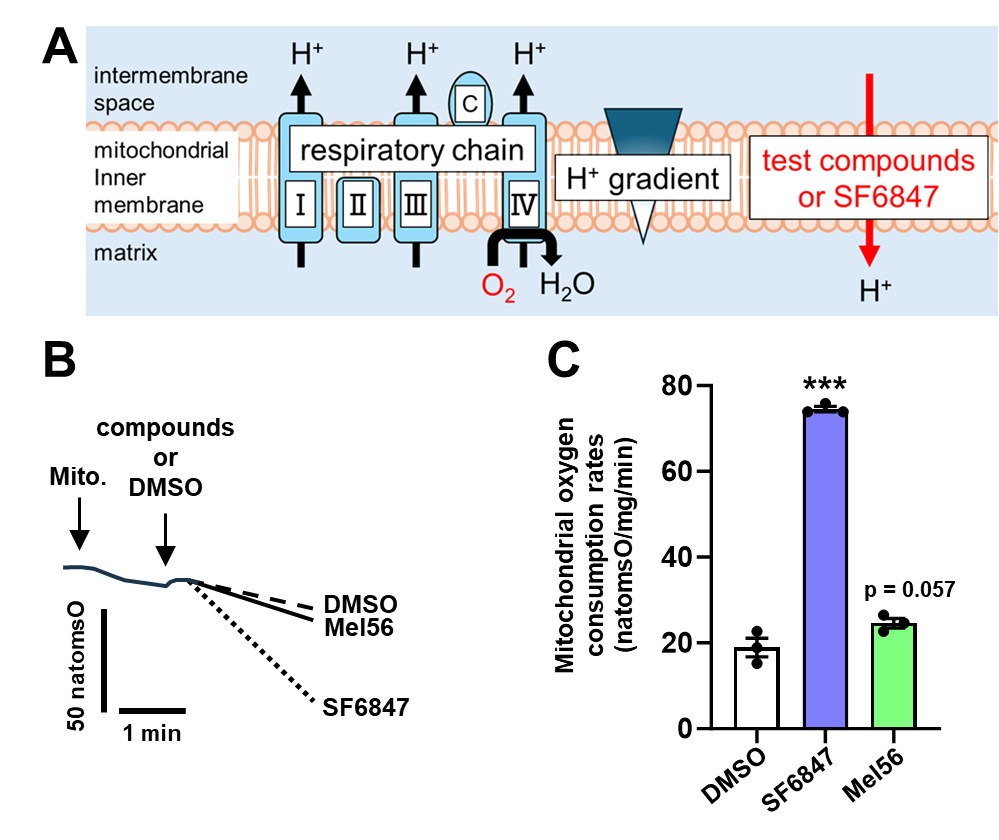
Figure S7. Effect of Mel56 on mitochondrial inner membrane permeability.** (A) Schematic illustration of the proposed model for mitochondrial inner membrane permeabilization induced by SF6847 or Mel56. (B) Time-dependent decrease in oxygen concentration in mitochondrial suspensions treated with DMSO (0.1%), SF6847 (100 nM), or Mel56 (10 µM). (C) Mitochondrial oxygen consumption rates (natomsO/mg/min) under each treatment condition. DMSO control: 19 ± 3 natomsO/mg/min. Data represent mean ± SEM (n = 3) and are representative of three independent experiments. ***p < 0.001 or p = 0.057 versus DMSO, as determined using one-way ANOVA followed by Dunnett’s post hoc tests.


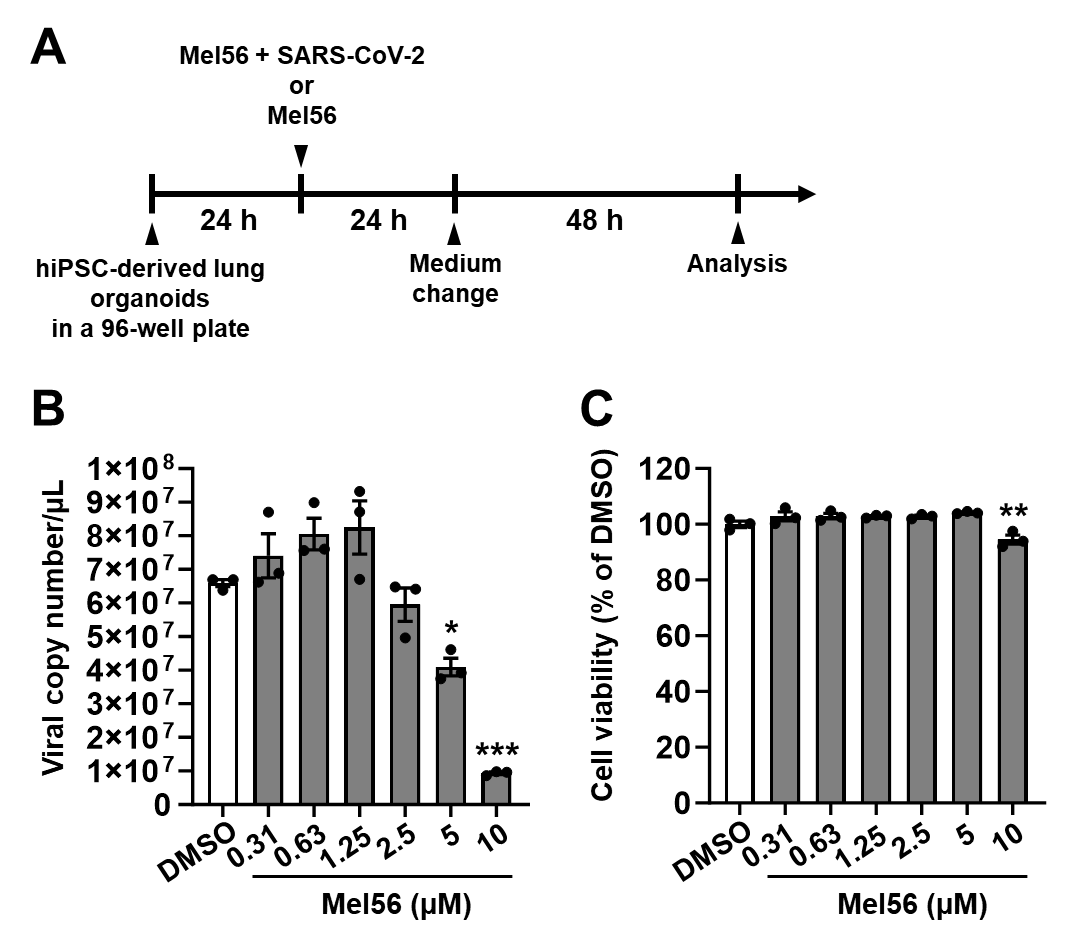
**Figure S8. Dose-dependent antiviral effect of Mel56 against SARS-CoV-2 in hiPSC-derived lung organoids.** (A) Schematic representation of the infection protocol in a 96-well plate. hiPSC-derived lung organoids (2 × 10⁴ cells/well) were treated with Mel56 (0.31–10 µM) and infected with or without SARS-CoV-2 Omicron XDQ.1 (MOI 0.01) in growth medium containing 5% FBS. DMSO (0.2%) served as a negative control. After 24 hours, the medium was replaced with fresh medium containing the respective compounds. (B) Viral RNA levels in the culture supernatant at 72 hpi, measured by RT-qPCR. (C) Cell viability in uninfected lung organoids treated with DMSO or Mel56, assessed using the WST-8 assay. Data represent mean ± SEM (n = 3) and are representative of two independent experiments. *p < 0.05, **p < 0.01, ***p < 0.001 versus DMSO group (infected or uninfected), as determined using one-way ANOVA followed by Dunnett’s post hoc tests.

**
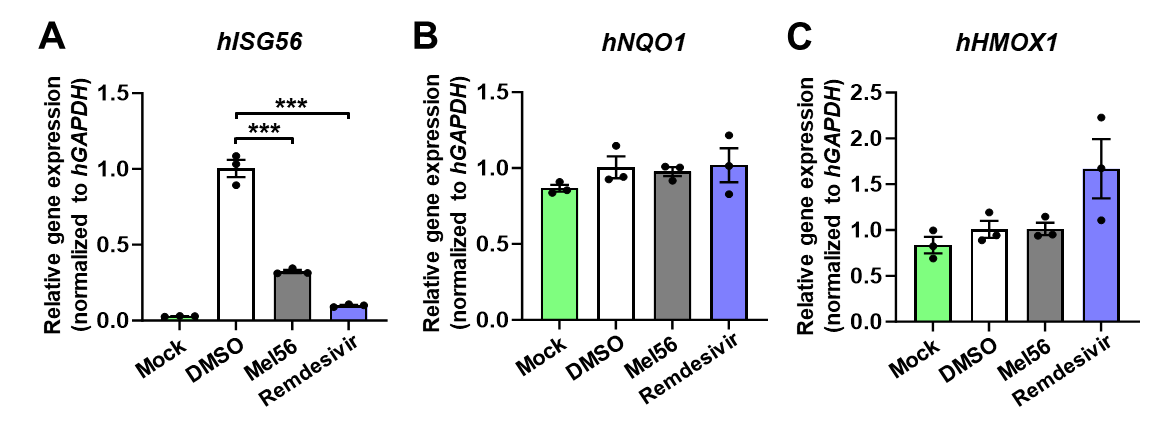
Figure S9. RT-qPCR analysis of antiviral host and NRF2 target genes in SARS-CoV-2-infected hiPSC-derived lung organoids.** mRNA expression levels of the antiviral host gene *hISG56* (A) and NRF2 target genes *hNQO1* (B) and *hHMOX1* (C) in SARS-CoV-2-infected lung organoids were analyzed by RT-qPCR. *hGAPDH* was used for normalization. “Mock” denotes uninfected and untreated controls. Data represent mean ± SEM (n = 3) from two independent experiments. ***p < 0.001 versus DMSO, as determined using one-way ANOVA followed by Dunnett’s post hoc tests.

**
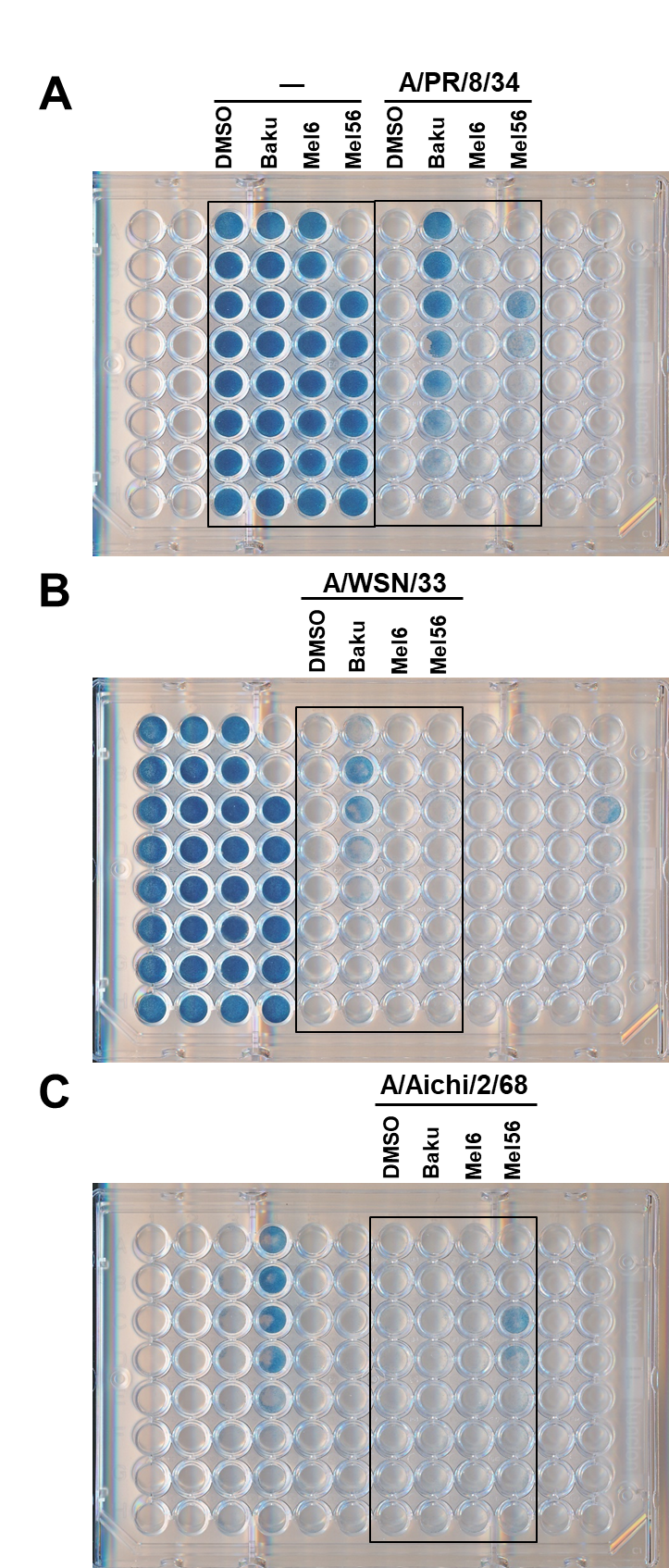
Figure S10. Uncropped images of NB-stained 96-well plates corresponding to Figure 1A.** MDCK cells were subjected to NB staining to assess viability following treatment with the indicated compound concentrations in the absence (–) (A) or presence of influenza A/PR/8/34 (A), A/WSN/33 (B), or A/Aichi/2/68 (C) viruses. The black squares shown in Figure 1A are derived from these plates.


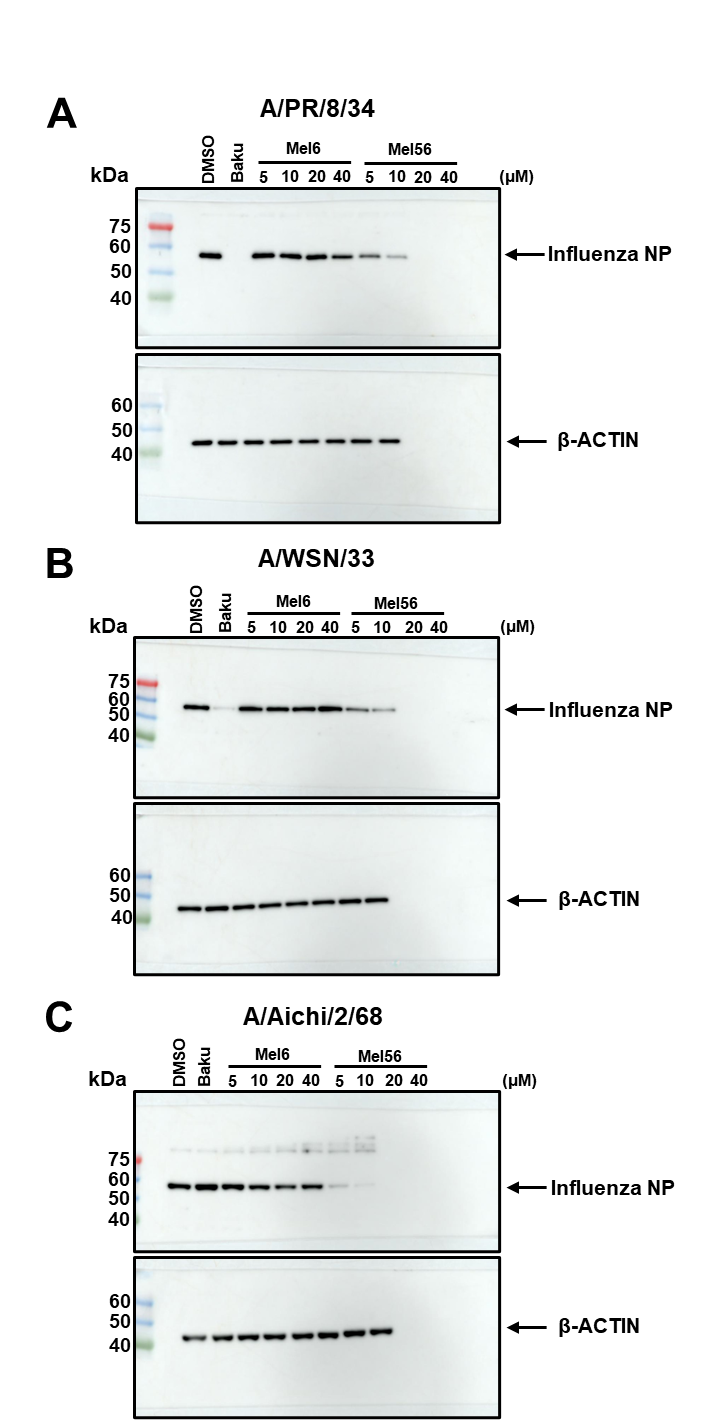
**Figure S11. Uncropped western blot membranes corresponding to Figures 1B, 1D, and 1F.** The membranes show influenza NP and β-ACTIN expression in MDCK cells infected with A/PR/8/34 (A), A/WSN/33 (B), or A/Aichi/2/68 (C) and treated with the indicated compound concentrations. The protein bands presented in Figures 1B, 1D, and 1F were derived from these membranes.


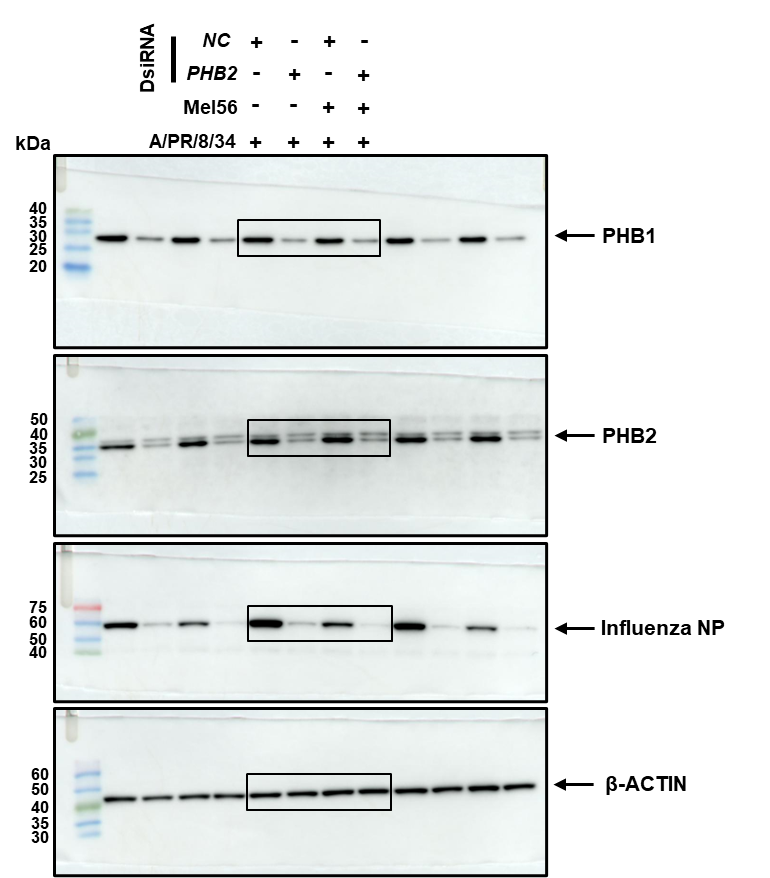
**Figure S12. Uncropped western blot membranes corresponding to Figure 4A.** The membranes show PHB1, PHB2, influenza NP, and β-ACTIN expression in MDCK cells transfected with *PHB2* DsiRNA or *NC* DsiRNA, treated with Mel56 (10 µM), and infected with A/PR/8/34 virus. The protein bands in black squares presented in Figure 4A were derived from these membranes.


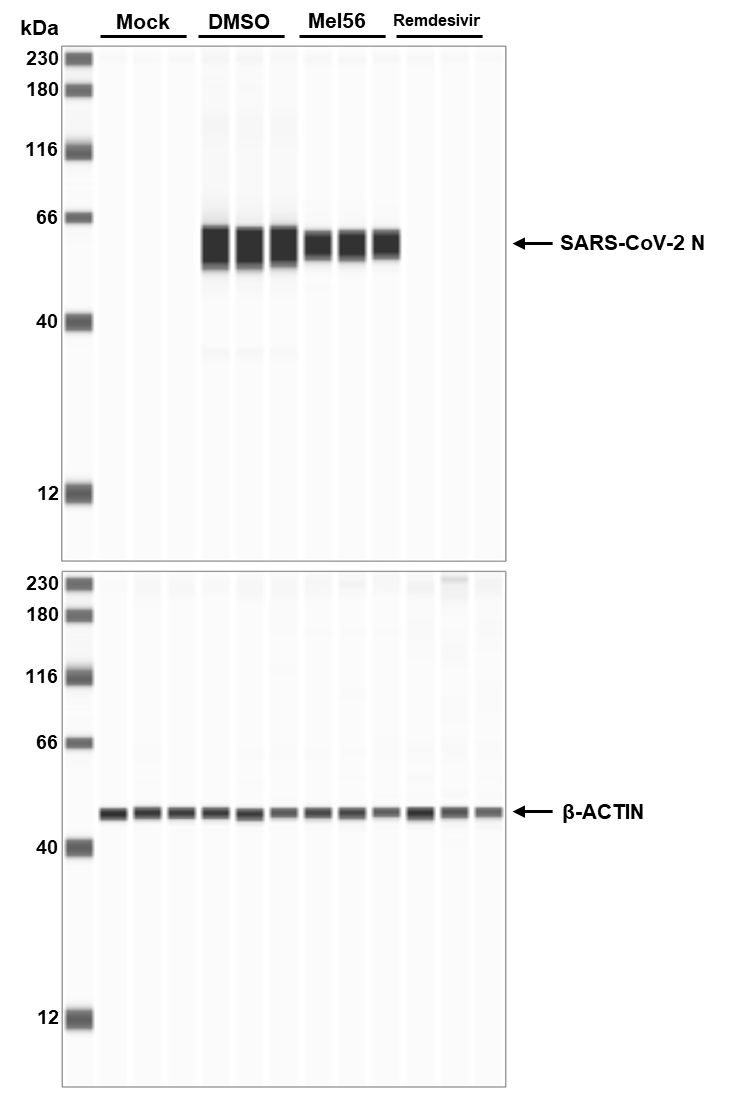
**Figure S13. Uncropped protein band data obtained by capillary-based immunoassay (Jess system) corresponding to Figure 8C.** The data show SARS-CoV-2 N and β-ACTIN expression in hiPSC-derived lung organoids infected with SARS-CoV-2 Omicron XDQ.1 and treated with the indicated compounds. The protein bands presented in Figure 8C were derived from these data.


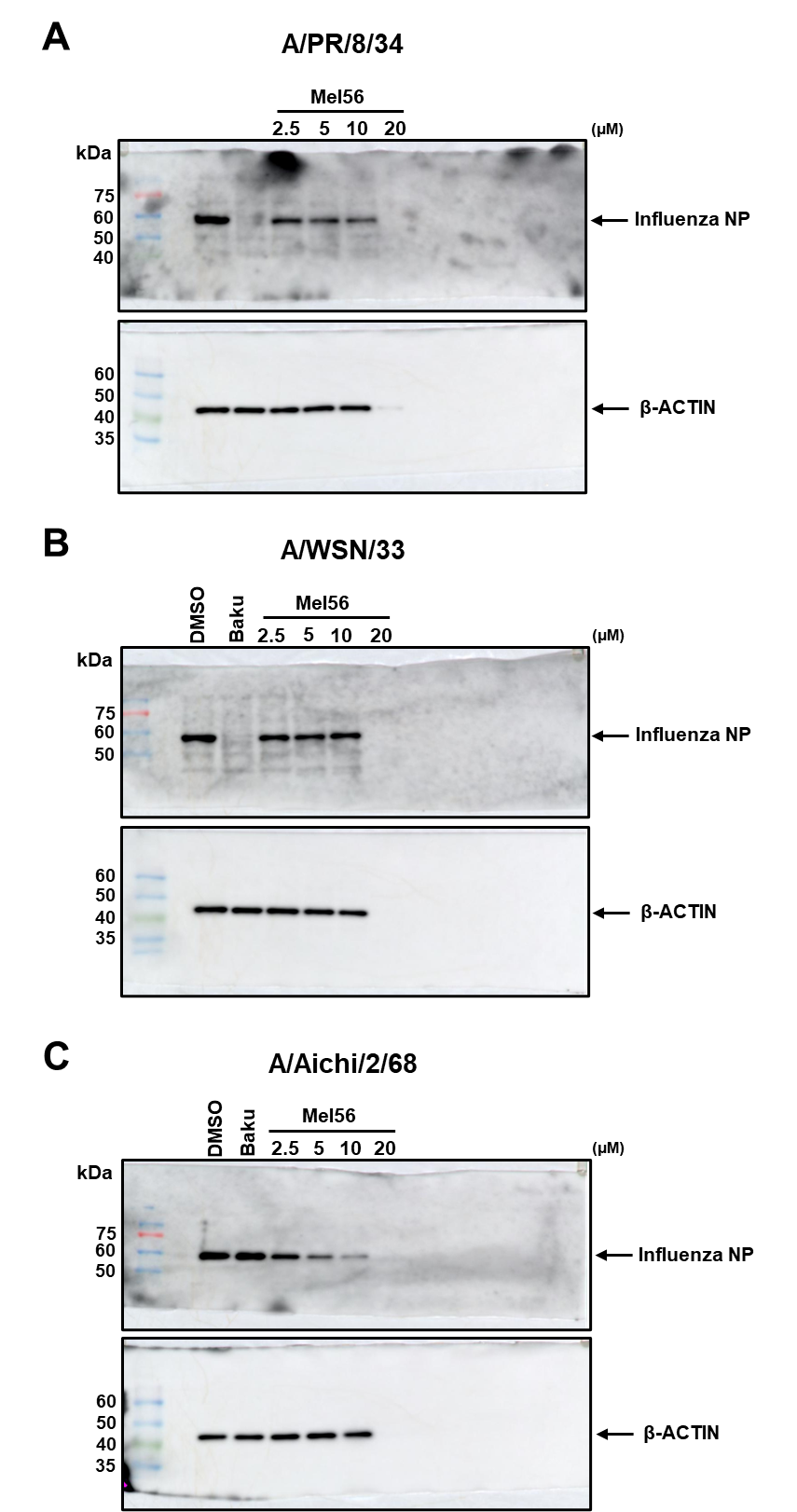
**Figure S14. Uncropped western blot membranes corresponding to Figures S2B, S2D, and S2F.** The membranes show influenza NP and β-ACTIN expression in A549 cells infected with A/PR/8/34 (A), A/WSN/33 (B), or A/Aichi/2/68 (C) and treated with the indicated compound concentrations. The protein bands presented in Figures S2B, S2D, and S2F were derived from these membranes.


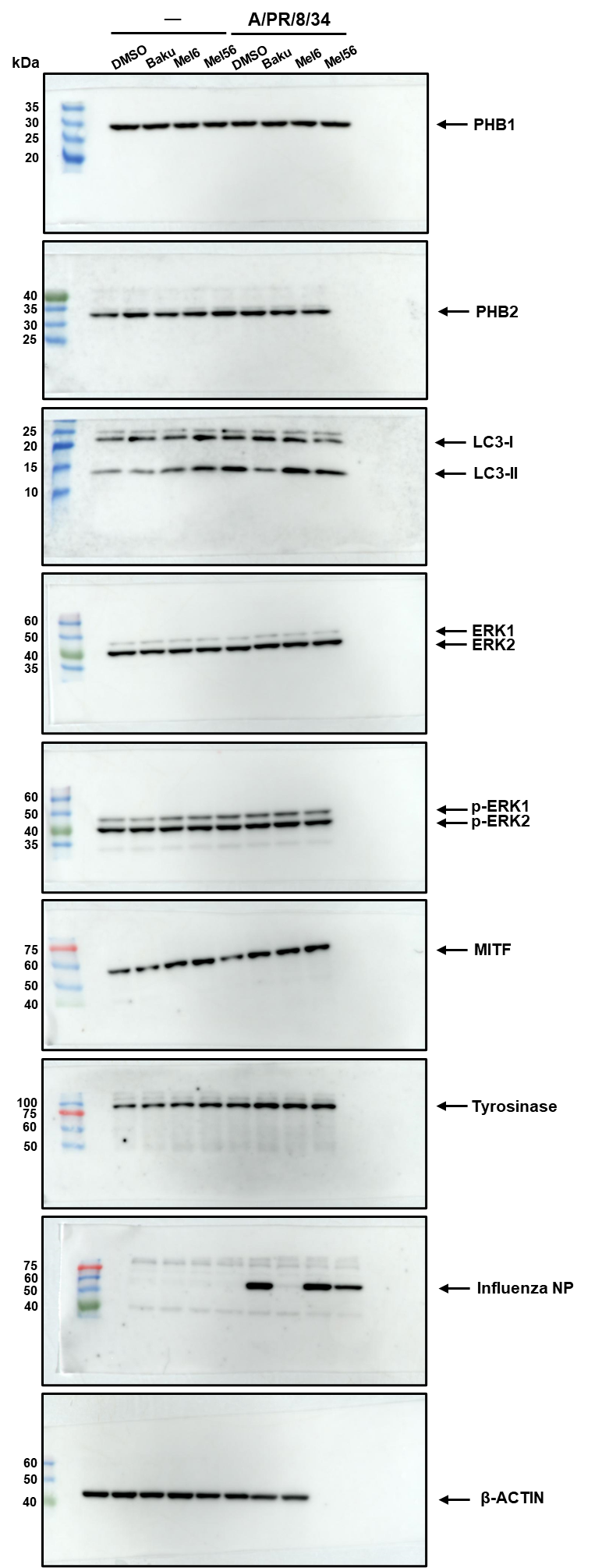
**Figure S15. Uncropped western blot membranes corresponding to Figure S3A.** The membranes show PHB1, PHB2, LC3-I, LC3-II, ERK1/2, p-ERK1/2, MITF, tyrosinase, influenza NP, and β-ACTIN expression in MDCK cells infected with A/PR/8/34 and treated with the indicated compounds. The protein bands in black squares presented in Figure S3A were derived from these membranes.

**Table S1. Primary and secondary antibodies.**

WB: Western blotting

IF: Immunofluorescence staining

Jess: A capillary-based immunoassay using Jess system

**Table S2. Quantitative real-time PCR primer sequences.**

A/PR/8/34, influenza A Puerto Rico 8/34; NP, nucleoprotein; NS1, nonstructural protein 1; PA, PB1 and PB2, RNA polymerase subunits; M1, matrix protein 1; M2, matrix protein 2; Ifn-b, interferon-b; Mx1, MX dynamin like GTPase 1; VGF, VGF nerve growth factor inducible; DHRS2, dehydrogenase/reductase 2; S100A14, S100 calcium binding protein A14; NDRG1, N-myc downstream regulated 1; DHRS9, dehydrogenase/reductase 9; SLC2A1, solute carrier family 2 member 1; ITGA3, integrin subunit alpha 3; NQO1, NAD(P)H quinone dehydrogenase 1; N-sarbeco, Charité's nucleocapsid; IFNβ1, interferon-β1; ISG15, interferon-stimulated gene 15; ISG56, interferon-stimulated gene 56; HMOX1, heme oxygenase 1; GAPDH, glyceraldehyde-3-phosphate dehydrogenase.
