# Supplementary material for: Host-directed antiviral strategy targeting prohibitins: Mel56 suppresses influenza A virus and severe acute respiratory syndrome coronavirus 2 via modulation of antioxidant pathways and mitochondrial function
Source: Microbiol Spectr. 2026 May 15;14(7):e03093-25. doi: 10.1128/spectrum.03093-25 (PMC13339898; doi:10.1128/spectrum.03093-25)
Supplement: Reviewer comments [file reviewer-comments.pdf]

AMERICAN SOCIETY FOR MICROBIOLOGY | Microbiology Spectrum

Host-Directed Antiviral Strategy Targeting Prohibitins: Mel56 Suppresses Influenza A Virus and Severe Acute Respiratory Syndrome Coronavirus 2 via Modulation of Antioxidant Pathways and Mitochondrial Function

Masaki Shoji, Rina Hashimoto, Canan Nebigil, Kazuto Takegawa, Itsuki Tomita, Kensuke Nakaoka, Momiji Ishikawa, Yasufumi Matsumura, Miki Nagao, Tomoyuki Esumi, Etsuhisa Takahashi, Hiroshi Kido, Yasuo Shinohara, Laurent Désaubry, Kazuo Takayama, and Takashi Kuzuhara

Corresponding Author(s): Takashi Kuzuhara, Tokushima Bunri Daigaku

Review Timeline:

Submission Date:	September 26, 2025
Editorial Decision:	November 16, 2025
Revision Received:	March 11, 2026
Editorial Decision:	March 29, 2026
Revision Received:	April 2, 2026
Accepted:	April 9, 2026

Editor: Yunyu Chen

Reviewer(s): Disclosure of reviewer identity is with reference to reviewer comments included in decision letter(s). The following individuals involved in review of your submission have agreed to reveal their identity: Shuofeng Yuan (Reviewer #2)

Transaction Report:

(Note: With the exception of the correction of typographical or spelling errors that could be a source of ambiguity, letters and reports are not edited. The original formatting of letters and referee reports may not be reflected in this compilation.)

DOI: https://doi.org/10.1128/spectrum.03093-25

Re: Spectrum03093-25 (**Host-Directed Antiviral Strategy Targeting Prohibitins: Mel56 Suppresses Influenza A Virus and SARS-CoV-2 via modulation of antioxidant pathways and mitochondrial function**)

Dear Prof. Takashi Kuzuhara:

Thank you for the privilege of reviewing your work. Your manuscript has now been reviewed by expert reviewers for the journal. The reviewers have raised some important issues and have made many useful suggestions for its improvement. Based on these comments (attached below), as well as my own review, we would be happy to receive a revised version of the manuscript that is responsive to the reviewers' comments.

Please return the manuscript within 60 days; if you cannot complete the modification within this time period, please contact me. If you do not wish to modify the manuscript and prefer to submit it to another journal, notify me immediately so that the manuscript may be formally withdrawn from consideration by Spectrum.

Revision Guidelines
To submit your modified manuscript, log into the submission site at https://spectrum.msubmit.net/cgi-bin/main.plex. Go to Author Tasks and click the appropriate manuscript title to begin. The information you entered when you first submitted the paper will be displayed; update this as necessary. Note the following requirements:

• Upload point-by-point responses to the issues raised by the reviewers in a file named "Response to Reviewers," NOT in your cover letter.
• Upload a compare copy of the manuscript (without figures) as a "Marked-Up Manuscript" file.
• Upload a clean .DOC/.DOCX version of the revised manuscript and remove the previous version.
• Each figure must be uploaded as a separate, editable, high-resolution file (TIFF or EPS preferred), and any multipanel figures must be assembled into one file.
• Any supplemental material intended for posting by ASM should be uploaded with their legends separate from the main manuscript. You can combine all supplemental material into one file (preferred) or split it into a maximum of 10 files with all associated legends included.

For complete guidelines on revision requirements, see our Submission and Review Process webpage. Submission of a paper that does not conform to guidelines may delay acceptance of your manuscript.

Data availability: ASM policy requires that data be available to the public upon online posting of the article, so please verify all links to sequence records, if present, and make sure that each number retrieves the full record of the data. If a new accession number is not linked or a link is broken, provide Spectrum production staff with the correct URL for the record. If the accession numbers for new data are not publicly accessible before the expected online posting of the article, publication may be delayed; please contact production staff (Spectrum@asmusa.org) immediately with the expected release date.

Publication Fees: For information on publication fees and which article types are subject to charges, visit our website. If your manuscript is accepted for publication and any fees apply, you will be contacted separately about payment during the production process; please follow the instructions in that e-mail. Arrangements for payment must be made before your article is published.

ASM Membership: Corresponding authors may join or renew ASM membership to obtain discounts on publication fees. Need to upgrade your membership level? Please contact Customer Service at Service@asmusa.org.

The ASM Journals program strives for constant improvement in our submission and publication process. Please tell us how we can improve your experience by taking this quick Author Survey.

Thank you for submitting your paper to Spectrum.

Sincerely,
Yunyu Chen
Editor
Microbiology Spectrum

Reviewer #1 (Public repository details (Required)):

The study includes RNA-seq (transcriptomic) datasets, which should be deposited in a public repository such as GEO or SRA. Please provide accession numbers prior to publication.

Reviewer #1 (Comments for the Author):

This study investigates the antiviral role of PHB-binding triazine melanogenin derivative Mel56 and potential mechanisms. Mel56 is against influenza A virus (H1N1/H3N2) in MDCK cells and SARS-CoV-2 in human iPSC-derived lung organoids. This is the novel role of Mel56 and providing a promising host-directed therapeutic strategy against viral infection, which is with great significance. And the study employs transcriptomic analysis and human iPSC-derived lung organoids as the SARS-CoV-2 infection model as well as new technique like capillary-based immunoassay. However, some revisions are still required.

Major concerns:
1. In the figure 4, knocking down PHB2 significantly decreases PR8 NP expression, indicating PHB2 helps viral replication not against IAV infection. And this article showed PHB1 facilitates PR8 IAV replication. (Zou J, Tian S, Zhu Y, Cheng Y, Jiang M, Tu S, Jin M, Chen H, Zhou H.2023.Prohibitin1 facilitates viral replication by impairing the RIG-I-like receptor signaling pathway. J Virol97:e00926-23.https://doi.org/10.1128/jvi.00926-23)
Moreover, the data of this study supports Mel56 has antiviral activities but not support this antiviral role needs PHBs.
2. The last sentence in the first paragraph in page (line 219-222) says Mel56 may exert anti-influenza activity by suppressing host immune responses triggered by viral infections. The down-regulated innate immune genes only indicate that the antiviral function of Mel56 is by other mechanisms not by increasing the innate immune response.
3. The study showed Mel56 impairs mitochondrial function and transcriptome analysis indicated antioxidant gene expression is upregulated. But no experiments support the NRF2-ROS or NRF2 activation mechanism, the mechanism is speculative. Experiments like NRF2 inhibitor, activator or NRF2 siRNA, ROS assays may be need to include to have this conclusion.
4. The Mitochondrial assays performed in rat liver mitochondria which is not the same species as the actual cell models (MDCK cells and human organoids) and is lack relevance to cell infection biology.
5.SARS-CoV-2 data showed Mel 56 has the antiviral activity but no experiments to support it share the mechanism with IAV: no transcriptome analysis, no mitochondrial assays in organoids.

Minor concerns:
1. In line 67 and 70, the citation of the website is not like this way.
2. In line 142 "similar to the effects of Baku at 0.4-25 μM (A/PR/8/34) or 3. 1-25 μM (A/WSN/33) (Fig. 1A)" what does 0.4-25 μM (A/PR/8/34) or 3.1-25 μM (A/WSN/33) mean?
3. More detail is needed for the MOI selection rationale.

Reviewer #2 (Comments for the Author):

It is interesting to discover that host melanogenin derivative Mel56, but not Mel6, is against influenza A virus and SARS-CoV-2 in vitro. It seems that the antiviral function of Mel56 is relative to mitochondrial modulation and NRF2 activation. However, the conclusions drawn are often speculative and not fully supported by the data presented. Further mechanism study is required.

1. The manuscript's cytotoxicity data raise concerns about the compound's viability. The authors state Mel56 is non-cytotoxic at <10 μM in 10% FBS media, yet Figure S1 reveals severe cytotoxicity (>90%) at just 20 μM, indicating a very steep toxicity curve. More troubling is the low CC50 (<1.25 μM) observed in 1% BSA-containing medium. This high sensitivity to serum protein concentration complicates the interpretation of its antiviral effect. The authors must clarify which medium conditions (10% FBS or 1% BSA) were used for all subsequent antiviral assays.
2. The data in Figure 2 show that Mel56 treatment reduces the percentage of NP-positive cells but does not appear to diminish the NP signal intensity within cells that are already infected. This observation suggests a mechanism of action at an early stage of the viral life cycle, such as inhibition of viral entry. It is highly suggested to dissert which viral cycle step is affected by Mel56.
3. The study relies exclusively on MDCK cells. While a standard model, results from a canine kidney cell line may not be representative of the Mel56's activity in the human respiratory tract, the primary site of influenza infection. To establish physiological relevance, it is essential to validate the CC50 and EC50 values in human-derived lung epithelial cell lines (e.g., A549, BEAS-2B) and preferably in primary human bronchial epithelial (HBE) cell models.
4. At Fig. 3, the authors claim that Mel56 directly reduces the expression of Ifn-β and Mx1. This conclusion is confounded by Mel56's primary antiviral activity. It is difficult to determine from the current data whether Mel56 directly suppresses the host innate immune pathway or if the reduced Ifn-β/Mx1 expression is simply a secondary consequence of the reduced viral load. To de-convolute these two possibilities, the authors need to test Mel56's effect in a virus-free system, for example, by measuring Ifn-β/Mx1 induction following stimulation with poly(I:C).
5. At Fig. 4, the data presented are insufficient to conclude that PHB proteins are a functional target of Mel56's anti-influenza activity. If Mel56 and a separate PHB inhibition both act on same pathway, their combined effect should be redundant (non-additive). The additive effect suggested by the data would imply they act on independent targets/pathways. If to properly assess the pharmacodynamic relationship (i.e., synergistic), a checkerboard titration assay analyzing combination indices (CI) is required.
6. Fig. 5D shows that the expression levels of VGF, DHRS2, S100A14, NDRG1, DHRS9, SLC2A1, ITGA3, and NQO1 were significantly upregulated by Mel56.
However, the conclude that 'Mel56 attenuates virus-induced immune responses and enhances antioxidant gene expression,

potentially via NRF2 activation' is correlative and speculative. To link this pathway to the antiviral effect, its necessity must be demonstrated. The authors should test if the antiviral activity of Mel56 is abrogated in Nrf2 knockout/knockdown (KO/KD) cells or in the presence of an NRF2 inhibitor.
7. Figure 6 indicates that Mel56 disrupts mitochondrial function. Disruption of mitochondrial integrity is a common hallmark of cell poisoning. The authors must provide evidence to decouple this mitochondrial dysfunction from non-specific cytotoxicity and demonstrate that this effect is specific to inhibiting the virus, not killing the host cell.
8. The manuscript proposes two separate mechanisms (NRF2 activation and mitochondrial disruption) without a unifying link. A hypothesis mentioned at the discussion section is that mitochondrial disruption by Mel56 induces mitochondrial ROS (mtROS), which is a known upstream activator of the NRF2 pathway. It is strongly suggested to directly measure mtROS levels (e.g., via MitoSOX) following Mel56 treatment to test this hypothesis and connect the observations from Figures 5 and 6.

Editor comments:
1. In this study, the authors reported that Mel56 is a PHB binding compound with broad-spectrum antiviral activity, but how to support this conclusion via rigorous biochemical experiments? Is there a direct binding between the compound and the target? I think this is an important question for this research.
2. The antiviral role of Mel56 against influenza A virus (H1N1/H3N2) in MDCK cells and SARS-CoV-2 in human iPSC-derived lung organoids is a promising therapeutic target. but the mechanistic claims are overstated and not experimentally demonstrated. I recommend major revision with explicit softening of mechanistic interpretations.

To authors:

This study investigates the antiviral role of PHB-binding triazine melanogenin derivative Mel56 and potential mechanisms. Mel56 is against influenza A virus (H1N1/H3N2) in MDCK cells and SARS-CoV-2 in human iPSC-derived lung organoids. This is the novel role of Mel56 and providing a promising host-directed therapeutic strategy against viral infection, which is with great significance. And the study employs transcriptomic analysis and human iPSC-derived lung organoids as the SARS-CoV-2 infection model as well as new technique like capillary-based immunoassay. However, some revisions are still required.

Major concerns:

1. In the figure 4, knocking down PHB2 significantly decreases PR8 NP expression, indicating PHB2 helps viral replication not against IAV infection. And this article showed PHB1 facilitates PR8 IAV replication. *(Zou J, Tian S, Zhu Y, Cheng Y, Jiang M, Tu S, Jin M, Chen H, Zhou H.2023.Prohibitin1 facilitates viral replication by impairing the RIG-I-like receptor signaling pathway. J Virol97:e00926-23.https://doi.org/10.1128/jvi.00926-23)*
   Moreover, the data of this study supports Mel56 has antiviral activities but not support this antiviral role needs PHBs.
2. The last sentence in the first paragraph in page (line 219-222) says Mel56 may exert anti-influenza activity by suppressing host immune responses triggered by viral infections. The down-regulated innate immune genes only indicate that the antiviral function of Mel56 is by other mechanisms not by increasing the innate immune response.
3. The study showed Mel56 impairs mitochondrial function and transcriptome analysis indicated antioxidant gene expression is upregulated. But no experiments support the NRF2-ROS or NRF2 activation mechanism, the mechanism is speculative. Experiments like NRF2 inhibitor, activator or NRF2 siRNA, ROS assays may be need to include to have this conclusion.
4. The Mitochondrial assays performed in rat liver mitochondria which is not the same species as the actual cell models (MDCK cells and human organoids) and is lack relevance to cell infection biology.
5. SARS-CoV-2 data showed Mel 56 has the antiviral activity but no experiments to support it share the mechanism with IAV: no transcriptome analysis, no mitochondrial assays in organoids.

Minor concerns:

1. In line 67 and 70, the citation of the website is not like this way.
2. In line 142 “similar to the effects of Baku at 0.4–25 μM (A/PR/8/34) or 3.1–25 μM (A/WSN/33) (Fig. 1A)” what does 0.4–25 μM (A/PR/8/34) or 3.1–25 μM (A/WSN/33) mean?
3. More detail is needed for the MOI selection rationale.

Responses to Reviewers' comments

Manuscript Number: Spectrum03093-25

Masaki Shoji, Rina Hashimoto, Canan G. Nebigil, Kazuto Takegawa, Itsuki Tomita, Kensuke Nakaoka, Momiji Ishikawa, Yasufumi Matsumura, Miki Nagao, Tomoyuki Esumi, Etsuhisa Takahashi, Hiroshi Kido, Yasuo Shinohara, Laurent Désaubry, Kazuo Takayama and Takashi Kuzuhara

"**Host-Directed Antiviral Strategy Targeting Prohibitins: Mel56 Suppresses Influenza A Virus and Severe Acute Respiratory Syndrome Coronavirus 2 via Modulation of Antioxidant Pathways and Mitochondrial Function**"

Dear Professor Yunyu Chen,

We would like to thank you and the reviewers for the positive comments on our manuscript, "Host-Directed Antiviral Strategy Targeting Prohibitins: Mel56 Suppresses Influenza A Virus and Severe Acute Respiratory Syndrome Coronavirus 2 via Modulation of Antioxidant Pathways and Mitochondrial Function" which we would like to resubmit for publication. We appreciated the constructive suggestions provided by the reviewers. Based on this feedback, we have added new experimental data, including the validation of our findings using additional cell and lung organoid models and additional analyses of the effects of Mel56 on mitochondrial function, and revised the manuscript accordingly. The revised manuscript more effectively establishes support for the development of PHB-targeting ligands.

Please find our point-by-point responses to the comments of the reviewers (in *italics*) below.

We believe that the revised manuscript addresses the comments provided by reviewers and we hope that it is now suitable for publication in *Microbiology Spectrum*.

Thank you for your consideration. I look forward to hearing from you.

Sincerely yours,

Takashi Kuzuhara, Ph.D.

Professor, Laboratory of Biochemistry, Faculty of Pharmaceutical Sciences,
Tokushima Bunri University

180 Nishihamahouji, Yamashirocho, Tokushima-city 770-8514, Japan
Tel.: +81 (88) 602-8477, Fax: +81 (88) 655-3051, E-mail: kuzuhara@ph.bunri-u.ac.jp

Editor comments:

1. In this study, the authors reported that Mel56 is a PHB binding compound with broad-spectrum antiviral activity, but how to support this conclusion via rigorous biochemical experiments? Is there a direct binding between the compound and the target? I think this is an important question for this research.

Response 1:

We sincerely appreciate the important comment regarding the biochemical evidence supporting Mel56 as a PHB-binding compound and the need to rigorously demonstrate direct target engagement. Melanogenin derivatives, including Mel56, were previously identified as PHB-binding compounds, and direct interactions with PHB1 and PHB2 were demonstrated in pull-down experiments using an agarose-conjugated derivative (Djehal et al., *Eur J Med Chem*, 2018; Wang et al., *Cell Mol Life Sci*, 2020). In the present study, we did not perform additional biophysical binding analyses (e.g., SPR or ITC). Rather, we built upon the previously established evidence for the Mel56–PHB interaction and focused on evaluating its antiviral activity and downstream functional consequences. To strengthen the mechanistic framework, we conducted additional experiments examining mitochondrial modulation and NRF2 activation following Mel56 treatment (Figs. 5–7). Furthermore, PHB2 knockdown experiments demonstrated that perturbation of PHB function reduces viral NP expression and enhances mitochondrial stress responses. However, the current data do not definitively establish PHBs as the sole or direct functional targets of Mel56. Accordingly, we have revised the manuscript to avoid overstating the mechanistic conclusion and now describe PHBs as putative or functionally implicated targets based on prior binding evidence and the phenotypic similarities observed upon PHB modulation. We have also clarified the limitations of the study and acknowledge that additional biochemical and structural studies are required to further validate direct target engagement in the antiviral context.

2. The antiviral role of Mel56 against influenza A virus (H1N1/H3N2) in MDCK cells and SARS-CoV-2 in human iPSC-derived lung organoids is a promising therapeutic target. but the mechanistic claims are overstated and not experimentally demonstrated. I recommend major revision with explicit softening of mechanistic interpretations.

Response 2:

We sincerely appreciate the thoughtful evaluation and agree that the mechanisms described in the original version of the manuscript were not sufficiently supported by direct experimental evidence. In response to this concern, we have revised the manuscript

substantially to soften the mechanistic claims and to clearly distinguish experimentally supported findings from more speculative interpretations.

Specifically:

1. We performed additional experiments demonstrating that Mel56 activates NRF2, as assessed by a reporter assay, and modulates mitochondrial function, as evidenced by attenuated mitochondrial membrane potential and increased mtROS production measured by JC-1 and MitoSOX™ Red staining, respectively, in live MDCK cells (Figs. 5 and 7). These data provide experimental support for mitochondrial modulation and NRF2 activation in the IAV model.
2. We have clarified that NRF2 activation is observed in the IAV system but not in SARS-CoV-2–infected hiPSC-derived lung organoids under conditions in which Mel56 exhibits antiviral activity (Figs. 8 and S9). Thus, the contribution of NRF2 signaling appears to be cell type- and/or virus-dependent rather than a universal mechanism.
3. We have revised the manuscript to indicate that PHB modulation is associated with antiviral effects, while acknowledging that direct target engagement under antiviral conditions has not been conclusively demonstrated.

The Abstract, Importance, Results, Discussion, and Conclusion sections have been revised to temper mechanistic claims and explicitly state the limitations of the current data. We believe these revisions improve the rigor and clarity of the manuscript substantially while preserving the central finding that Mel56 exhibits antiviral activity against IAV and SARS-CoV-2 in relevant experimental systems.

Reviewer #1:

Major concerns:

Comment 1:

1. In the figure 4, knocking down PHB2 significantly decreases PR8 NP expression, indicating PHB2 helps viral replication not against IAV infection. And this article showed PHB1 facilitates PR8 IAV replication. (Zou J, Tian S, Zhu Y, Cheng Y, Jiang M, Tu S, Jin M, Chen H, Zhou H.2023. Prohibitin1 facilitates viral replication by impairing the RIG-I-like receptor signaling pathway. J Virol97:e00926-23.https://doi.org/10.1128/jvi.00926-23)

Moreover, the data of this study supports Mel56 has antiviral activities but not support this antiviral role needs PHBs.

Response 1:

We sincerely appreciate the reviewer's insightful comment regarding the role of PHBs in the antiviral activity of Mel56. In our experiments, PHB2 knockdown using DsiRNA reduced the protein levels of both PHB2 and PHB1, consistent with the interdependent stability of the PHB complex. PHB2 DsiRNA alone and Mel56 treatment each decreased IAV NP expression. Notably, the combination of PHB2 knockdown and Mel56 treatment further decreased viral NP expression, indicating an additive effect. We acknowledge the report by Zou et al. (*J Virol* 2023) demonstrating that siPHB1 transfection reduces PR8 IAV replication, indicating that PHB1 facilitates viral replication. Because PHBs are essential for maintaining mitochondrial integrity and function, their reduction by siRNA likely induces mitochondrial stress, leading to increased ROS production, as reported previously (Huang et al., *Nat Commun*, 2021). This mitochondrial stress response may activate the NRF2 pathway. NRF2 activation exerts antiviral effects, potentially explaining the reduced NP expression observed following PHB2 knockdown. Similarly, Mel56 binds to PHBs and modulates mitochondrial function, resulting in enhanced mitochondrial ROS (mtROS) production and NRF2 activation, as shown in Figs. 5–7. Mel56 also induces cell apoptosis via the loss of mitochondrial potential and the caspase activation in melanoma lines (Najem et al., Cells 2023). Therefore, although PHBs may support viral replication under basal conditions, their functional modulation—either through genetic knockdown or pharmacological targeting by Mel56—appears to induce mitochondrial stress responses that activate NRF2 signaling and suppress IAV replication. Taken together, these findings suggest that PHBs are mechanistically involved in the antiviral activity of Mel56, not because PHBs themselves are intrinsically antiviral but because the modulation of PHB-associated mitochondrial function contributes to the activation of host antiviral pathways. We have clarified these points in the revised

Discussion section:

Pages 18 and 19, lines 372–397:

"Mel56 is a synthetic triazine derivative that binds to PHB1 and PHB2 and has previously been shown to promote melanogenesis and induce apoptosis in melanoma cells [33,34]. PHBs are multifunctional scaffolding proteins implicated in the replication of several viruses, including IAV [22-25], SARS-CoV [26,27], and SARS-CoV-2 [28,29]. In IAV-infected cells, PHB is upregulated, and siRNA-mediated knockdown of PHBs reduces viral replication [22,24,25], supporting a proviral role under basal conditions. In the present study, both *PHB2* DsiRNA and Mel56 independently reduced viral NP expression, and their combination exerted a stronger suppressive effect, indicating that PHB function is mechanistically linked to viral replication and can be disrupted by Mel56. Given that PHBs are essential for maintaining mitochondrial integrity and function, their reduction by siRNA likely induces mitochondrial stress and increases ROS production, as previously reported [47]. Such mitochondrial stress may activate the NRF2 pathway, which exerts antiviral effects and may therefore explain the reduced NP expression observed following *PHB2* knockdown. Similarly, Mel56 binding to PHBs modulated mitochondrial function, resulting in enhanced mtROS production and NRF2 activation (Figs. 5–7). Mel56 also induces cell apoptosis via the loss of mitochondrial potential and the caspase activation in melanoma lines [48]. Thus, although intact PHB complexes may facilitate efficient viral replication, their functional perturbation—either through genetic knockdown or pharmacological targeting by Mel56—appears to shift mitochondrial homeostasis toward a stress-responsive state that activates NRF2 signaling and suppresses IAV replication. This interpretation reconciles the apparent paradox between the basal proviral role of PHBs and the antiviral effects observed upon their modulation. Rather than functioning as direct antiviral factors, PHBs may serve as regulatory nodes whose disruption triggers host stress responses that limit viral replication. This model aligns with the concept of host-directed antiviral strategies, in which controlled modulation of host factors activates protective signaling pathways rather than directly targeting viral components. In this context, Mel56 binding to PHBs may alter PHB-dependent mitochondrial signaling to promote NRF2-mediated antiviral responses."

Page 23, lines 482–487:

"In addition, the additive effect observed with PHB2 knockdown does not establish whether Mel56 and PHB perturbation act through identical or partially overlapping mechanisms. Thus, while our findings support a functional association between PHB modulation, mitochondrial stress responses, and antiviral activity, further biochemical

and genetic studies are required to definitively identify the direct molecular target(s) and clarify the mechanistic basis of the antiviral effects of Mel56."

Comment 2:

2. The last sentence in the first paragraph in page (line 219-222) says Mel56 may exert anti-influenza activity by suppressing host immune responses triggered by viral infections. The down-regulated innate immune genes only indicate that the antiviral function of Mel56 is by other mechanisms not by increasing the innate immune response.

Response 2:

We appreciate the reviewer's comment regarding the statement that Mel56 may exert anti-influenza activity by suppressing host immune responses triggered by viral infections. We agree that the observed downregulation of innate immune genes does not support this statement; rather, it suggests that antiviral activity is mediated through alternative mechanisms. Accordingly, we have deleted the sentence from the revised manuscript.

Comment 3:

3. The study showed Mel56 impairs mitochondrial function and transcriptome analysis indicated antioxidant gene expression is upregulated. But no experiments support the NRF2-ROS or NRF2 activation mechanism, the mechanism is speculative. Experiments like NRF2 inhibitor, activator or NRF2 siRNA, ROS assays may be need to include to have this conclusion.

Response 3:

We sincerely appreciate the reviewer's insightful comment regarding the NRF2-ROS activation mechanism. To address this concern, we have performed additional experiments to determine whether Mel56 activates NRF2 and modulates mitochondrial function in MDCK cells. Using an NRF2 reporter assay, we confirmed that Mel56 significantly activates NRF2 (Fig. 5I). Furthermore, mitochondrial membrane potential, total intracellular ROS, and mtROS production were assessed in live MDCK cells using JC-1, DCFH-DA, and MitoSOX™ Red staining, respectively. Mel56 attenuated mitochondrial membrane potential (Figs. 7A and 7B) and increased mtROS production (Figs. 7E and 7F), whereas total intracellular ROS levels were not significantly altered (Figs. 7C and 7D). These findings support the notion that Mel56 induces mitochondrial dysfunction and enhances mtROS generation, which is associated with NRF2 activation and the subsequent upregulation of antioxidant responses. We have revised the Abstract, Importance, Results, Discussion, Conclusion, and Materials and Methods sections to incorporate these new data.

Comment 4:

4. The Mitochondrial assays performed in rat liver mitochondria which is not the same species as the actual cell models (MDCK cells and human organoids) and is lack relevance to cell infection biology.

Response 4:

We sincerely appreciate the reviewer's insightful comment regarding the use of rat liver mitochondria for mitochondrial assays and their relevance to our cellular infection models. We agree that interspecific differences may limit the physiological relevance of isolated rat liver mitochondria. To address this concern, we have performed additional experiments using MDCK cells to evaluate the effects of Mel56 on NRF2 activation, mitochondrial membrane potential, intracellular ROS, and mtROS production, as described in **Response 3 to Reviewer #1, Comment 3**. These experiments confirmed that Mel56 activates NRF2, modulates mitochondrial function, and promotes mtROS production in the same cellular context used for the viral infection assays. In addition, in RT-qPCR analyses of SARS-CoV-2–infected hiPSC-derived lung organoids, expression levels of NRF2 target genes, including *NQO1* and *HMOX1*, were comparable to those in DMSO-treated infected organoids under conditions in which Mel56 exerted anti-SARS-CoV-2 activity (Figs. 8 and S9), indicating that NRF2 signaling was not significantly activated in this context. Taken together, these results demonstrate that Mel56-induced NRF2 activation occurs in a cell type- or context-dependent manner. We have revised the Abstract, Importance, Results, Discussion, Conclusion, and Materials and Methods sections to incorporate these new data.

Comment 5:

5.SARS-CoV-2 data showed Mel56 has the antiviral activity but no experiments to support it share the mechanism with IAV: no transcriptome analysis, no mitochondrial assays in organoids.

Response 5:

We sincerely appreciate the reviewer's important comment regarding whether the antiviral mechanism of Mel56 against SARS-CoV-2 is shared with that observed for IAV. We agree that the mechanistic analyses were more extensive in the IAV model. To address this concern, we have performed additional experiments using relevant cellular systems. As described in **Responses 3 and 4 to Reviewer #1, Comment 3 and 4**, Mel56 activated NRF2 and modulated mitochondrial function in MDCK cells used for viral infection assays. In SARS-CoV-2-infected hiPSC-derived lung organoids, the expression levels of

NRF2 target genes, including *NQO1* and *HMOX1*, were comparable to those in DMSO-treated infected controls under conditions in which Mel56 exhibited antiviral activity (Figs. 8 and S9), indicating that NRF2 signaling was not significantly activated in this context. These findings suggest that NRF2 activation is cell type- or virus-dependent. Therefore, the contribution of NRF2 signaling to antiviral activity may differ between IAV and SARS-CoV-2 models. Accordingly, we have revised the Discussion section to clarify that the mechanistic link between mitochondrial modulation and antiviral activity is well supported in the IAV system, whereas the contribution of NRF2 signaling in the SARS-CoV-2 model appears to be context-dependent and warrants further investigation. We have clarified these points in the revised Discussion section:

Pages 20 and 21, lines 410–433:

"Our transcriptomic analysis indicated that Mel56 upregulates several antioxidant response-related genes, including *NQO1*, *DHRS2*, *S100A14*, *NDRG1*, and *SLC2A1*. NRF2 activation by Mel56 was confirmed using a reporter assay. Mel56 also impaired mitochondrial ATP synthesis by suppressing electron transport in isolated mitochondria. In live cells, Mel56 decreased mitochondrial membrane potential and enhanced mtROS production. Collectively, these mitochondrial alterations suggest that mtROS generation may contribute to the activation of NRF2 signaling. We have previously reported that baku, a natural PHB2-binding compound with anti-influenza activity, also activates NRF2 signaling and induces the expression of antioxidant genes, including *NQO1* and *glutathione S-transferase A3* [24,30]. Baku impairs mitochondrial function and reduces lipid peroxidation via mtROS generation [53,54]. These results suggest that Mel56, similar to baku, exerts its antiviral effects via the mtROS-dependent activation of the NRF2 pathway. NRF2 activation has been linked to the suppression of SARS-CoV-2 replication and associated inflammation [45,55]. Pharmacological NRF2 activators, such as 4-octyl-itaconate, dimethyl fumarate, sulforaphane, and bardoxolone, inhibit viral replication and inflammation. SARS-CoV-2 NSP14 suppresses NRF2 activation via Sirtuin 1 inhibition [55], and NRF2 activity is diminished in the lungs of patients with COVID-19 [45]. Moreover, NRF2 activation reduces *ACE2* mRNA levels, a key receptor for SARS-CoV-2 entry, suggesting an additional antiviral mechanism [56]. However, under conditions in which Mel56 exerted anti-SARS-CoV-2 activity, it did not upregulate the expression of NRF2 target genes, including *NQO1* and *HMOX1*, in virus-infected lung organoids, indicating that NRF2 signaling was not activated in this setting. These results suggest that NRF2 activation is cell type- or virus-dependent. Accordingly, the contribution of NRF2 signaling to antiviral activity may differ between the IAV and SARS-CoV-2 models. Overall, these observations highlight the context-dependent nature of the antiviral effects

of Mel56 and underscore the complexity of host signaling pathways during viral infection.”

Minor concerns:

Comment 1:

1. In line 67 and 70, the citation of the website is not like this way.

Response 1:

Thank you for this comment. We have revised the website citation format on lines 71–72 and 74–75.

Comment 2:

2. In line 142 "similar to the effects of Baku at 0.4-25 μM (A/PR/8/34) or 3. 1-25 μM (A/WSN/33) (Fig. 1A)" what does 0.4-25 μM (A/PR/8/34) or 3.1-25 μM (A/WSN/33) mean?

Response 2:

Thank you for this comment. The values “0.4–25 μM (A/PR/8/34)” and “3.1–25 μM (A/WSN/33)” indicate the concentration ranges of baku used in the antiviral assays against each respective influenza A virus strain. Specifically, 0.4–25 μM refers to the range tested for the A/PR/8/34 strain, and 3.1–25 μM refers to the range tested for the A/WSN/33 strain.

Comment 3:

3. More detail is needed for the MOI selection rationale.

Response 3:

We thank the reviewer for this comment. The MOI used in this study was determined based on preliminary experiments to ensure sufficient viral replication while avoiding excessive cytopathic effects. We have clarified this point in the Materials and Methods section:

Pages 26 and 27, lines 550–553:

“The multiplicity of infection (MOI) used for the IAV infection experiments was determined based on preliminary studies to ensure robust and reproducible viral replication while minimizing excessive cytopathic effects under the experimental conditions.”

Page 39, lines 848–850:

“The MOI used for the SARS-CoV-2 infection experiments was determined based on

preliminary studies to ensure robust and reproducible viral replication while minimizing excessive cytopathic effects under the experimental conditions.”

Reviewer #2:

Comment:

It is interesting to discover that host melanogenin derivative Mel56, but not Mel6, is against influenza A virus and SARS-CoV-2 in vitro. It seems that the antiviral function of Mel56 is relative to mitochondrial modulation and NRF2 activation. However, the conclusions drawn are often speculative and not fully supported by the data presented. Further mechanism study is required.

Response:

We sincerely appreciate the reviewer's insightful comment regarding the mechanistic basis of the anti-influenza A virus (IAV) and SARS-CoV-2 activity of Mel56. We agree that the initial version of the manuscript did not provide sufficient experimental evidence to fully support our proposed mechanism. To address this concern, we have performed additional experiments to determine whether Mel56 activates NRF2 and modulates mitochondrial function in MDCK cells. Using an NRF2 reporter assay, we confirmed that Mel56 significantly activates NRF2 (Fig. 5I). In addition, mitochondrial membrane potential, total intracellular ROS, and mitochondrial ROS (mtROS) production were assessed in live MDCK cells using JC-1, DCFH-DA, and MitoSOX™ Red staining, respectively. Mel56 attenuated mitochondrial membrane potential (Figs. 7A and 7B) and increased mtROS production (Figs. 7E and 7F), whereas total intracellular ROS levels were not significantly altered (Figs. 7C and 7D). These results provide experimental support for mitochondrial modulation and NRF2 activation in the IAV model. In contrast, in SARS-CoV-2-infected hiPSC-derived lung organoids, RT-qPCR analysis showed that the expression levels of NRF2 target genes, including *NQO1* and *HMOX1*, were comparable to those in DMSO-treated infected controls under conditions in which Mel56 exhibited antiviral activity (Figs. 8 and S9), indicating that NRF2 signaling was not significantly activated in this context. Together, these findings indicate that while mitochondrial modulation by Mel56 is consistently observed, downstream NRF2 activation appears to be cell type- or virus-dependent. Thus, the mechanistic link between mitochondrial modulation and antiviral activity is well supported in the IAV system, whereas the contribution of NRF2 signaling in the SARS-CoV-2 model may be context-dependent and warrants further investigation. We have revised the Abstract, Importance, Results, Discussion, Conclusion, and Materials and Methods sections to incorporate these new data and clarify the mechanistic interpretation.

Comment 1:

***1.** The manuscript's cytotoxicity data raise concerns about the compound's viability. The authors state Mel56 is non-cytotoxic at <10 μM in 10% FBS media, yet Figure S1 reveals severe cytotoxicity (>90%) at just 20 μM, indicating a very steep toxicity curve. More troubling is the low CC50 (<1.25 μM) observed in 1% BSA-containing medium. This high sensitivity to serum protein concentration complicates the interpretation of its antiviral effect. The authors must clarify which medium conditions (10% FBS or 1% BSA) were used for all subsequent antiviral assays.*

Response 1:

We sincerely appreciate the reviewer's important comment regarding the culture medium conditions used in the antiviral assays and the interpretation of the cytotoxicity data. As described in the Materials and Methods section, all experiments involving Mel56—including antiviral infection assays and mechanistic analyses—were performed in growth medium containing 10% FBS consisting of high-glucose DMEM supplemented with 10% FBS, 100 U/mL penicillin, 100 μg/mL streptomycin, and 4 mM L-glutamine, **except for the Mel56 cytotoxicity assays**. Serum-free medium containing 1% BSA was used exclusively for the MTT and WST-8 cytotoxicity assays summarized in Fig. S1D and S1E and was not used for any antiviral or mechanistic experiments. In the hiPSC-derived lung organoid experiments, SARS-CoV-2 infection assays were conducted using growth medium containing 5% FBS consisting of Eagle's minimum essential medium supplemented with 5% FBS and P/S, as described in the Materials and Methods section. Under these conditions, treatment with 10 μM Mel56 only slightly reduced organoid viability (Fig. S8C). Therefore, the pronounced cytotoxicity observed in MDCK cells cultured in 1% BSA-containing medium reflects the specific conditions of the cytotoxicity assay and does not apply to the antiviral or mechanistic experiments. We have revised the Materials and Methods section to clearly state these distinctions.

***2.** The data in Figure 2 show that Mel56 treatment reduces the percentage of NP-positive cells but does not appear to diminish the NP signal intensity within cells that are already infected. This observation suggests a mechanism of action at an early stage of the viral life cycle, such as inhibition of viral entry. It is highly suggested to dissert which viral cycle step is affected by Mel56.*

Response 2:

We sincerely appreciate the reviewer's insightful comment regarding the stage of the viral life cycle affected by Mel56. We agree that the reduction in the percentage of NP-positive cells, without a marked decrease in NP signal intensity in already infected cells, suggests an effect at an early stage of infection. However, we did not perform specific time-of-

addition or viral entry assays to definitively determine which step of the viral life cycle is targeted by Mel56. Therefore, we cannot conclusively state whether Mel56 directly inhibits viral entry, uncoating, or another early replication step. We addressed the potential early-stage effect in the revised Discussion section and clarified that further mechanistic studies, such as time-of-addition and entry assays, are required to precisely define the affected step in the viral life cycle.

Page 23, lines 468–474:

"Second, the reduction in the proportion of NP-positive cells without a substantial decrease in NP signal intensity among infected cells (shown in Fig. 2) suggests that Mel56 primarily affects an early stage of the viral life cycle. This pattern is consistent with a potential impact on viral entry, uncoating, or early replication events rather than direct inhibition of viral protein synthesis in already infected cells. However, because time-of-addition and viral entry assays were not performed in this study, the precise step targeted by Mel56 remains undefined. Future experiments designed to dissect the temporal stage of viral inhibition are necessary to clarify this mechanism."

3. *The study relies exclusively on MDCK cells. While a standard model, results from a canine kidney cell line may not be representative of the Mel56's activity in the human respiratory tract, the primary site of influenza infection. To establish physiological relevance, it is essential to validate the CC50 and EC50 values in human-derived lung epithelial cell lines (e.g., A549, BEAS-2B) and preferably in primary human bronchial epithelial (HBE) cell models.*

Response 3:

We sincerely appreciate the reviewer's insightful comment regarding the validation of CC_{50} and EC_{50} values for Mel56 in human-derived lung epithelial cell models. In the revised manuscript, we have evaluated and reported the selectivity index (SI) of Mel56 against IAV and SARS-CoV-2 infection using MDCK cells, A549 cells, and hiPSC-derived lung organoids, as shown in Tables 1 and 2 and described in the Results section (see pages 9 and 10, lines 169–174; pages 16 and 17, lines 330–335). The calculation of SI values, as described in the Materials and Methods section (see pages 42 and 43, lines 917–929), was based on CC_{50} values determined through WST-8 assays using MDCK cells, A549 cells, and hiPSC-derived lung organoids (Figs. S1C, S2A, and S8C) and IC_{50} values derived from western blot and immunofluorescence analyses of A/PR/8/34, A/WSN/33, and A/Aichi/2/68 strains in MDCK or A549 cells (Figs. 1C, 1E, 1G, S2C, S2E, and S2G) or from qPCR analyses of viral RNA levels in SARS-CoV-2-infected organoids cultured in 96-well plates (Fig. S8B). In IAV infection assays using MDCK and

A549 cells, the calculated SI values ranged from approximately 0.1 to >9.9 (Table 1). In the SARS-CoV-2 infection assay using hiPSC-derived lung organoids, the SI value was approximately 2.0 (Table 2). We have revised the Results and Materials and Methods sections to clarify these points.

Pages 9 and 10, lines 169–174:

"We evaluated the selectivity index (SI) of Mel56 against IAV infection in MDCK and A549 cells, as summarized in Table 1. SI values were calculated using CC_{50} values determined by the WST-8 assay in MDCK and A549 cells (Figs. S1C and S2A) and IC_{50} values derived from WB and IF analyses of NP expression for the A/PR/8/34, A/WSN/33, and A/Aichi/2/68 strains in the respective cell lines (Figs. 1C, 1E, 1G, S2C, S2E, and S2G). The calculated SI values ranged from approximately 0.1 to >9.9 (Table 1)."

Pages 16 and 17, lines 330–335:

"We further evaluated the SI of Mel56 against SARS-CoV-2 infection in hiPSC-derived lung organoids, as shown in Table 2, using the same calculation method described in Table 1. The SI values were calculated based on CC_{50} values determined by the WST-8 assay in hiPSC-derived lung organoids (Fig. S8C) and IC_{50} values derived from a qPCR analysis of viral RNA levels in SARS-CoV-2-infected organoids cultured in 96-well plates (Fig. S8B). The calculated SI value was approximately 2.0 (Table 2)."

Pages 42 and 43, lines 917–929:

"**CC_{50}, IC_{50}, and Selectivity Index Determination**

The selectivity index (SI) was calculated using the formula: $SI = CC_{50}/IC_{50}$, where CC_{50} represents the 50% cytotoxic concentration and IC_{50} represents the 50% inhibitory concentration. CC_{50} values of Mel56 were determined by the WST-8 cell viability assay using MDCK cells, A549 cells, and hiPSC-derived lung organoids, as described above (Figs. S1C, S2A, and S8C). IC_{50} values against the infection of IAV strains A/PR/8/34, A/WSN/33, and A/Aichi/2/68 were calculated based on WB and IF analyses of viral NP expression in Mel56-treated MDCK or A549 cells infected with the respective IAV strains (Figs. 1C, 1E, 1G, S2C, S2E, and S2G). For SARS-CoV-2 infection in hiPSC-derived lung organoids, IC_{50} values were determined based on qPCR analyses of viral RNA levels (Fig. S8B). Dose–response curves were generated, and all CC_{50} and IC_{50} values were calculated using GraphPad Prism version 10 (GraphPad Software, La Jolla, CA, USA). An SI value greater than 1.0 was considered indicative of greater antiviral efficacy than cytotoxicity in the respective cell model."

4. *At Fig. 3, the authors claim that Mel56 directly reduces the expression of Ifn-β and Mx1. This conclusion is confounded by Mel56's primary antiviral activity. It is difficult to determine from the current data whether Mel56 directly suppresses the host innate immune pathway or if the reduced Ifn-β/Mx1 expression is simply a secondary consequence of the reduced viral load. To de-convolute these two possibilities, the authors need to test Mel56's effect in a virus-free system, for example, by measuring Ifn-β/Mx1 induction following stimulation with poly(I:C).*

Response 4:

We thank the reviewer for the important comment regarding our previous statement that Mel56 exerts direct effects on Ifn-β and Mx1. We agree that the observed downregulation of innate immune genes in virus-infected cells does not support the conclusion that Mel56 directly suppresses host innate immune signaling. Rather, the reduced expression of Ifn-β and Mx1 is more likely a secondary consequence of decreased viral replication. Therefore, the current data do not allow us to conclude that Mel56 exerts antiviral effects through the suppression of host immune responses. Accordingly, we have removed the following sentence from the revised manuscript:

"Their downregulation in the presence of Mel56 suggests that Mel56 may exert anti-influenza activity by suppressing host immune responses triggered by viral infections such as influenza virus, hepatitis C virus, Epstein–Barr virus, and SARS-CoV-2."

5. *At Fig. 4, the data presented are insufficient to conclude that PHB proteins are a functional target of Mel56's anti-influenza activity. If Mel56 and a separate PHB inhibition both act on same pathway, their combined effect should be redundant (non-additive). The additive effect suggested by the data would imply they act on independent targets/pathways. If to properly assess the pharmacodynamic relationship (i.e., synergistic), a checkerboard titration assay analyzing combination indices (CI) is required.*

Response 5:

We sincerely appreciate the reviewer's insightful comment regarding whether PHB proteins are functional targets of Mel56. Our additional data indicate that Mel56 binding to PHB proteins modulates mitochondrial function (Figs. 5–7). Mel56 treatment increased mtROS production and activated the NRF2 pathway (Figs. 5–7), which may be associated with the observed anti-influenza activity. Consistent with this notion, the reduction of PHB proteins via DsiRNA transfection has been reported to induce mitochondrial stress and ROS release (Huang et al., *Nat Commun*, 2021), leading to NRF2 activation and antiviral effects. In our experiments, combined treatment with Mel56 and PHB2 DsiRNA

further enhanced mtROS production and NRF2 activation, accompanied by stronger suppression of viral NP expression. As the reviewer correctly noted, the additive effect observed in our current data does not conclusively demonstrate that Mel56 and PHB inhibition act via the same pathway; there may be partial overlap or independent mechanisms. We have revised the Discussion as follows:

Pages 18 and 19, lines 372–397:

"Mel56 is a synthetic triazine derivative that binds to PHB1 and PHB2 and has previously been shown to promote melanogenesis and induce apoptosis in melanoma cells [33,34]. PHBs are multifunctional scaffolding proteins implicated in the replication of several viruses, including IAV [22-25], SARS-CoV [26,27], and SARS-CoV-2 [28,29]. In IAV-infected cells, PHB is upregulated, and siRNA-mediated knockdown of PHBs reduces viral replication [22,24,25], supporting a proviral role under basal conditions. In the present study, both *PHB2* DsiRNA and Mel56 independently reduced viral NP expression, and their combination exerted a stronger suppressive effect, indicating that PHB function is mechanistically linked to viral replication and can be disrupted by Mel56. Given that PHBs are essential for maintaining mitochondrial integrity and function, their reduction by siRNA likely induces mitochondrial stress and increases ROS production, as previously reported [47]. Such mitochondrial stress may activate the NRF2 pathway, which exerts antiviral effects and may therefore explain the reduced NP expression observed following *PHB2* knockdown. Similarly, Mel56 binding to PHBs modulated mitochondrial function, resulting in enhanced mtROS production and NRF2 activation (Figs. 5–7). Mel56 also induces cell apoptosis via the loss of mitochondrial potential and the caspase activation in melanoma lines [48]. Thus, although intact PHB complexes may facilitate efficient viral replication, their functional perturbation—either through genetic knockdown or pharmacological targeting by Mel56—appears to shift mitochondrial homeostasis toward a stress-responsive state that activates NRF2 signaling and suppresses IAV replication. This interpretation reconciles the apparent paradox between the basal proviral role of PHBs and the antiviral effects observed upon their modulation. Rather than functioning as direct antiviral factors, PHBs may serve as regulatory nodes whose disruption triggers host stress responses that limit viral replication. This model aligns with the concept of host-directed antiviral strategies, in which controlled modulation of host factors activates protective signaling pathways rather than directly targeting viral components. In this context, Mel56 binding to PHBs may alter PHB-dependent mitochondrial signaling to promote NRF2-mediated antiviral responses."

Page 23, lines 482–487:

"In addition, the additive effect observed with PHB2 knockdown does not establish whether Mel56 and PHB perturbation act through identical or partially overlapping mechanisms. Thus, while our findings support a functional association between PHB modulation, mitochondrial stress responses, and antiviral activity, further biochemical and genetic studies are required to definitively identify the direct molecular target(s) and clarify the mechanistic basis of the antiviral effects of Mel56."

6. *Fig. 5D shows that the expression levels of VGF, DHRS2, S100A14, NDRG1, DHRS9, SLC2A1, ITGA3, and NQO1 were significantly upregulated by Mel56. However, the conclude that 'Mel56 attenuates virus-induced immune responses and enhances antioxidant gene expression, potentially via NRF2 activation' is correlative and speculative. To link this pathway to the antiviral effect, its necessity must be demonstrated. The authors should test if the antiviral activity of Mel56 is abrogated in Nrf2 knockout/knockdown (KO/KD) cells or in the presence of an NRF2 inhibitor.*

Response 6:

We sincerely appreciate the reviewer's insightful comment regarding the mechanistic basis of the anti-IAV and SARS-CoV-2 activity of Mel56. We agree that the initial version of the manuscript did not provide sufficient experimental evidence to fully substantiate the proposed mechanism. To address this concern, we have performed additional experiments to examine whether Mel56 activates NRF2 and modulates mitochondrial function in MDCK cells. Using an NRF2 reporter assay, we confirmed that Mel56 significantly activates NRF2 (Fig. 5I). Furthermore, mitochondrial membrane potential, total intracellular ROS, and mtROS production were assessed in live MDCK cells using JC-1, DCFH-DA, and MitoSOX™ Red staining, respectively. Mel56 reduced mitochondrial membrane potential (Figs. 7A and 7B) and increased mtROS production significantly (Figs. 7E and 7F), whereas total intracellular ROS levels were not significantly altered (Figs. 7C and 7D). These findings provide experimental evidence that Mel56 modulates mitochondrial function and activates NRF2 signaling in the IAV model. In contrast, in SARS-CoV-2-infected hiPSC-derived lung organoids, RT-qPCR analysis demonstrated that the expression levels of NRF2 target genes, including *NQO1* and *HMOX1*, were comparable to those in DMSO-treated infected controls under conditions in which Mel56 exhibited antiviral activity (Figs. 8 and S9). These results suggest that NRF2 signaling was not significantly activated in this context. Together, these findings indicate that Mel56 consistently results in mitochondrial modulation, whereas downstream NRF2 activation appears to be cell type- and/or virus-dependent. While our data support an association between mitochondrial modulation, NRF2

activation, and antiviral activity in the IAV system, we acknowledge that the necessity of NRF2 signaling for the antiviral effect has not yet been formally demonstrated (e.g., using NRF2 knockout/knockdown models or pharmacological inhibitors). Accordingly, we have revised the Abstract, Importance, Results, Discussion, Conclusion, and Materials and Methods sections to temper the mechanistic interpretation and to clearly state the limitations of the current study.

7. Figure 6 indicates that Mel56 disrupts mitochondrial function. Disruption of mitochondrial integrity is a common hallmark of cell poisoning. The authors must provide evidence to decouple this mitochondrial dysfunction from non-specific cytotoxicity and demonstrate that this effect is specific to inhibiting the virus, not killing the host cell.

Response 7:

We sincerely appreciate the reviewer's important comment that the disruption of mitochondrial integrity is commonly associated with nonspecific cytotoxicity. We agree that it is essential to distinguish virus-specific antiviral effects from general host-cell toxicity. To address this concern, we have performed additional experiments to evaluate mitochondrial function and cell viability under conditions in which Mel56 exhibited antiviral activity. First, cell viability was assessed using the WST-8 assay, and the concentrations of Mel56 used for mechanistic analyses were confirmed to be below the CC_{50} values in MDCK cells cultured in growth medium containing 10% FBS, indicating that the observed effects were not attributable to overt cytotoxicity (Fig. S1C and Table 1). We then examined whether Mel56 at a non-toxic concentration (10 μM) modulates mitochondrial function in live MDCK cells. Using an NRF2 reporter assay, we confirmed that Mel56 (10 μM) significantly activates NRF2 (Fig. 5I). Furthermore, mitochondrial membrane potential, total intracellular ROS, and mitochondrial ROS (mtROS) production were assessed using JC-1, DCFH-DA, and MitoSOX™ Red staining, respectively. Mel56 (10 μM) reduced mitochondrial membrane potential moderately (Figs. 7A and 7B) and increased mtROS production significantly (Figs. 7E and 7F), whereas total intracellular ROS levels were not significantly altered (Figs. 7C and 7D). Importantly, these mitochondrial changes occurred under conditions in which overall cell viability was largely preserved, suggesting that Mel56 modulates mitochondrial signaling in a controlled manner, rather than inducing nonspecific toxicity. A previous study also reported that Mel56 does not exhibit overt cytotoxicity in preadipocytes at 10 μM (Gao et al., *Diabetes*, 2021). Together, these findings support the interpretation that mitochondrial modulation by Mel56 is mechanistically associated with antiviral activity and not a secondary consequence of generalized cytotoxicity.

8. *The manuscript proposes two separate mechanisms (NRF2 activation and mitochondrial disruption) without a unifying link. A hypothesis mentioned at the discussion section is that mitochondrial disruption by Mel56 induces mitochondrial ROS (mtROS), which is a known upstream activator of the NRF2 pathway. It is strongly suggested to directly measure mtROS levels (e.g., via MitoSOX) following Mel56 treatment to test this hypothesis and connect the observations from Figures 5 and 6.*

Response 8:

We sincerely appreciate the reviewer's insightful suggestion to assess mtROS levels directly in order to establish a mechanistic link between mitochondrial modulation and NRF2 activation. As recommended, we have performed additional experiments to measure mtROS production following Mel56 treatment. Using MitoSOX™ Red staining in live MDCK cells, we observed a significant increase in mtROS levels upon treatment with Mel56 (Figs. 7E and 7F). In parallel, mitochondrial membrane potential was reduced moderately (Figs. 7A and 7B), whereas total intracellular ROS levels measured by DCFH-DA were not significantly altered (Figs. 7C and 7D). These findings indicate that Mel56 preferentially enhances mtROS. Importantly, activation of NRF2 by Mel56 was confirmed using an NRF2 reporter assay (Fig. 5I). Taken together, these data support the hypothesis proposed in the Discussion that the Mel56-induced modulation of mitochondrial function leads to increased mtROS production, which may act as an upstream signal for NRF2 activation. Thus, the additional mtROS measurements provide a mechanistic link connecting the mitochondrial effects (Figs. 6 and 7) with NRF2 pathway activation (Fig. 5). We have revised the Results and Discussion sections to incorporate these new data and to clearly describe mtROS as a potential upstream mediator linking mitochondrial modulation and NRF2 activation.

Re: Spectrum03093-25R1 (**Host-Directed Antiviral Strategy Targeting Prohibitins: Mel56 Suppresses Influenza A Virus and Severe Acute Respiratory Syndrome Coronavirus 2 via Modulation of Antioxidant Pathways and Mitochondrial Function**)

Dear Prof. Takashi Kuzuhara:

I have received the reviews of your manuscript and regret to inform you that we will not be able to publish it in Microbiology Spectrum. Your submission was read by reviewers with expertise in the area addressed in your study and it was the consensus view of the reviewer and editor that your paper did not meet the standards necessary for publication. I have appended the comments of the reviewers in order for you to understand the basis for the final decision.

I am sorry to convey a negative decision on this occasion, but I hope that the enclosed reviews are useful. Please note, rejections from Microbiology Spectrum are final and your manuscript will not be considered by other ASM journals. We wish you well in publishing this report in another journal and hope that you will consider Spectrum in the future.

The ASM Journals program strives for constant improvement in our submission and publication process. Please tell us how we can improve your experience by taking this quick Author Survey.

Sincerely,
Yunyu Chen
Editor, Microbiology Spectrum

Reviewer #1 (Comments for the Author):

The study includes RNA-seq (transcriptomic) datasets, which should be deposited in a public repository such as GEO or SRA. Please provide accession numbers prior to publication.

The authors have made substantial efforts to address every previous comment, including additional experiments, revision of lots of sentences in manuscripts and the adding of details. Despite these great improvements, I found a very serious problem is that all IAV infection in this study using the culture media with 10%FBS. But the successful IAV infection should be serum free. And if the IAV is propagated from cells, TPCK-trypsin is required. Thus, I suspect the reliability of all data from IAV infection.

The use of non-standard infection conditions of influenza A virus (e.g., high serum without exogenous protease) raises fundamental concerns regarding the reliability of the antiviral assays of IAV. Therefore, I do not believe the current study meets the standard required for publication.

Reviewer #2 (Comments for the Author):

The authors have addressed my concerns.

To authors

The authors have made substantial efforts to address every previous comment, including additional experiments, revision of lots of sentences in manuscripts and the adding of details. Despite these great improvements, I found a very serious problem is that all IAV infection in this study using the culture media with 10%FBS. But the successful IAV infection should be serum free. And If the IAV is propagated from cells, TPCK-trypsin is required. Thus, I suspect the reliability of all data from IAV infection.

To editor,

The use of non-standard infection conditions of influenza A virus (e.g., high serum without exogenous protease) raises fundamental concerns regarding the reliability of the antiviral assays of IAV. Therefore, I do not believe the current study meets the standard required for publication.

Responses to Reviewer's comments

Manuscript Number: Spectrum03093-25R1

Masaki Shoji, Rina Hashimoto, Canan G. Nebigil, Kazuto Takegawa, Itsuki Tomita, Kensuke Nakaoka, Momiji Ishikawa, Yasufumi Matsumura, Miki Nagao, Tomoyuki Esumi, Etsuhisa Takahashi, Hiroshi Kido, Yasuo Shinohara, Laurent Désaubry, Kazuo Takayama and Takashi Kuzuhara

"**Host-Directed Antiviral Strategy Targeting Prohibitins: Mel56 Suppresses Influenza A Virus and Severe Acute Respiratory Syndrome Coronavirus 2 via Modulation of Antioxidant Pathways and Mitochondrial Function**"

Dear Dr. Yunyu Chen,

We would like to thank you and the reviewers for the positive comments on our manuscript, "**Host-Directed Antiviral Strategy Targeting Prohibitins: Mel56 Suppresses Influenza A Virus and Severe Acute Respiratory Syndrome Coronavirus 2 via Modulation of Antioxidant Pathways and Mitochondrial Function**," which we would like to resubmit for publication. We appreciated the constructive suggestions provided by the reviewers. Based on this feedback, we have revised our manuscript, including the experimental conditions used for influenza A virus (IAV) infection, as raised by Reviewer #1.

Please find our point-by-point responses (in blue) to the comments of the reviewers (in ***italics***) below.
We believe that the revised manuscript addresses the comments provided by the reviewers and we hope that it is now suitable for publication in *Microbiology Spectrum*.

Thank you for your consideration. I look forward to hearing from you.

Sincerely yours,

Takashi Kuzuhara, Ph.D.
Laboratory of Biochemistry, Faculty of Pharmaceutical Sciences,
Tokushima Bunri University
Yamashirocho, Tokushima 770-8514, Japan
Tel.: +81 (88) 602-8477, Fax: +81 (88) 655-3051, E-mail: kuzuhara@ph.bunri-u.ac.jp

Reviewer #1 comments:

1. The study includes RNA-seq (transcriptomic) datasets, which should be deposited in a public repository such as GEO or SRA. Please provide accession numbers prior to publication.

Response 1:

We thank the reviewer for this comment. The RNA-seq data had already been deposited in the Gene Expression Omnibus (GEO) under accession number GSE304376, and this information was provided in the manuscript (Page 44, Line 958). For emphasizing this information, GEO number was also added in Methods section (Page 32, Line 693).

2. The authors have made substantial efforts to address every previous comment, including additional experiments, revision of lots of sentences in manuscripts and the adding of details. Despite these great improvements, I found a very serious problem is that all IAV infection in this study using the culture media with 10%FBS. But the successful IAV infection should be serum free. And if the IAV is propagated from cells, TPCK-trypsin is required. Thus, I suspect the reliability of all data from IAV infection. The use of non-standard infection conditions of influenza A virus (e.g., high serum without exogenous protease) raises fundamental concerns regarding the reliability of the antiviral assays of IAV. Therefore, I do not believe the current study meets the standard required for publication.

Response 2:

We sincerely appreciate the important comment regarding all IAV infection in this study using the culture media with 10% FBS absence of TPCK-trypsin. We respectfully believe that this concern reflects a misunderstanding of the experimental design and of commonly used infection conditions for host-directed antiviral studies.

1. The use of serum-containing medium (e.g., 10% FBS) for infection assays in epithelial cell systems such as MDCK and A549 cells is widely described in the literature, including established methodological references such as Influenza Virus: Methods and Protocols (Kawaoka and Neumann, eds., Springer protocols, Methods in Molecular Biology 865, Humana Press). Such conditions are frequently used when evaluating host-directed antiviral responses over extended incubation periods.
2. Because the primary objective of this study was to evaluate host-directed antiviral responses—including mitochondrial function and antioxidant pathway regulation—maintaining physiologically relevant culture conditions was essential. Serum deprivation itself is known to alter cellular metabolism and stress-response pathways

and could therefore confound interpretation of host-targeted antiviral mechanisms. For this reason, serum-containing medium was intentionally used to ensure biological interpretability of host-response readouts.

3. With respect to TPCK-trypsin, we would like to clarify that exogenous protease supplementation is primarily required for efficient multi-cycle viral propagation. In contrast, our experimental design was intended to evaluate host-directed antiviral effects under conditions compatible with single-cycle or limited-cycle infection rather than classical multi-cycle amplification assays. Under such conditions, initial infection and early replication can proceed via activation of hemagglutinin by endogenous host proteases expressed in epithelial cell systems such as A549 cells.

Consistent with this understanding, several airway epithelial cell models are known to support productive IAV infection without exogenous trypsin supplementation under appropriate experimental settings. Importantly, in our study, untreated control cells reproducibly supported viral replication sufficient for quantitative antiviral evaluation, confirming that infection efficiency under our conditions was appropriate for the objectives of this work. Comparable infection conditions without exogenous trypsin have also been reported in studies investigating host–virus interactions in epithelial systems (e.g., Matrosovich et al., PNAS 2004; Endo et al. J Virol 1994; Slepushkin et al. Mol Ther 2001). We also note that this specific concern regarding infection conditions was not raised during the first round of review. Had it been raised earlier, we would have gladly clarified the rationale and supporting literature at that stage.

We added supporting references and clarifications into the manuscript in Methods section:

Page 26 and 27, Line 544–560

“**Influenza A virus (IAV) strains**

The IAV strains A/PR/8/34 (H1N1), A/WSN/33 (H1N1), and A/Aichi/2/68 (H3N2) were provided by Takahashi and Kido [64]. These viruses were propagated in MDCK cells using serum-free medium supplemented with 3 μg/mL L-tosylamido-2-phenyl ethyl chloromethyl ketone (TPCK)-treated trypsin (Sigma-Aldrich). These culture media were clarified by centrifugation (5,000 rpm, 10 min) and stored at −80°C. Viral titers in these media were determined by immunostaining for influenza A viral NP, as previously described [30,65,66]. The multiplicity of infection (MOI) used for the IAV infection experiments was determined based on preliminary studies to ensure robust and reproducible viral replication while minimizing excessive cytopathic effects under the experimental conditions. All IAV infection experiments described below were performed

in growth medium containing 10% FBS without exogenous trypsin (e.g., TPCK-treated trypsin), as previous reports using similar culture conditions demonstrated efficient proteolytic activation of influenza viruses by endogenous proteases [67-69]. With regard to TPCK-treated trypsin, supplementation with this exogenous protease is primarily required for efficient multi-cycle viral propagation. In contrast, our experimental design was intended to evaluate host-directed antiviral effects under conditions compatible with single-cycle or limited-cycle infection, rather than classical multi-cycle amplification assays."

Re: Spectrum03093-25R2-A (**Host-Directed Antiviral Strategy Targeting Prohibitins: Mel56 Suppresses Influenza A Virus and Severe Acute Respiratory Syndrome Coronavirus 2 via Modulation of Antioxidant Pathways and Mitochondrial Function**)

Dear Prof. Takashi Kuzuhara:

Your manuscript has been accepted, and I am forwarding it to the ASM production staff for publication. Your paper will first be checked to make sure all elements meet the technical requirements. ASM staff will contact you if anything needs to be revised before copyediting and production can begin. Otherwise, you will be notified when your proofs are ready to be viewed.

Data Availability: ASM policy requires that data be available to the public upon online posting of the article, so please verify all links to sequence records, if present, and make sure that each number retrieves the full record of the data. If a new accession number is not linked or a link is broken, provide production staff with the correct URL for the record. If the accession numbers for new data are not publicly accessible before the expected online posting of the article, publication may be delayed; please contact ASM production staff immediately with the expected release date.

Publication Fees: For information on publication fees and which article types have charges, please visit our website. We have partnered with Copyright Clearance Center (CCC) to collect author charges. If fees apply to your paper, you will receive a message from no-reply@copyright.com with further instructions. For questions related to paying charges through RightsLink, please contact CCC at ASM_Support@copyright.com or toll free at +1-877-622-5543. CCC makes every attempt to respond to all emails within 24 hours.

ASM Membership: Corresponding authors may join or renew ASM membership to obtain discounts on publication fees. Need to upgrade your membership level? Please contact Customer Service at Service@asmusa.org.

Embargo Policy: A press release may be issued as soon as the manuscript is posted on the Spectrum Latest Articles webpage. The corresponding author will receive an email with the subject line "ASM Journals Author Services Notification" when the article is available online.

The ASM Journals program strives for constant improvement in our submission and publication process. Please tell us how we can improve your experience by taking this quick Author Survey.

Thank you for submitting your paper to Spectrum.

Sincerely,
Yunyu Chen
Editor
Microbiology Spectrum
